# Bluetongue Virus NS4 Protein Is an Interferon Antagonist and a Determinant of Virus Virulence

**DOI:** 10.1128/JVI.00422-16

**Published:** 2016-05-12

**Authors:** Maxime Ratinier, Andrew E. Shaw, Gerald Barry, Quan Gu, Luigina Di Gialleonardo, Anna Janowicz, Mariana Varela, Richard E. Randall, Marco Caporale, Massimo Palmarini

**Affiliations:** a MRC—University of Glasgow Centre for Virus Research, Glasgow, Scotland, United Kingdom; b Veterinary Sciences Centre, School of Veterinary Medicine, University College Dublin, Dublin, Ireland; c Istituto Zooprofilattico Sperimentale dell'Abruzzo e Molise G. Caporale, Teramo, Italy; d Biomedical Sciences Research Complex, University of St. Andrews, North Haugh, St. Andrews, United Kingdom; Instituto de Biotecnologia/UNAM

## Abstract

Bluetongue virus (BTV) is the causative agent of bluetongue, a major infectious disease of ruminants with serious consequences to both animal health and the economy. The clinical outcome of BTV infection is highly variable and dependent on a variety of factors related to both the virus and the host. In this study, we show that the BTV nonstructural protein NS4 favors viral replication in sheep, the animal species most affected by bluetongue. In addition, NS4 confers a replication advantage on the virus in interferon (IFN)-competent primary sheep endothelial cells and immortalized cell lines. We determined that in cells infected with an NS4 deletion mutant (BTV8ΔNS4), there is increased synthesis of type I IFN compared to cells infected with wild-type BTV-8. In addition, using RNA sequencing (RNA-seq), we show that NS4 modulates the host IFN response and downregulates mRNA levels of type I IFN and interferon-stimulated genes. Moreover, using reporter assays and protein synthesis assays, we show that NS4 downregulates the activities of a variety of promoters, such as the cytomegalovirus immediate-early promoter, the IFN-β promoter, and a promoter containing interferon-stimulated response elements (ISRE). We also show that the NS4 inhibitory activity on gene expression is related to its nucleolar localization. Furthermore, NS4 does not affect mRNA splicing or cellular translation. The data obtained in this study strongly suggest that BTV NS4 is an IFN antagonist and a key determinant of viral virulence.

**IMPORTANCE** Bluetongue is one of the main infectious diseases of ruminants and is caused by bluetongue virus (BTV), an arthropod-borne virus transmitted from infected to susceptible animals by Culicoides biting midges. Bluetongue has a variable clinical outcome that can be related to both virus and host factors. It is therefore critical to understand the interplay between BTV and the host immune responses. In this study, we show that a nonstructural protein of BTV (NS4) is critical to counteract the innate immune response of the host. Infection of cells with a BTV mutant lacking NS4 results in increased synthesis of IFN-β and upregulation of interferon-stimulated genes. In addition, we show that NS4 is a virulence factor for BTV by favoring viral replication in sheep, the animal species most susceptible to bluetongue.

## INTRODUCTION

Bluetongue virus (BTV) is the causative agent of bluetongue, a major infectious disease of ruminants with serious consequences for both animal health and the economy (1). Throughout the 20th century, bluetongue occurred almost exclusively in tropical and subtropical geographical areas (2). However, in the last 2 decades, the geographical limits of the disease have expanded, and BTV is now endemic in more temperate areas, such as southern and central Europe (3).

BTV is an arbovirus transmitted by Culicoides sp. biting midges and belongs to the genus Orbivirus within the Reoviridae (4, 5). The BTV genome consists of 10 double-stranded RNA (dsRNA) genome segments encoding seven structural and four, possibly five, nonstructural proteins (6–9). The core particle, formed by VP3 and VP7, encapsidates the viral genome segments, each associated with a replicase complex comprising VP1, VP4, and VP6 (6, 10–12). VP2 and VP5 constitute the outer capsid of the BTV virion and are responsible for cell attachment and entry (13–15). VP2 is the most variable BTV protein and determines the virus serotype, 27 of which have been described to date (16–19).

The BTV nonstructural proteins play fundamental roles in virus replication. NS1 forms tubules in the cytoplasm of BTV-infected cells and favors viral protein synthesis (20–22). NS2 is the major component of viral inclusion bodies (23–25), while NS3/NS3A play a critical role in virus intracellular trafficking and egress (26, 27). NS3 has also been shown to downregulate transcription from the beta interferon (IFN-β) promoter in reporter assays (28). A putative fifth nonstructural protein may be expressed from a conserved small open reading frame (ORF) in segment 10 (29).

NS4 is a small protein (77 to 79 amino acid residues) encoded by an ORF in genome segment 9 overlapping the larger ORF encoding VP6 and with nucleolar localization. Although NS4 has been shown to confer a replication advantage on BTV in cells treated with interferon, it is dispensable for BTV replication in IFN-incompetent cell lines and has no impact on pathogenicity in IFN-α/β receptor^−/−^ (IFNAR^−/−^) mice (8, 9).

The clinical outcome of BTV infection is highly variable and dependent upon a variety of viral, host, and, likely, environmental factors (30–35). Understanding the interplay between BTV and the host immune response will be central to identifying the virus and host determinants of disease susceptibility.

In order to establish a successful infection, BTV must overcome both physical and innate immune barriers. One of the key innate immune mechanisms for fighting viral infections is the IFN system (36). Mammalian cells possess pattern recognition receptors that detect incoming pathogen signatures and induce the synthesis and secretion of IFN-β. Secreted IFN signals in both autocrine and paracrine fashions, leading to the transcription of hundreds of IFN-stimulated genes (ISGs), some of which have a direct or indirect antiviral effect (37). BTV is known to induce an IFN response, both *in vitro* and *in vivo*, and consequently, it must possess countermeasures that allow the virus to replicate in the face of this host response (8, 38–43). Evidence to date suggests that BTV NS3 and NS4 are the viral proteins most likely responsible for the disruption of the cellular innate immune response to BTV infection (8, 28).

In this study, we investigated the interaction of BTV and the IFN response of the host. We demonstrate that NS4 is an important virulence factor in sheep, a natural host of BTV infection, and acts as an interferon antagonist.

## MATERIALS AND METHODS

### Cell cultures.

Vero, BHK21, and BSR (a clone of BHK21, kindly provided by Karl K. Conzelmann) (44) cells were grown in Dulbecco's modified Eagle's medium (DMEM) supplemented with 10% fetal bovine serum (FBS). CPT-Tert cells are sheep choroid plexus cells immortalized with the simian virus 40 (SV40) T antigen and human telomerase reverse transcriptase (hTERT) (45). CPT-Tert cells were grown in Iscove's modified Dulbecco's medium (IMDM) supplemented with 10% FBS. A549 cells are derived from a human lung adenocarcinoma and were grown in DMEM supplemented with 10% FBS. All cell lines were cultured at 37°C in a 5% CO_2_ humidified atmosphere.

Primary ovine endothelial cells (ovEC) were obtained as previously described (46). ovEC were seeded in 12-well plates and maintained in a low-oxygen incubator (37°C, 5% CO_2_, and 3% O_2_). In this study, ovEC were passaged once before being used and cultured at 37°C in a 5% CO_2_ humidified atmosphere.

### Viruses.

Wild-type BTV8 (BTV8wt) and an NS4 deletion mutant (BTV8ΔNS4) used in this study were obtained by reverse genetics as described previously (8, 47). Virus stock titers were determined by standard plaque assays using CPT-Tert cells (48). The defective-interfering (DI)-rich preparation of Sendai virus (SeV) (Cantell strain) was generated by sequentially passaging the virus at a high multiplicity of infection (MOI), as previously described (49). Encephalomyocarditis virus (EMCV) was used in interferon protection assays (see below) and was prepared as previously described (50).

### Virus replication curves.

Replication curves were carried out in either ovEC or A549 cells. The cells were infected with the appropriate virus (MOI = 0.01), and the supernatants were collected at 24, 48, and 72 h postinfection (p.i.). The supernatants were subsequently titrated by endpoint dilution analysis on BSR cells using the method of Reed and Muench and expressed as log_10_ 50% tissue culture infective doses (TCID_50_)/ml (51). Each experiment was performed independently in triplicate using at least two different stocks of each virus.

### Ethical statement.

All animal experimental procedures carried out in this study were approved by the ethical committee of the Istituto Zooprofilattico Sperimentale dell'Abruzzo e Molise G. Caporale (Teramo, Italy) (protocol no. 11427/2012) and further approved by the Italian Ministry of Health (Ministero della Salute) in accordance with Council Directive 86/609/EEC of the European Union and the Italian D.Igs 116/92.

### *In vivo* pathogenicity studies.

Experiments were carried out using 15 sheep (Italian mixed breed) in an insect-proof isolation unit. Before inoculation, all the animals were confirmed to have no antibodies against BTV using a blocking enzyme-linked immunosorbent assay (ELISA) as previously described (52). The absence of BTV genome in blood samples from each animal was also confirmed by quantitative reverse transcription-PCR (qRT-PCR) as described previously (30). The animals (*n* = 5 per group) were infected with 5 ml of either BTV8wt or BTV8ΔNS4 (2 × 10^6^ PFU in total) by multiple intradermal inoculations in the inner leg and prescapular areas. Negative controls were inoculated with 5 ml of mock-infected cell supernatants. Body temperature was recorded daily, beginning a week before inoculation, until day 14 p.i. and subsequently at days 17, 21, and 28. Fever was defined as a rectal temperature above 40°C. EDTA blood samples were collected daily from all the animals for 14 days p.i. and thereafter at days 17, 21, and 28, when the experiment was terminated. The blood samples were analyzed for the presence of viremia by qRT-PCR as previously described (30). Sera were collected from each animal on the day of the inoculation (day 0) and then at days 7, 14, 21, and 28 p.i. The presence in infected sheep of neutralizing antibodies against BTV8 was assessed by virus neutralization assay as previously described (53).

### Plasmids and antisera.

The open reading frame encoding NS4 was either amplified by PCR [BTV8 NET2006/04 (GenBank accession number JX680455), BTV1 RSArrrr/01 (JX680465), and BTV-2IT(L) (JN255870)] or synthesized commercially (Genscript) [(BTV-2RSA(WT) (JN255930), BTV-9IT(L) (JN255910), BTV1SASEG9 (D10905), BTV25 (EU839845), and BTV26 (JN255161)] and cloned into the pCI mammalian expression vector (Promega). BTV10 segment 8 (NC_006007) was synthesized commercially (Genscript) and cloned into pCI. All plasmids used in this study were sequenced before use. Plasmid pCMV-luc was obtained by inserting the firefly luciferase (FLuc) open reading frame into pCDNA3.1 (Invitrogen) as previously described (54). pRL-CMV (Promega) is similar to pCMV-luc but contains an intron before the Renilla luciferase (RLuc) gene. p125Luc expresses FLuc under the control of the IFN-β promoter (55). pISRE-Luc (Promega) contains five copies of the interferon-stimulated response element (ISRE)-binding sequence (56) located upstream of the TATA-like promoter region from the herpes simplex virus thymidine kinase (HSV-TK) promoter.

Antisera used in this study included polyclonal rabbit antisera raised against the BTV NS4 and NS2 proteins, as previously described (8). Antibodies against B23 (Sigma), α-tubulin (Sigma), IRF-3 (clone FL-425; Santa Cruz), and NF-κB p65 (clone D14E12; Cell Signaling) were obtained commercially.

### *In vitro* RNA transcription.

Capped and polyadenylated RNA for use in luciferase assays was generated using the mMessage mMachine T7 Ultra kit as recommended by the manufacturer's instructions (Ambion), using linearized pCMV-luc or pRL-CMV as a template.

An RNA control (named EU-Luc-pA) for use in transcriptome sequencing (RNA-seq) and qRT-PCR experiments was obtained as follows. Linearized pCMV-luc was transcribed using the Megascript T7 kit (Ambion, Life Technologies) according to the manufacturer's instructions, with the exception that the transcription reaction mixture was supplemented with an analogue of uridine (5-ethynyl uridine [EU]; Invitrogen, Life Technologies). The RNA was then polyadenylated following the poly(A) tailing procedure of the mMessage mMachine T7 Ultra kit. All *in vitro*-transcribed RNA was recovered using the RNeasy minikit (Qiagen).

### Labeling and extraction of nascent RNA for transcriptomic analyses.

A549 cells were seeded in 6-well plates, incubated at 37°C for 24 h, and mock infected or infected with BTV8wt or BTV8ΔNS4 at an MOI of 4. At 8, 12, and 16 h p.i., nascent RNA was labeled with 0.4 mM EU for 90 min. The cells were lysed with 1 ml of TRIzol spiked with 1 ng of EU-Luc-pA. Total RNA was then extracted using the TRIzol method and further purified using RNeasy mini spin columns (Qiagen), including an on-column DNase I digestion step (Qiagen), according to the manufacturer's protocol.

### RNA-seq.

Total RNA (4.5 μg) was enriched by selectively depleting rRNA using the RiboMinus Eukaryote kit v2 (Ambion, Life Technologies). The EU-labeled RNA was specifically linked to azide-modified biotin by a chemical reaction and then captured on streptavidin magnetic beads using the Click-iT Nascent RNA Capture kit (Molecular Probes, Life Technologies). Biotin-labeled RNA (attached to the magnetic beads) was fragmented and subsequently used to construct libraries using the Ion Total RNA-seq kit v2 (Life Technologies) as described by the manufacturer. The amplified libraries were size selected using the E-Gel size select system (Life Technologies) and assessed using a Tapestation (Agilent). The libraries were quantified using the Qubit HS dsDNA assay (Life Technologies) and sequenced using the Ion Proton sequencer (Life Technologies). The sequence reads were processed according to the Tuxedo pipeline (57). The read quality was first assessed using FastQC (58). Tophat2 and Bowtie2 were used to map short reads against the Homo sapiens genome (genome browser UCSC hg19), and a list of differentially expressed genes was generated using CuffDiff2 (genes with Benjamini Hochberg *P* values of ≤0.05 were considered significant) (59, 60). Canonical pathway analysis was performed using the Ingenuity Pathway Analysis (IPA) software (Qiagen) by submitting the differentially expressed (DE) gene data sets (see Tables S1 to S3 in the supplemental material) and using the default parameters.

### qRT-PCR.

For qRT-PCR analysis, mRNA was purified from the total RNA fraction using the Dynabeads mRNA Direct kit (Ambion, Life Technologies) according to the manufacturer's instructions before capturing EU-labeled RNA, as described above. RNA captured on the streptavidin beads was used directly as a template for cDNA synthesis using the SuperScript Vilo cDNA synthesis kit (Invitrogen, Life Technologies). qPCR was performed using Brilliant III Ultra Fast QPCR master mix (Agilent) to detect a selection of ISGs and housekeeping genes and the spiked EU-Luc-pA. The sequences of primers and probes are available upon request. Samples were run on an Mx3005P (Stratagene) PCR machine and analyzed using MxPro software (Stratagene). Mock-infected cells were used as a calibrator against which the infected cells were compared. The EU-Luc-pA RNA was used as an exogenous control to normalize the results.

### Interferon protection assays.

Interferon protection assays were performed as previously described (46). Briefly, ovEC were first seeded in 12-well plates and incubated for 2 days. The cells were then mock infected or infected with the indicated virus at an MOI of 4, and the medium was collected 16 h postinfection. Cell culture supernatants were treated with UV light to inactivate any virus. CPT-Tert cells were incubated with 2-fold dilutions of the medium for 24 h before infecting them with EMCV for 72 h. The level of IFN (expressed as international units [IU]) was calculated by monitoring wells that were protected from cell death induced by EMCV and comparing them to known amounts of universal type I IFN alpha (UIFN; PBL Assay Science).

### Western blotting.

Protein expression was assessed from total cell lysates by sodium dodecyl-sulfate polyacrylamide gel electrophoresis (SDS-PAGE) and Western blotting using the various antisera indicated above as previously described (61). For quantitative Western blotting, primary antibodies were detected using fluorescently labeled secondary antibodies (DyLight; Thermo Scientific) in a LI-COR Odyssey scanner. Bands were quantified with the Odyssey software (LI-COR Biosciences).

### Metabolic radiolabeling.

ovEC were mock infected or infected with BTV8wt or BTV8ΔNS4 at an MOI of 4. At the time points indicated, cells were incubated at 37°C for 30 min in medium lacking methionine. Nascent proteins were labeled for 2 h with [^35^S]methionine/cysteine (0.8 MBq/ml; PerkinElmer), after which they were resuspended in sample buffer (60 mM Tris-HCl [pH 6.8], 2% SDS, 10% glycerol, 2.5% β-mercaptoethanol, 0.01% bromophenol blue), and the proteins were separated by SDS-PAGE as described above. Visualization of labeled proteins was achieved by exposure to a Storm 840 PhosphorImager (Molecular Dynamics) or to X-ray film. Band intensities were determined using ImageQuant software (Amersham).

### Luciferase assays.

CPT-Tert cells grown in 24-well plates were cotransfected using Lipofectamine 2000 (Invitrogen) with 100 ng of pCMV-luc or pRL-CMV, along with 100 to 400 ng of the indicated pCI plasmids expressing NS4. A similar procedure was performed to cotransfect 200 ng of *in vitro*-transcribed RNA expressing firefly luciferase (see above) and the appropriate expression plasmids, as indicated (100 to 400 ng). When using p125Luc (50 ng) and pISRE-luc (400 ng), 293T cells were either infected with DI-rich preparations of SeV or treated with 200 U/ml UIFN (PBL InterferonSource) 4 h posttransfection before incubating for a further 18 h.

The total amount of plasmid DNA transfected was 500 ng for all experiments, using the empty pCI plasmid to balance each transfection. Luciferase activity was detected 22 h posttransfection, using a luciferase or dual-luciferase assay system (Promega) as described by the manufacturer. The percentage of luciferase was determined by setting the luciferase activity or luminescence (expressed as relative light units [RLU]) detected in control cells (i.e., transfected with pCMV-luc and an empty pCI plasmid only) as 100%. Constitutive promoters could not be used as transfection controls, as NS4 affects gene expression. However, each experiment was repeated independently at least three times, with each sample assessed in triplicate. For each experiment, at least two independent plasmid preparations were used. Relative lights units for each sample were always at least 100-fold above background. In addition, in some experiments, 5 ng of *in vitro*-transcribed RNA encoding Renilla luciferase was used as an additional internal control.

### Confocal microscopy.

Experiments were performed using CPT-Tert or A549 cells cultured in two-well glass chamber slides (Lab-Tek; Nalge Nunc International). The cells were either transfected with the appropriate plasmids or infected with the indicated viruses (MOI = 4) for 16 to 22 h. The cells were washed with phosphate-buffered saline (PBS) and fixed with 5% formaldehyde for 15 min. The fixed cells were then processed as described previously (62) and incubated with the appropriate antisera. Secondary antibodies were conjugated with either Alexa Fluor 488 or Alexa Fluor 594 (Invitrogen, Molecular Probes). Slides were mounted using Vectashield mounting medium with DAPI (4′,6-diamidino-2-phenylindole) (Vector Laboratories). The slides were analyzed and images were collected using a Leica TCS SP2 confocal microscope.

### Nucleotide sequence accession number.

RNA-seq data have been deposited in the European Nucleotide Archive (ENA) and can be accessed through the study accession number PRJEB13366.

## RESULTS

### BTV8ΔNS4 is attenuated in experimentally infected sheep.

**FIG 1:**
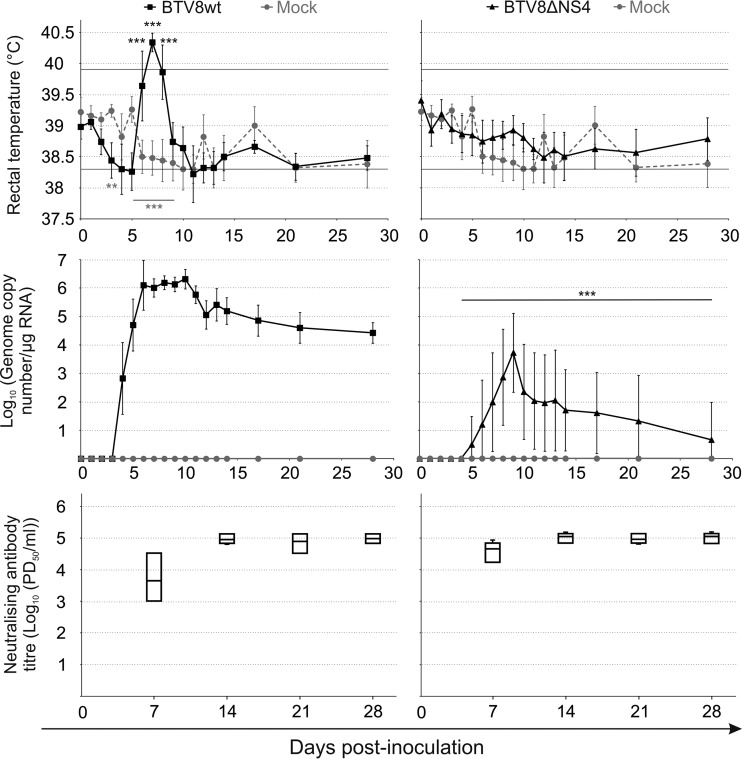
Experimental infection of sheep with BTV8wt and BTV8ΔNS4. Three groups of sheep (*n* = 5 per group) were mock infected or infected with either BTV8wt or BTV8ΔNS4. (Top) Body temperatures (average per group) of experimentally infected sheep. The rectal temperature of sheep is normally between 38.3 and 39.9°C (horizontal lines). (Middle) BTV RNA in blood samples of experimentally infected sheep. Viral RNA was detected by qRT-PCR targeting Seg-5, and the values are expressed as log_10_ copy number per microgram of total RNA. (Bottom) Box-and-whisker plot representing neutralizing antibodies in experimentally infected sheep at 7, 14, 21, and 28 days postinfection as described in Materials and Methods. PD_50_, 50% protective dose. **, *P* < 0.01; ***, *P* < 0.001 (two-way analysis of variance [ANOVA]; Bonferroni posttests) (gray stars, mock infected versus BTV8wt infected; black stars, BTV8wt infected versus BTV8ΔNS4 infected). The error bars represent standard deviations.

In a previous study, we showed that a BTV8 NS4 deletion mutant (BTV8ΔNS4) is as virulent as BTV8wt in experimental mouse models of infection (8). Here, we wanted to determine whether NS4 influenced virulence in sheep, the natural host of BTV infection. Therefore, we experimentally infected sheep with BTV8wt or BTV8ΔNS4. Sheep infected with BTV8wt showed an elevation of body temperature from day 6 to day 8 p.i. (Fig. 1, top). BTV RNA was detectable at day 4 p.i. and reached a plateau from days 6 to 10 p.i., followed by a slow decrease. Viremia remained detectable until the end of the experiment. In contrast, animals infected by BTV8ΔNS4 did not develop pyrexia and displayed a delayed onset and lower levels of viremia. BTV RNA was below the detection level in 4 of the 5 inoculated sheep at 4 weeks p.i., when the experiment was stopped (Fig. 1, middle). Between days 4 and 28 p.i., the average levels of BTV RNA were between 10^2^- and 10^5^-fold higher in sheep infected with BTV8wt than in those infected with BTV8ΔNS4. As expected, all BTV-infected animals developed neutralizing antibodies from day 7 p.i. (Fig. 1, bottom). Altogether, these data show that BTV8ΔNS4 is attenuated in sheep and suggest that NS4 is a virulence factor *in vivo*.

### Replication kinetics of BTV8wt and BTV8ΔNS4 in primary ovine endothelial cells and human A549 cells.

**FIG 2:**
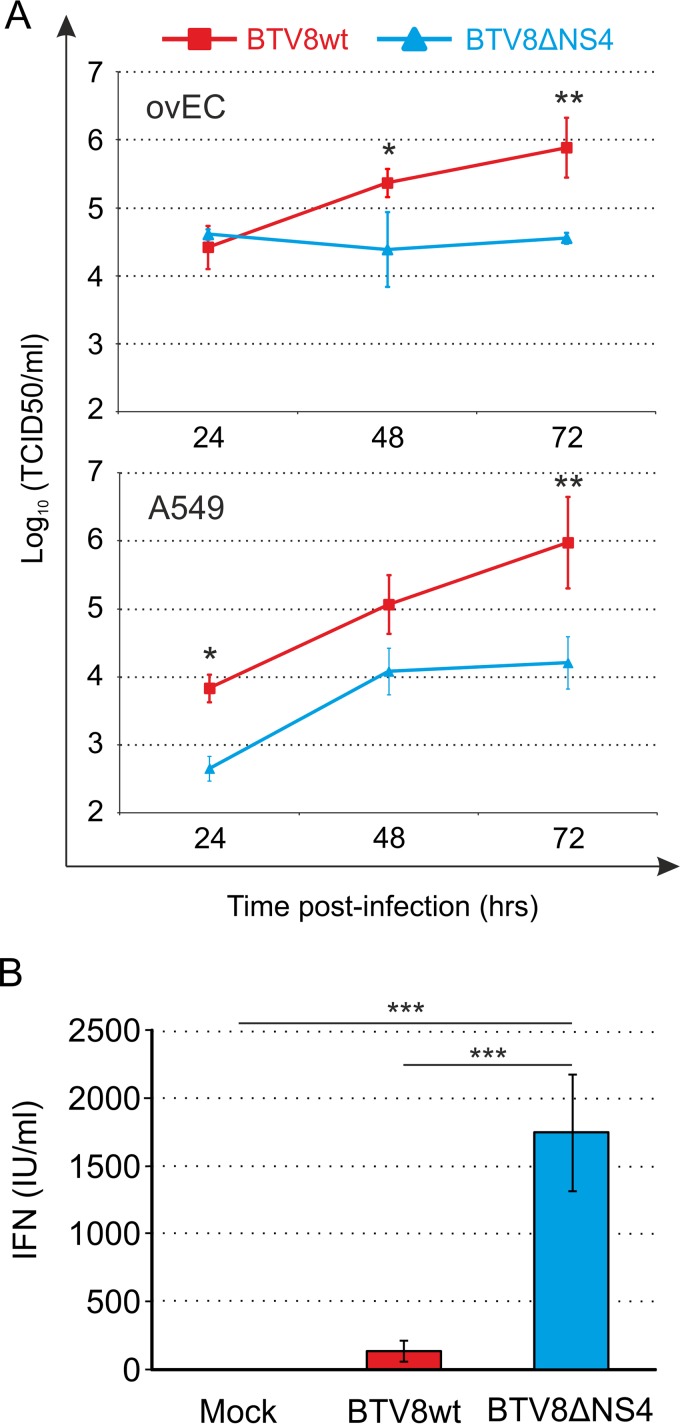
NS4 modulates IFN synthesis in infected cells. (A) *In vitro* replication kinetics of BTV8wt and BTV8ΔNS4 in primary ovEC and human A549 cells. The cells were infected with BTV8wt (red) and BTV8ΔNS4 (blue) at an MOI of 0.01, and the supernatants were titrated at the indicated time points as described in Materials and Methods. Experiments were performed independently three times. Two-way ANOVA, *P* < 0.01. *, *P* < 0.05; **, *P* < 0.01 (Bonferroni posttests). (B) Upregulation of IFN-β synthesis in BTV8-infected cells (IFN protection assay). Primary ovEC were mock infected or infected with the indicated viruses (MOI = 4). Supernatants were then collected at 16 h p.i. and inactivated by UV treatment, and the amount of IFN released in the supernatants was determined in biological assays as described in Materials and Methods. The error bars represent standard deviations. One-way ANOVA, *P* = 0.002. ***, *P* < 0.001 (Tukey's multiple-comparison test).

We showed previously that BTV8ΔNS4 replicates as efficiently as BTV8wt in immortalized cell lines, such as hamster BSR and sheep CPT-Tert cells (8), but these cell lines do not possess an intact IFN response to viral infections (44, 45). Hence, we compared the replication kinetics of BTV8wt and BTV8ΔNS4 in primary ovEC. BTV8wt reached titers approximately 20-fold higher than those of BTV8ΔNS4 at 72 h p.i. (Fig. 2A). Similarly, BTV8wt reached titers nearly 60-fold higher than those of BTV8ΔNS4 in the IFN-competent human cell line A549 (Fig. 2A). These data demonstrate that the presence of NS4 confers a replication advantage on BTV8 in cells that are capable of mounting an antiviral response.

### NS4 inhibits IFN release, but not virus sensing, in infected cells.

Given the data obtained as described above and the previously published observation that NS4 confers a replication advantage on BTV in cells pretreated with IFN (8), we next sought to determine whether NS4 impacted the level of IFN released into the supernatant of infected cells. Primary ovEC were infected at an MOI of 4 with either BTV8wt or BTV8ΔNS4. At 16 h p.i., the level of IFN in the cell culture medium was measured using an IFN protection assay. BTV8ΔNS4-infected cells had, on average, 13 times more IFN (*P* < 0.001) in their supernatants than BTV8wt-infected cells (Fig. 2B).

**FIG 3:**
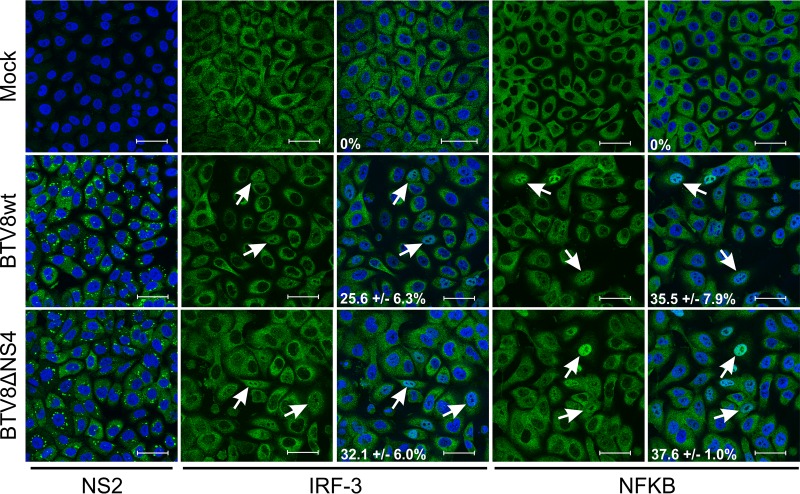
IRF-3 and NF-κB nuclear translocation in BTV-infected cells. A549 cells were infected at an MOI of 4 for 16 h and fixed before being processed for immunofluorescence using antibodies against NS2, IRF-3, and NF-κB with an Alexa Fluor 488 secondary antibody (green). Nuclei were stained with DAPI (blue). Average values (±standard deviations) corresponding to the percentage of translocation are indicated for each experimental condition. Scale bars, 47.62 μm.

These results could potentially be explained by NS4 affecting either the sensing of viral infection or transcription and/or the synthesis/secretion of IFN into the supernatant. The first step in IFN-β expression is the recognition of pathogen-associated molecular patterns (PAMPs) by various host pattern recognition receptors, which results in the translocation of the transcription factors IRF-3 and NF-κB into the nucleus (37). In order to determine whether NS4 impacted supernatant levels of IFN by interfering with this process, we infected A549 cells at an MOI of 4 for 16 h and assessed the nuclear translocation of IRF-3 and NF-κB by confocal microscopy. The cellular localization of IRF-3 and NF-κB in mock-infected cells was exclusively cytoplasmic (Fig. 3), while stimulation with tumor necrosis factor alfa (TNF-α) used as a positive control resulted in nuclear translocation of NF-κB in essentially 100% of the cells (not shown). In contrast, between 25 and 35% of cells infected by BTV8wt showed translocation of IRF-3 and NF-κB into the nucleus. BTV8ΔNS4 was found to induce levels of translocation similar to those of BTV8wt, suggesting that NS4 does not prevent either PAMP recognition by the host cells or translocation of IRF-3 and NF-κB. NS2 immunolabeling confirmed similar levels of infection in cells infected with BTV8wt or BTV8ΔNS4 (Fig. 3).

### NS4 downregulates IFN-β and ISG mRNA levels.

**FIG 4:**
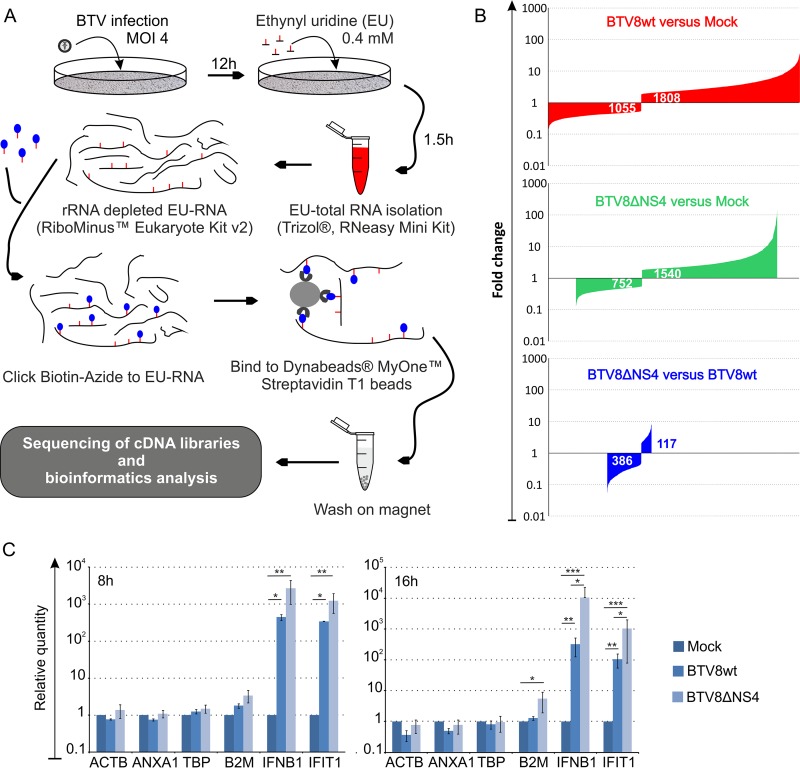
RNA-seq of BTV8wt-, BTV8ΔNS4-, and mock-infected cells. (A) Schematic representation of RNA-sequencing workflow. A549 cells were mock infected or infected with BTV8wt or BTV8ΔNS4 at an MOI of 4. At 12 h p.i., nascent RNA was metabolically labeled with an analogue of uridine (EU) at 0.4 mM for 90 min. Total RNA was extracted and enriched by selectively depleting rRNA transcripts. The EU-labeled RNA was chemically linked to azide-modified biotin and then captured on streptavidin magnetic beads. Biotin-labeled RNA attached to the magnetic beads was used directly to construct a library and subsequently sequenced using the Ion Proton sequencer. Sequence reads were processed as described in Materials and Methods. (B) Plots representing DE genes according to their fold change values. Log_2_-fold changes of >1 were regarded as upregulated (*q* value [false-discovery rate] < 0.05), whereas changes of <1 were regarded as statistically downregulated (*q* value < 0.05). (C) Validation of RNA-seq by qRT-PCR. mRNA levels of ACTB, ANXA1, TBP, B2M, IFN-β (IFNB1), and IFIT1 were measured by qRT-PCR at 8 and 16 h p.i. as described in Materials and Methods. *, *P* < 0.05; **, *P* < 0.01; ***, *P* < 0.001 (one-way ANOVA; Tukey's multiple-comparison test). The error bars represent standard deviations.

**FIG 5:**
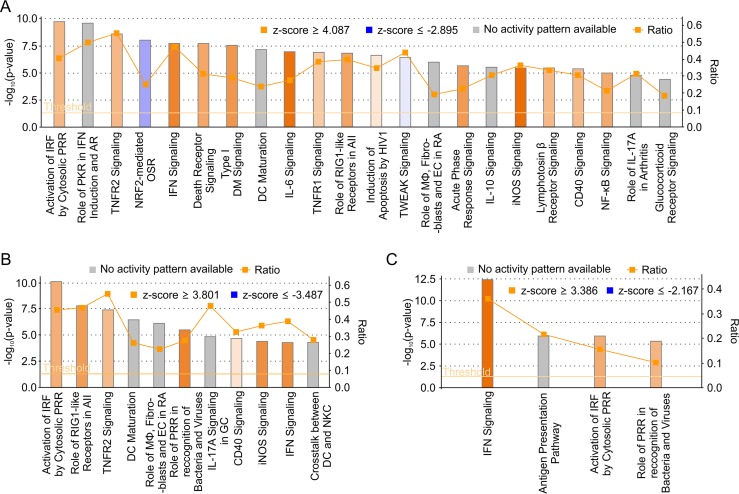
Cellular pathways of BTV8wt- and BTV8ΔNS4-infected cells. (A to C) Canonical cellular pathways were analyzed using Ingenuity Pathway Analysis. Only canonical pathways with a *P* value of <6.3 × 10^5^ are shown. The ratios correspond to the number of genes found DE in a given pathway divided by the total number of genes contained in that pathway. A specific pathway is colored in orange or blue depending on whether it has been predicted to be activated (positive Z-scores) or inhibited (negative Z-scores), respectively. The gray bars represent pathways where no prediction can currently be made. PRR, pattern recognition receptors; AII, antiviral innate immunity; DC, dendritic dell; MΦ, macrophages; EC, endothelial cells; RA, rheumatoid arthritis; GC, gastric cells; NKC, natural killer cells; AR, antiviral response; OSR, oxidative-stress response; DM, diabetes mellitus. (A) BTV8ΔNS4- versus mock-infected cells. (B) BTV8wt- versus mock-infected cells. (C) BTV8ΔNS4- versus BTV8wt-infected cells.

Next, we carried out RNA-seq analysis of IFN-competent A549 cells infected with either BTV8wt or BTV8ΔNS4 in order to further characterize the activity of NS4. Mock-infected A549 cells were used as negative controls. Libraries were prepared from nascent RNA metabolically labeled with a uracil analogue for 90 min at 12 h p.i. (Fig. 4A) and sequenced on an Ion Proton sequencer (Life Technologies, Thermo Fisher). On average, 64.58 million reads per sample were generated (Phred quality > 20). We found 2,863 differentially expressed genes in cells infected by BTV8wt compared to mock-infected cells, of which 1,055 were downregulated and 1,808 were upregulated (Fig. 4B). In comparison, fewer genes (*n* = 2,292) were found to be differentially expressed in cells infected by BTV8ΔNS4 than in mock-infected cells, with 752 of them being downregulated and 1,540 upregulated. The entire list of differentially expressed genes in BTV8wt and BTV8ΔNS4-infected cells is presented in Tables S1 to S3 in the supplemental material. The CH25H gene (encoding cholesterol 25-hydroxylase; a known interferon-stimulated gene [ISG]) was found to be the most upregulated gene in BTV8ΔNS4-infected cells. A total of 117 genes were upregulated in BTV8ΔNS4-infected cells compared to BTV8wt-infected cells (see Table S3 in the supplemental material). Of these 117 genes, 102 were either IFN genes or ISGs, according to the Interferome database (63). IFN-β, IFN-λ1, IFN-λ2, and IFN-λ3 genes were among the top 6 upregulated genes in BTV8ΔNS4-infected cells (87- to 136-fold more than in mock-infected cells) (Table 1). Interestingly, more genes were strongly upregulated (fold change > 32) in BTV8ΔNS4 (*n* = 33) than in BTV8wt-infected cells (*n* = 7). These genes included MX1 and -2; interferon-induced protein with tetratricopeptide repeats 1 (IFIT1), -2, and -3; and OASL genes and other well-characterized ISGs. However, all 33 of the genes highly upregulated in BTV8ΔNS4-infected cells were also upregulated (albeit at a lower level) in BTV8wt-infected cells (Table 1). The IFN-β gene, for example, was also upregulated (17-fold) in BTV8wt-infected cells. No major differences in the levels of expression in BTV8wt- and BTV8ΔNS4-infected cells were observed for the genes with the highest levels of downregulation (Table 2). We validated the RNA-seq analysis by qRT-PCR on selected genes found to be equally or differentially expressed in mock-infected and infected cells. Relative mRNA levels of IFNB1, IFIT1, beta-2 microglobulin (B2M), β-actin (ACTB), annexin A1 (ANXA1), and TATA-binding protein (TBP) determined by RT-qPCR reflected the patterns of expression observed in the RNA-seq analyses (Fig. 4C). The Ingenuity Pathway Analysis software (Qiagen) was used to compare the representation of canonical pathways between infected and uninfected cells (Fig. 5A and B) and cells infected with either BTV-8wt or BTV8ΔNS4 (Fig. 5C). Most of the pathways were involved in the cellular immune response, cytokine signaling, the inflammatory response, apoptosis, and pathogen-related signaling. Consistent with the RNA-seq data set, pathways relating to the innate immune response were particularly evident when comparing BTV8ΔNS4 with BTV8wt (Fig. 5C).

**TABLE 1:** Top 33 upregulated genes in BTV-infected compared to mock-infected A549 cells

Gene	BTV8ΔNS4 vs. mock infected		BTV8wt vs. mock infected	
	Rank^*a*^	Fold change	Rank	Fold change
CH25H	U1	142.3	U18	23.6
IFNB1	U2	136.4	U41	17.5
IFNL1	U3	105.6	U11	28.8
IFNL2	U4	91.0	U68	14.1
MX1	U5	88.6	U38	17.8
IFNL3	U6	87.7	U98	12.0
OASL	U7	87.6	U13	28.3
IFIT2	U8	84.7	U14	25.9
RSAD2	U9	78.7	U50	16.3
IFIT1	U10	75.9	U36	18.2
CXCL11	U11	71.6	U24	21.5
IFIT3	U12	71.0	U40	17.6
CMPK2	U13	64.1	U112	11.2
IFI44	U14	63.4	U25	21.4
MX2	U15	61.8	U76	13.6
CCR4	U16	56.3	U17	24.0
IFI27	U17	49.7	U170	8.9
DDX58	U18	48.4	U115	11.0
OAS2	U19	46.1	U5	33.6
IFIH1	U20	45.5	U22	21.9
CA1	U21	45.0	U9	30.9
ISG15	U22	43.7	U78	13.4
FOSB	U23	43.6	U3	35.0
EGR2	U24	42.5	U8	31.8
FOS	U25	39.9	U15	25.3
IL-8	U26	39.3	U29	19.8
KLRC2	U27	36.8	U10	29.0
EGR1	U28	36.1	U6	32.9
SLC1A3	U29	35.9	U46	16.7
EGR4	U30	35.7	U59	14.9
BATF2	U31	33.5	U548	4.3
HERC5	U32	32.4	U69	14.0
IFI6	U33	32.3	U303	6.2

a Ranks of upregulated genes. The number (U1, U2, etc.) refers to the rank of the specified gene. For example, the CH25H gene is the gene found to be the most upregulated in BTV8ΔNS4-infected cells compared to mock-infected cells.

**TABLE 2:** Top 20 downregulated genes in BTV-infected compared to mock-infected A549 cells

Gene	BTV8ΔNS4 vs. mock infected		BTV8wt vs. mock infected	
	Rank^*a*^	Fold change	Rank	Fold change
CLDND2	D1	0.13	D157	0.30
CPN1	D2	0.13	D24	0.20
DHRS4L1	D3	0.14	D425	0.38
EMID1	D4	0.14	NA^*b*^	1.0
LOC100506229	D5	0.16	D3	0.15
HMOX1	D6	0.16	D4	0.15
KRT82	D7	0.16	D73	0.26
SHH	D8	0.17	D5	0.15
NUPR1	D9	0.18	D11	0.18
KRT4	D10	0.18	D142	0.30
SFRP4	D11	0.19	D9	0.17
NRTN	D12	0.19	NA	1.0
EMILIN1	D13	0.20	D18	0.20
LOC113230	D14	0.20	NA	1.0
LINC00086	D15	0.20	D329	0.36
OSGIN1	D16	0.21	D10	0.18
KLHDC7A	D17	0.21	D1	0.12
ATOH8	D18	0.22	D2	0.14
MIR210HG	D19	0.22	D106	0.28
KRTAP4-1	D20	0.22	D27	0.21

a Ranks of downregulated genes. The numbers D1, D2, etc. refer to the rank of the specified gene (the most downregulated gene under a given condition is ranked first).

b NA, not applicable, as the genes were not found to be differentially expressed in the samples considered.

The availability of the transcriptome of cells infected by BTV8wt and BTV8ΔNS4 also provided additional information regarding the possible influence of NS4 on mRNA maturation. Comparable proportions of reads containing intron sequences were found in mock-infected cells and in cells infected with BTV8wt or BTV8ΔNS4 (data not shown). In addition, we also quantified how many reads finished with 8 or more adenines, assuming these reads to be representative of polyadenylated mRNAs. Comparable percentages of poly(A) reads were obtained under the three conditions tested, suggesting that NS4 was not associated with a global defect in mRNA polyadenylation (data not shown).

### NS4 modulates the activities of a wide range of promoters.

**FIG 6:**
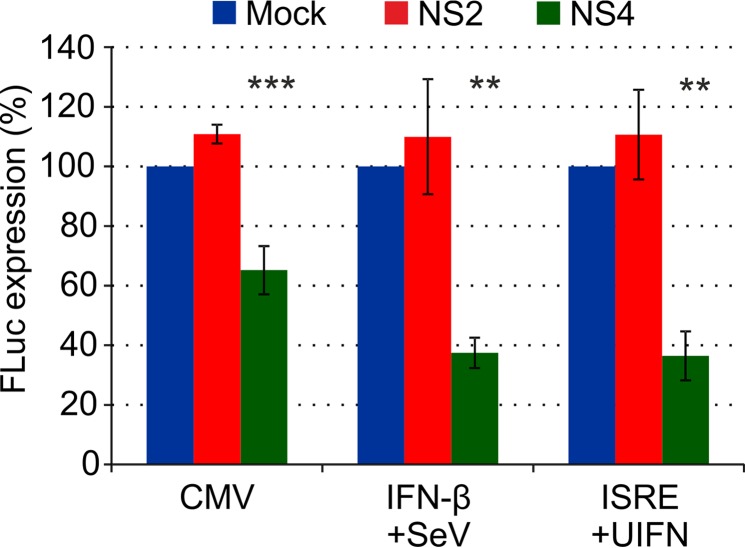
Effects of NS4 on expression driven by various promoters. CPT-Tert cells were cotransfected with 100 ng of a DNA plasmid expressing either BTV10 NS2 (GenBank accession number NC006007) or BTV8 NS4 and a DNA plasmid expressing FLuc under the control of either the CMV (50 ng), IFN-β (50 ng), or ISRE (400 ng) promoter. After transfection (4 h), cells transfected with the IFN-β promoter were stimulated with Sendai virus, while cells transfected with ISRE-containing promoter were stimulated with universal IFN. FLuc activity was assessed 22 h posttransfection in a luminometer. Five nanograms of capped and polyadenylated RNA made *in vitro* were also cotransfected as a control for transfection efficiency. One-way ANOVA, *P* = 0.0002 (CMV), *P* = 0.0015 (IFN-β), and *P* = 0.0006 (ISRE). **, *P* < 0.01; ***, *P* < 0.001 (Dunnett's multiple-comparison test).

We next assessed the ability of BTV-8 NS4 to reduce the activities of basal promoters, such as the cytomegalovirus (CMV) immediate-early promoter and the promoters of genes involved in the host innate immune response (IFN-β and ISRE-containing promoters). 293T cells were cotransfected with a plasmid expressing either BTV NS4 or NS2 and an FLuc reporter plasmid driven by either the CMV promoter, the IFN-β promoter, or a promoter containing ISRE elements (Fig. 5). At 4 h posttransfection, cells transfected with the IFN-β promoter were stimulated with Sendai virus, while cells transfected with the ISRE-containing promoter were stimulated with universal IFN (200 U/ml). NS4, unlike NS2, was able to reduce by between 40 and 60% the activities of CMV, IFN-β, and ISRE promoters (Fig. 6).

**FIG 7:**
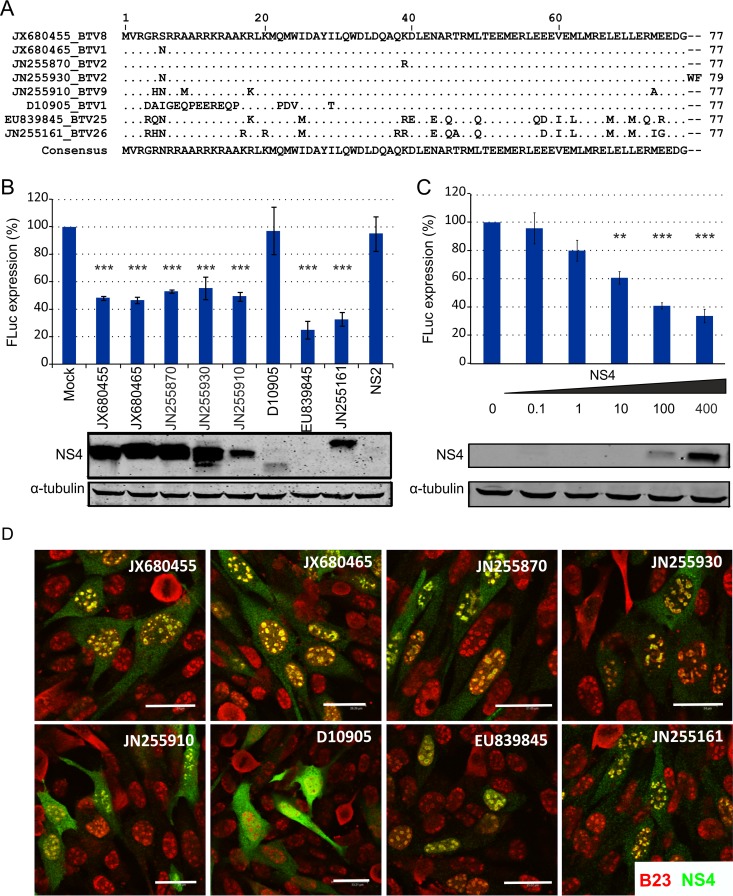
NS4 proteins from various BTV strains display inhibitory activity on gene expression. (A) Multiple-sequence alignment of representative NS4 sequences. Identical residues compared to BTV8 (GenBank accession number JX680455) are shown as dots. The accession numbers refer to BTV genome segment 9 containing the NS4 open reading frame. (B) CPT-Tert cells were cotransfected with a plasmid expressing the indicated BTV NS4 protein (named according to the accession number corresponding to BTV genome segment 9) and a plasmid expressing FLuc under the control of a CMV promoter. FLuc activity was assessed 22 h posttransfection and in parallel by Western blotting of the same cell lysates using anti-NS4 or anti-α-tubulin antibodies. One-way ANOVA, *P* < 0.0001. ***, *P* < 0.001 (Dunnett's multiple-comparison test). (C) CPT-Tert cells were cotransfected with increasing amounts of an expression plasmid for BTV8 NS4 and a plasmid expressing FLuc under the control of the CMV immediate-early promoter. FLuc activity was assessed 22 h posttransfection in a luminometer and in parallel by Western blotting of the same cell lysates using antibodies to NS4 or tubulin antibodies. One-way ANOVA, *P* < 0.0001. **, *P* < 0.01; ***, *P* < 0.001 (Dunnett's multiple-comparison test). (D) Confocal microscopy of CPT-Tert cells transfected with pCI-NS4 plasmids. At 22 h posttransfection, the cells were fixed and analyzed by immunofluorescence using antibodies against the nucleolar marker B23 (red) and NS4 (green), with the appropriate conjugated secondary antibodies as described in Materials and Methods. Scale bars, 33.21 μm. The error bars represent standard deviations.

To further investigate the ability of NS4 to block host gene expression, sheep cells were cotransfected with an expression plasmid for FLuc, under the control of the CMV immediate-early promoter, along with a variety of expression plasmids expressing BTV NS4 or an empty plasmid (also containing a CMV promoter) as a control (Fig. 7). NS4 is well conserved among the BTV serotypes/strains identified to date (8). The only exceptions were a strain of BTV-1 (GenBank accession number D10905, submitted in 1992) and the more divergent BTV-25 and BTV-26 strains (GenBank accession numbers EU839845 and JN255161), which showed only 77.9%, 76.6%, and 75.3% identity to BTV-8 NS4, respectively (Fig. 7A). All of the NS4 proteins tested were able to reduce FLuc expression, with the notable exception of NS4 from BTV-1 (D10905) (Fig. 7B). Interestingly, the NS4 protein of BTV-1 (D10905) displayed a mobility different from those of other NS4 proteins by SDS-PAGE that could be explained by the presence of considerable differences in the basic residues in the N terminus. In addition, using BTV-8 NS4, we showed that gene expression inhibition by NS4 was dose dependent (Fig. 7C).

In a previous study, we showed that NS4 localizes in the nucleoli of infected or transfected cells (8). Interestingly, BTV-1 (D10905) NS4 was the only variant that failed to localize to the nucleoli of sheep CPT-Tert cells (Fig. 7D), suggesting that nucleolar localization may be critical for the activity of this nonstructural protein.

### NS4 is not the only viral protein involved in host cell protein shutoff.

**FIG 8:**
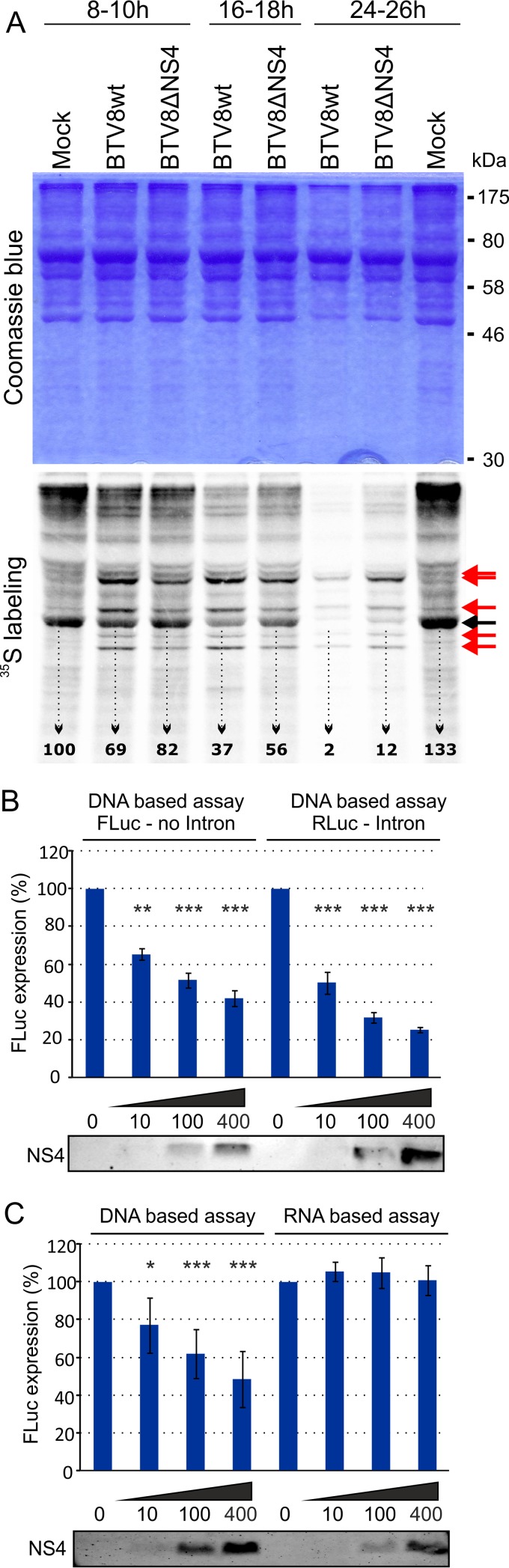
NS4 expression does not significantly affect host cellular protein shutoff, mRNA splicing, or translation. (A) Primary ovEC were mock infected or infected with BTV8wt and BTV8ΔNS4, and nascent proteins were metabolically labeled with [^35^S]methionine/cysteine for 2 h at the time points indicated. Cell extracts were fractionated by SDS-PAGE, and the gels were stained with Coomassie blue. The dried gels were analyzed by phosphorimaging. The black arrow indicates cellular actin. The signal intensity was quantified using ImageQuant software. The red arrows indicate BTV proteins. (B) CPT-Tert cells were cotransfected with variable amounts of plasmids expressing BTV8 NS4 protein and either a plasmid expressing FLuc under the control of a CMV promoter or a plasmid expressing RLuc downstream of an intron and the CMV promoter. FLuc or RLuc expression was assessed 22 h posttransfection. Cell lysates were analyzed by Western blotting using antibodies to NS4. One-way ANOVA, *P* < 0.0001. **, *P* < 0.01; ***, *P* < 0.001 (Dunnett's multiple-comparison test). (C) CPT-Tert cells were cotransfected with variable amounts of a DNA plasmid expressing BTV8 NS4 and either a plasmid expressing FLuc under the control of a CMV promoter or capped and polyadenylated RNA made *in vitro*. FLuc activity was assessed 22 h posttransfection. Cell lysates were also analyzed by Western blotting using antibodies to NS4. One-way ANOVA, *P* < 0.0001. *, *P* < 0.05; ***, *P* < 0.001 (Dunnett's multiple-comparison test). The error bars represent standard deviations.

It is well established that BTV induces protein synthesis shutoff in infected cells (64–66). Conceivably, a reduced level of IFN (both mRNA and proteins) observed in cells infected with BTV8wt could reflect the general virus-induced shutoff of protein synthesis. In order to test this hypothesis, we metabolically radiolabeled nascent proteins in ovEC mock infected or infected with either BTV8wt or BTV8ΔNS4. As expected, we observed decreased levels of ^35^S-labeled methionine/cysteine proteins in BTV8wt-infected cells compared to mock-infected cells (particularly evident at 18 and 26 h p.i.), confirming previously published data (Fig. 8) (65, 66). Protein synthesis shutoff was also evident in BTV8ΔNS4-infected cells, although at somewhat reduced levels compared to BTV8wt-infected cells. In order to quantify the reduction in protein synthesis during viral infection we used phosphorimaging and measured the signal intensity of a prominent band present in all samples (Fig. 8A, black arrow). The signal intensity of actin in BTV8wt-infected cells relative to mock-infected cells (taken as 100%) decreased progressively to 69% at 10 h p.i., 37% at 18 h p.i., and 2% at 24 h p.i., by which point cytopathic effect was apparent. On the other hand, the signal intensity of actin decreased to 82%, 56%, and 12%. Hence, these data suggest that host protein shutoff induced by BTV occurs largely independently of NS4, although the protein may contribute to the phenomenon.

### BTV-8 NS4 does not influence mRNA splicing or translation.

We also assessed the impact of NS4 on RNA transcript splicing. CPT-Tert cells were transfected with the pRL-CMV vector, which is driven by the CMV immediate-early promoter and contains the RLuc gene downstream of an intron. In this assay, NS4 retained the ability to inhibit the expression of the reporter gene in a dose-dependent manner (Fig. 8B), indicating that NS4 does not affect mRNA splicing and confirming the data obtained by RNA-seq as described above. Cells were also cotransfected with *in vitro*-transcribed RNA encoding FLuc and an expression plasmid for BTV8 NS4, in order to determine whether NS4-induced inhibition of protein expression could also occur at the translational level (Fig. 8C). Increasing levels of NS4 did not interfere with the level of FLuc activity driven by RNA, as opposed to the activity driven by plasmid DNA, used as a control, suggesting that the inhibition mediated by NS4 most likely occurs at the transcriptional and not at the translational level.

## DISCUSSION

In this study, we showed that NS4 is an IFN antagonist and a virulence factor for BTV. BTV NS4 deletion mutants replicate as efficiently as wild-type viruses in cell lines that lack a competent innate immune system and are lethal to IFNAR^−/−^ mice (8). In contrast, here, we demonstrated that NS4 facilitates BTV replication *in vitro* in IFN-competent cells and *in vivo* in sheep, the animal species most affected by bluetongue.

BTV induces a type 1 IFN response *in vivo* and *in vitro* (38–42). Our data strongly suggest that NS4 is required by the virus to help overcome the cellular innate antiviral responses. Reporter assays revealed that NS4 can inhibit transcription from a variety of mammalian and viral promoters while having little if any direct impact on mRNA editing or translation. However, NS4 does appear to influence the extent of the host IFN response, as supernatants of primary sheep endothelial cells infected with BTV8ΔNS4 contained more IFN than supernatants collected from BTV8wt-infected cells, a result that may explain the more efficient growth of BTVwt in IFN-competent cells. These results were supported by RNA-seq analyses of nascent RNA isolated from infected and mock-infected cells. Genes relating to the innate immune system were among the most upregulated genes in BTV8ΔNS4-infected cells, even if some of them were also found to be upregulated in BTV8wt-infected cells, suggesting that NS4 is not by itself sufficient to entirely inhibit the host IFN response. Most of the genes that were specifically upregulated in BTV8ΔNS4-infected cells compared to BTV8wt-infected cells were ISGs, including those encoding well-characterized antiviral factors.

Overall, the data obtained in this study indicate that NS4 exerts its effect upon the antiviral host response at a point upstream of RNA editing and translation. Interestingly, NS4 possesses features of a transcription factor of the bZip family, with a basic domain followed by a leucine zipper motif (67), and it has been suggested to have the ability to bind DNA, based on DNase protection assays (9). Our reporter assays, showing that NS4 inhibits transcription from a variety of promoters, including IFN-β and an ISRE-containing promoter, suggest that the protein may inhibit cellular transcription. In a previous study, we showed that NS4 displays nucleolar localization in infected cells (8). Thus, a direct interaction of NS4 with transcription factors and/or chromatin resulting in the downregulation of cellular transcription is a possible mechanism of action for the protein. In support of this hypothesis, we found that the NS4 protein of a BTV-1 strain that did not show nucleolar localization was not able to modulate gene expression in reporter assays. It is also possible that NS4 inhibits IFN-β activation before transcription. Nuclear translocation of IRF-3 and NF-κB appeared to occur at similar levels in both BTVwt- and BTVΔNS4-infected cells, suggesting that NS4 does not prevent PAMP recognition by host cells. However, it is possible that IRF-3 or NF-κB could be inhibited by NS4 in the nucleus. It is important to note that, in a previous study, we showed that NS4 confers a replication advantage on BTV in cells pretreated with type I IFN, highlighting its ability to counteract the innate immune response after IFN activation. Hence, it is less likely that NS4 blocks PAMP recognition and subsequent signaling. More studies will be necessary to fully dissect how NS4 counteracts the host innate immune system. Obviously, NS4, like other nonstructural proteins of different viruses, might have different functions and antagonize the IFN response via multiple mechanisms. For example, the NS1 protein of influenza A viruses inhibits IFN production by blocking IRF3 and NF-κB activation and mRNA maturation (68, 69).

It has been known for at least 3 decades that BTV induces host protein synthesis shutdown (22, 64, 65). Recent studies suggest that BTV NS1 favors viral RNA translation and therefore competes with cellular protein synthesis (22). A key difference between mammalian and BTV transcripts is the lack of a poly(A) tail. The NSP3 protein of rotaviruses (also members of the Reoviridae) acts in a similar fashion and outcompetes the poly(A) binding protein of cells, thus biasing translation of viral transcripts (70, 71). It remains possible that a similar mechanism may exist for BTV. Our data, obtained in metabolically labeled cells, confirm that BTV induces host protein synthesis shutdown and also establishes that this occurs largely independently of NS4. Hence, other BTV proteins might also play a similar role in the shutoff of host cell protein synthesis or function in different ways as IFN antagonists. For example, NS3 is believed to modulate IFN induction downstream of RIG-I and upstream of IKKε activation. Therefore, NS3 and NS4 may potentially act synergistically to counteract the innate immune system (28). In addition, it has also been shown that BTV inhibits the IFN signaling pathway by downregulating key components of the JAK/STAT pathway: JAK1 and TYK2 (43). The downregulation of JAK1/TYK2 may be the result of a specific interaction with a BTV protein and/or due to the general host protein synthesis shutdown induced by the virus. We did not find either of the two genes to be differentially expressed in BTV8wt- or BTV8ΔNS4-infected cells.

In this study, we showed that BTV8ΔNS4-infected sheep display lower levels of viremia, lasting for shorter periods, than the viremia observed in animals infected with BTV8wt. The levels and duration of viremia in infected animals are essential factors for successful transmission between the mammalian hosts and the intermediate arthropod vectors. As mentioned above, BTV infection can result in a lethal hemorrhagic fever in some animals, while in others, the virus induces a mild febrile illness or even a clinically unapparent infection. The data obtained in this study provide evidence that viral proteins modulating the host innate immune response play a significant role in viral pathogenesis. This study lays the foundations for a deeper understanding of the mechanisms by which BTV antagonizes the IFN system, in turn helping us to define the molecular determinants of BTV virulence.

## Supplementary Material

Supplemental material

## References

[1] Mellor PS, Baylis M, Mertens PP 2009 Bluetongue. Academic Press, London, United Kingdom.

[2] Mellor PS, Carpenter S, Harrup L, Baylis M, Mertens PP 2008 Bluetongue in Europe and the Mediterranean Basin: history of occurrence prior to 2006. Prev Vet Med 87:4–20. doi:10.1016/j.prevetmed.2008.06.002. 18619694

[3] Wilson AJ, Mellor PS 2009 Bluetongue in Europe: past, present and future. Philos Trans R Soc Lond B Biol Sci 364:2669–2681. doi:10.1098/rstb.2009.0091. 19687037 PMC2865089

[4] Mellor PS, Carpenter S, White DM 2009 Bluetongue virus in the insect vector, p 295–320. *In* Mellor P, Baylis M, Mertens P (ed), Bluetongue. Elsevier, Amsterdam, Netherlands.

[5] Price DA, Hardy WT 1954 Isolation of the bluetongue virus from Texas sheep: Culicoides shown to be a vector. J Am Vet Med Assoc 124:255–258. 13142963

[6] Gouet P, Diprose JM, Grimes JM, Malby R, Burroughs JN, Zientara S, Stuart DI, Mertens PP 1999 The highly ordered double-stranded RNA genome of bluetongue virus revealed by crystallography. Cell 97:481–490. doi:10.1016/S0092-8674(00)80758-8. 10338212

[7] Patel A, Roy P 2014 The molecular biology of Bluetongue virus replication. Virus Res 182:5–20. doi:10.1016/j.virusres.2013.12.017. 24370866 PMC7162684

[8] Ratinier M, Caporale M, Golder M, Franzoni G, Allan K, Nunes SF, Armezzani A, Bayoumy A, Rixon F, Shaw A, Palmarini M 2011 Identification and characterization of a novel non-structural protein of bluetongue virus. PLoS Pathog 7:e1002477. doi:10.1371/journal.ppat.1002477. 22241985 PMC3248566

[9] Belhouchet M, Mohd Jaafar F, Firth AE, Grimes JM, Mertens PP, Attoui H 2011 Detection of a fourth orbivirus non-structural protein. PLoS One 6:e25697. doi:10.1371/journal.pone.0025697. 22022432 PMC3192121

[10] Grimes JM, Jakana J, Ghosh M, Basak AK, Roy P, Chiu W, Stuart DI, Prasad BV 1997 An atomic model of the outer layer of the bluetongue virus core derived from X-ray crystallography and electron cryomicroscopy. Structure 5:885–893. doi:10.1016/S0969-2126(97)00243-8. 9261080

[11] Roy P 2008 Bluetongue virus: dissection of the polymerase complex. J Gen Virol 89:1789–1804. doi:10.1099/vir.0.2008/002089-0. 18632949 PMC2735681

[12] Roy P, Noad R 2006 Bluetongue virus assembly and morphogenesis. Curr Top Microbiol Immunol 309:87–116. 16909898 10.1007/3-540-30773-7_4

[13] Forzan M, Marsh M, Roy P 2007 Bluetongue virus entry into cells. J Virol 81:4819–4827. doi:10.1128/JVI.02284-06. 17267479 PMC1900141

[14] Hassan SS, Roy P 1999 Expression and functional characterization of bluetongue virus VP2 protein: role in cell entry. J Virol 73:9832–9842. 10559295 10.1128/jvi.73.12.9832-9842.1999 PMC113032

[15] Zhang X, Boyce M, Bhattacharya B, Schein S, Roy P, Zhou ZH 2010 Bluetongue virus coat protein VP2 contains sialic acid-binding domains, and VP5 resembles enveloped virus fusion proteins. Proc Natl Acad Sci U S A 107:6292–6297. doi:10.1073/pnas.0913403107. 20332209 PMC2852009

[16] Huismans H, Erasmus BJ 1981 Identification of the serotype-specific and group-specific antigens of bluetongue virus. Onderstepoort J Vet Res 48:51–58. 6273773

[17] Kahlon J, Sugiyama K, Roy P 1983 Molecular basis of bluetongue virus neutralization. J Virol 48:627–632. 6313962 10.1128/jvi.48.3.627-632.1983 PMC255393

[18] Shaw AE, Ratinier M, Nunes SF, Nomikou K, Caporale M, Golder M, Allan K, Hamers C, Hudelet P, Zientara S, Breard E, Mertens P, Palmarini M 2013 Reassortment between two serologically unrelated bluetongue virus strains is flexible and can involve any genome segment. J Virol 87:543–557. doi:10.1128/JVI.02266-12. 23097432 PMC3536370

[19] Zientara S, Sailleau C, Viarouge C, Hoper D, Beer M, Jenckel M, Hoffmann B, Romey A, Bakkali-Kassimi L, Fablet A, Vitour D, Breard E 2014 Novel bluetongue virus in goats, Corsica, France, 2014. Emerg Infect Dis 20:2123–2125. doi:10.3201/eid2012.140924. 25418049 PMC4257820

[20] Owens RJ, Limn C, Roy P 2004 Role of an arbovirus nonstructural protein in cellular pathogenesis and virus release. J Virol 78:6649–6656. doi:10.1128/JVI.78.12.6649-6656.2004. 15163755 PMC416502

[21] Monastyrskaya K, Gould EA, Roy P 1995 Characterization and modification of the carboxy-terminal sequences of bluetongue virus type 10 NS1 protein in relation to tubule formation and location of an antigenic epitope in the vicinity of the carboxy terminus of the protein. J Virol 69:2831–2841. 7535866 10.1128/jvi.69.5.2831-2841.1995 PMC188978

[22] Boyce M, Celma CC, Roy P 2012 Bluetongue virus non-structural protein 1 is a positive regulator of viral protein synthesis. Virol J 9:178. doi:10.1186/1743-422X-9-178. 22931514 PMC3479040

[23] Butan C, Tucker P 2010 Insights into the role of the non-structural protein 2 (NS2) in Bluetongue virus morphogenesis. Virus Res 151:109–117. doi:10.1016/j.virusres.2010.05.014. 20621672

[24] Kar AK, Bhattacharya B, Roy P 2007 Bluetongue virus RNA binding protein NS2 is a modulator of viral replication and assembly. BMC Mol Biol 8:4. doi:10.1186/1471-2199-8-4. 17241458 PMC1794256

[25] Lymperopoulos K, Noad R, Tosi S, Nethisinghe S, Brierley I, Roy P 2006 Specific binding of Bluetongue virus NS2 to different viral plus-strand RNAs. Virology 353:17–26. doi:10.1016/j.virol.2006.04.022. 16872657 PMC7116519

[26] Beaton AR, Rodriguez J, Reddy YK, Roy P 2002 The membrane trafficking protein calpactin forms a complex with bluetongue virus protein NS3 and mediates virus release. Proc Natl Acad Sci U S A 99:13154–13159. doi:10.1073/pnas.192432299. 12235365 PMC130602

[27] Celma CC, Roy P 2009 A viral nonstructural protein regulates bluetongue virus trafficking and release. J Virol 83:6806–6816. doi:10.1128/JVI.00263-09. 19369335 PMC2698550

[28] Chauveau E, Doceul V, Lara E, Breard E, Sailleau C, Vidalain PO, Meurs EF, Dabo S, Schwartz-Cornil I, Zientara S, Vitour D 2013 NS3 of bluetongue virus interferes with the induction of type I interferon. J Virol 87:8241–8246. doi:10.1128/JVI.00678-13. 23658442 PMC3700197

[29] Stewart M, Hardy A, Barry G, Pinto RM, Caporale M, Melzi E, Hughes J, Taggart A, Janowicz A, Varela M, Ratinier M, Palmarini M 2015 Characterization of a second open reading frame in genome segment 10 of bluetongue virus. J Gen Virol 96:3280–3293. doi:10.1099/jgv.0.000267. 26290332 PMC4806581

[30] Caporale M, Di Gialleonorado L, Janowicz A, Wilkie G, Shaw A, Savini G, Van Rijn PA, Mertens P, Di Ventura M, Palmarini M 2014 Virus and host factors affecting the clinical outcome of bluetongue virus infection. J Virol 88:10399–10411. doi:10.1128/JVI.01641-14. 24991012 PMC4178883

[31] Janowicz A, Caporale M, Shaw A, Gulletta S, Di Gialleonardo L, Ratinier M, Palmarini M 2015 Multiple genome segments determine the virulence of bluetongue virus serotype 8. J Virol 89:5238–5249. doi:10.1128/JVI.00395-15. 25822026 PMC4442542

[32] Caporale M, Wash R, Pini A, Savini G, Franchi P, Golder M, Patterson-Kane J, Mertens P, Di Gialleonardo L, Armillotta G, Lelli R, Kellam P, Palmarini M 2011 Determinants of bluetongue virus virulence in murine models of disease. J Virol 85:11479–11489. doi:10.1128/JVI.05226-11. 21865388 PMC3194974

[33] Maclachlan NJ, Drew CP, Darpel KE, Worwa G 2009 The pathology and pathogenesis of bluetongue. J Comp Pathol 141:1–16. doi:10.1016/j.jcpa.2009.04.003. 19476953

[34] Brenner J, Oura C, Asis I, Maan S, Elad D, Maan N, Friedgut O, Nomikou K, Rotenberg D, Bumbarov V, Mertens P, Yadin H, Batten C 2010 Multiple serotypes of bluetongue virus in sheep and cattle, Israel. Emerg Infect Dis 16:2003–2004. doi:10.3201/eid1612.100239. 21122245 PMC3294591

[35] Anderson GA, Stott JL, Gershwin LJ, Osburn BI 1985 Subclinical and clinical bluetongue disease in cattle: clinical, pathological and pathogenic considerations. Prog Clin Biol Res 178:103–107. 2989843

[36] Yan N, Chen ZJ 2012 Intrinsic antiviral immunity. Nat Immunol 13:214–222. doi:10.1038/ni.2229. 22344284 PMC3549670

[37] Randall RE, Goodbourn S 2008 Interferons and viruses: an interplay between induction, signalling, antiviral responses and virus countermeasures. J Gen Virol 89:1–47. doi:10.1099/vir.0.83391-0. 18089727

[38] Chauveau E, Doceul V, Lara E, Adam M, Breard E, Sailleau C, Viarouge C, Desprat A, Meyer G, Schwartz-Cornil I, Ruscanu S, Charley B, Zientara S, Vitour D 2012 Sensing and control of bluetongue virus infection in epithelial cells via RIG-I and MDA5 helicases. J Virol 86:11789–11799. doi:10.1128/JVI.00430-12. 22915805 PMC3486277

[39] Huismans H 1969 Bluetongue virus-induced interferon synthesis. Onderstepoort J Vet Res 36:181–185. 4340875

[40] Jameson P, Schoenherr CK, Grossberg SE 1978 Bluetongue virus, an exceptionally potent interferon inducer in mice. Infect Immun 20:321–323. 208975 10.1128/iai.20.1.321-323.1978 PMC421856

[41] MacLachlan NJ, Thompson J 1985 Bluetongue virus-induced interferon in cattle. Am J Vet Res 46:1238–1241. 2411172

[42] Ruscanu S, Pascale F, Bourge M, Hemati B, Elhmouzi-Younes J, Urien C, Bonneau M, Takamatsu H, Hope J, Mertens P, Meyer G, Stewart M, Roy P, Meurs EF, Dabo S, Zientara S, Breard E, Sailleau C, Chauveau E, Vitour D, Charley B, Schwartz-Cornil I 2012 The double-stranded RNA bluetongue virus induces type I interferon in plasmacytoid dendritic cells via a MYD88-dependent TLR7/8-independent signaling pathway. J Virol 86:5817–5828. doi:10.1128/JVI.06716-11. 22438548 PMC3347300

[43] Doceul V, Chauveau E, Lara E, Breard E, Sailleau C, Zientara S, Vitour D 2014 Dual modulation of type I interferon response by bluetongue virus. J Virol 88:10792–10802. doi:10.1128/JVI.01235-14. 25008919 PMC4178850

[44] Buchholz UJ, Finke S, Conzelmann KK 1999 Generation of bovine respiratory syncytial virus (BRSV) from cDNA: BRSV NS2 is not essential for virus replication in tissue culture, and the human RSV leader region acts as a functional BRSV genome promoter. J Virol 73:251–259. 9847328 10.1128/jvi.73.1.251-259.1999 PMC103829

[45] Arnaud F, Black SG, Murphy L, Griffiths DJ, Neil SJ, Spencer TE, Palmarini M 2010 Interplay between ovine bone marrow stromal cell antigen 2/tetherin and endogenous retroviruses. J Virol 84:4415–4425. doi:10.1128/JVI.00029-10. 20181686 PMC2863748

[46] Varela M, Schnettler E, Caporale M, Murgia C, Barry G, McFarlane M, McGregor E, Piras IM, Shaw A, Lamm C, Janowicz A, Beer M, Glass M, Herder V, Hahn K, Baumgartner W, Kohl A, Palmarini M 2013 Schmallenberg virus pathogenesis, tropism and interaction with the innate immune system of the host. PLoS Pathog 9:e1003133. doi:10.1371/journal.ppat.1003133. 23326235 PMC3542112

[47] Boyce M, Celma CC, Roy P 2008 Development of reverse genetics systems for bluetongue virus: recovery of infectious virus from synthetic RNA transcripts. J Virol 82:8339–8348. doi:10.1128/JVI.00808-08. 18562540 PMC2519640

[48] Dulbecco R, Vogt M 1953 Some problems of animal virology as studied by the plaque technique. Cold Spring Harbor Symp Quant Biol 18:273–279. doi:10.1101/SQB.1953.018.01.039. 13168995

[49] Killip MJ, Young DF, Precious BL, Goodbourn S, Randall RE 2012 Activation of the beta interferon promoter by paramyxoviruses in the absence of virus protein synthesis. J Gen Virol 93:299–307. doi:10.1099/vir.0.037531-0. 22049094 PMC3352343

[50] Killip MJ, Young DF, Ross CS, Chen S, Goodbourn S, Randall RE 2011 Failure to activate the IFN-beta promoter by a paramyxovirus lacking an interferon antagonist. Virology 415:39–46. doi:10.1016/j.virol.2011.03.027. 21511322 PMC3107429

[51] Reed LJ, Muench HA 1938 A simple method for estimating fifty percent endpoints. Am J Hygiene 27:493–497.

[52] Lelli R, Di Ventura M, Mercante MT, Tittarelli M, Mangana-Vougiouka O, Nomikou K, Conte A, Di Emidio B, Portanti O, Giovannucci G, Bonfini B, Zaghini M, Caporale V 2004 Bluetongue laboratory diagnosis: a ring test to evaluate serological results using a competitive ELISA kit. Vet Ital 40:577–580. 20422590

[53] Polci A, Camma C, Serini S, Di Gialleonardo L, Monaco F, Savini G 2007 Real-time polymerase chain reaction to detect bluetongue virus in blood samples. Vet Ital 43:77–88. 20411502

[54] Palmarini M, Hallwirth C, York D, Murgia C, de Oliveira T, Spencer T, Fan H 2000 Molecular cloning and functional analysis of three type D endogenous retroviruses of sheep reveal a different cell tropism from that of the highly related exogenous jaagsiekte sheep retrovirus. J Virol 74:8065–8076. doi:10.1128/JVI.74.17.8065-8076.2000. 10933716 PMC112339

[55] Hale BG, Steel J, Medina RA, Manicassamy B, Ye J, Hickman D, Hai R, Schmolke M, Lowen AC, Perez DR, Garcia-Sastre A 2010 Inefficient control of host gene expression by the 2009 pandemic H1N1 influenza A virus NS1 protein. J Virol 84:6909–6922. doi:10.1128/JVI.00081-10. 20444891 PMC2898253

[56] Tanaka N, Kawakami T, Taniguchi T 1993 Recognition DNA sequences of interferon regulatory factor 1 (IRF-1) and IRF-2, regulators of cell growth and the interferon system. Mol Cell Biol 13:4531–4538. doi:10.1128/MCB.13.8.4531. 7687740 PMC360068

[57] Trapnell C, Roberts A, Goff L, Pertea G, Kim D, Kelley DR, Pimentel H, Salzberg SL, Rinn JL, Pachter L 2012 Differential gene and transcript expression analysis of RNA-seq experiments with TopHat and Cufflinks. Nat Protoc 7:562–578. doi:10.1038/nprot.2012.016. 22383036 PMC3334321

[58] Babraham Bioinformatics. FastQC, a quality control tool for high throughput sequence data. http://www.bioinformatics.babraham.ac.uk/projects/fastqc/.

[59] Trapnell C, Hendrickson DG, Sauvageau M, Goff L, Rinn JL, Pachter L 2013 Differential analysis of gene regulation at transcript resolution with RNA-seq. Nat Biotechnol 31:46–53. doi:10.1038/nbt.2450. 23222703 PMC3869392

[60] Langmead B, Salzberg SL 2012 Fast gapped-read alignment with Bowtie 2. Nat Methods 9:357–359. doi:10.1038/nmeth.1923. 22388286 PMC3322381

[61] Varela M, Chow YH, Sturkie C, Murcia P, Palmarini M 2006 Association of RON tyrosine kinase with the Jaagsiekte sheep retrovirus envelope glycoprotein. Virology 350:347–357. doi:10.1016/j.virol.2006.01.040. 16500691

[62] Mura M, Murcia P, Caporale M, Spencer TE, Nagashima K, Rein A, Palmarini M 2004 Late viral interference induced by transdominant Gag of an endogenous retrovirus. Proc Natl Acad Sci U S A 101:11117–11122. doi:10.1073/pnas.0402877101. 15263098 PMC503749

[63] Rusinova I, Forster S, Yu S, Kannan A, Masse M, Cumming H, Chapman R, Hertzog PJ 2013 Interferome v2.0: an updated database of annotated interferon-regulated genes. Nucleic Acids Res 41:D1040–D1046. doi:10.1093/nar/gks1215. 23203888 PMC3531205

[64] Huismans H 1979 Protein synthesis in bluetongue virus-infected cells. Virology 92:385–396. doi:10.1016/0042-6822(79)90143-0. 218351

[65] Huismans H 1971 Host cell protein synthesis after infection with bluetongue virus and reovirus. Virology 46:500–503. doi:10.1016/0042-6822(71)90053-5. 4331734

[66] Huismans H, van Dijk AA, Bauskin AR 1987 In vitro phosphorylation and purification of a nonstructural protein of bluetongue virus with affinity for single-stranded RNA. J Virol 61:3589–3595. 2822964 10.1128/jvi.61.11.3589-3595.1987 PMC255959

[67] Vinson C, Myakishev M, Acharya A, Mir AA, Moll JR, Bonovich M 2002 Classification of human B-ZIP proteins based on dimerization properties. Mol Cell Biol 22:6321–6335. doi:10.1128/MCB.22.18.6321-6335.2002. 12192032 PMC135624

[68] Hale BG 2014 Conformational plasticity of the influenza A virus NS1 protein. J Gen Virol 95:2099–2105. doi:10.1099/vir.0.066282-0. 24928909

[69] Krug RM 2015 Functions of the influenza A virus NS1 protein in antiviral defense. Curr Opin Virol 12:1–6. doi:10.1016/j.coviro.2015.01.007. 25638592 PMC4470714

[70] Poncet D, Aponte C, Cohen J 1993 Rotavirus protein NSP3 (NS34) is bound to the 3′ end consensus sequence of viral mRNAs in infected cells. J Virol 67:3159–3165. 8388495 10.1128/jvi.67.6.3159-3165.1993 PMC237654

[71] Poncet D, Laurent S, Cohen J 1994 Four nucleotides are the minimal requirement for RNA recognition by rotavirus non-structural protein NSP3. EMBO J 13:4165–4173. 8076612 10.1002/j.1460-2075.1994.tb06734.x PMC395339

