# Supplementary material for: Bluetongue Virus NS4 Protein Is an Interferon Antagonist and a Determinant of Virus Virulence
Source: J Virol. 2016 May 12;90(11):5427–39. doi: 10.1128/JVI.00422-16 (PMC4934764; doi:10.1128/JVI.00422-16)
Supplement: Supplemental material [file supp_90_11_5427__index.html]

Bluetongue Virus NS4 Protein Is an Interferon Antagonist and a Determinant of Virus Virulence — Supplemental material 

# Bluetongue Virus NS4 Protein Is an Interferon Antagonist and a Determinant of Virus Virulence

## Supplemental material

- Supplemental file 1 -

  Table S1 (Differentially expressed genes in BTV8wt- compared to mock-infected A549 cells.)

  Table S2 (Differentially expressed genes in BTV8ΔNS4- compared to mock-infected A549 cells.)

  Table S3 (Differentially expressed genes in BTV8ΔNS4- compared to BTV8wt-infected A549 cells.)

  PDF, 3.2M
