# Supplementary material for: Bluetongue Virus NS4 Protein Is an Interferon Antagonist and a Determinant of Virus Virulence
Source: J Virol. 2016 May 12;90(11):5427–39. doi: 10.1128/JVI.00422-16 (PMC4934764; doi:10.1128/JVI.00422-16)
Supplement: Supplemental material [file JVI.00422-16_zjv999181675so1.pdf]

Table S1. Differentially expressed genes in BTV8wt- compared to mock-infected A549 cells.

Gene symbol	Fold change (log2)	P-value [(a)]	Q-value [(b)] (<0.05)
GOLGA6B	5.29417	0.0042	0.029379
TNFSF4	5.1774	5.00E-05	0.000967
FOSB	5.13067	5.00E-05	0.000967
NID1	5.12621	5.00E-05	0.000967
OAS2	5.07037	5.00E-05	0.000967
EGR1	5.04055	5.00E-05	0.000967
KLRC4	5.01435	0.00075	0.008257
EGR2	4.99273	5.00E-05	0.000967
CA1	4.94783	5.00E-05	0.000967
KLRC2	4.85722	5.00E-05	0.000967
IFNL1	4.85015	5.00E-05	0.000967
LY6G6D	4.8236	0.0042	0.029379
OASL	4.82132	5.00E-05	0.000967
IFIT2	4.69685	5.00E-05	0.000967
FOS	4.6624	5.00E-05	0.000967
IL1RL1	4.63597	5.00E-05	0.000967
CCR4	4.58683	5.00E-05	0.000967
CH25H	4.56111	0.00295	0.022658
FAM71A	4.55619	5.00E-05	0.000967
HSPA6	4.51161	5.00E-05	0.000967
RAET1L	4.45823	0.0029	0.022408
IFIH1	4.45597	5.00E-05	0.000967
LY6G6C	4.44981	5.00E-05	0.000967
CXCL11	4.42392	0.00125	0.012208
IFI44	4.4202	5.00E-05	0.000967
TYRP1	4.3747	5.00E-05	0.000967
DUSP8	4.36605	0.0024	0.019631
FNDC7	4.33015	5.00E-05	0.000967
IL8	4.3058	5.00E-05	0.000967
FAM182B	4.29125	0.0002	0.003062
FAP	4.27405	5.00E-05	0.000967
LTA	4.25327	5.00E-05	0.000967
HSPA7	4.24313	5.00E-05	0.000967
MOBP	4.22447	5.00E-05	0.000967
HIST1H2BG	4.19766	5.00E-05	0.000967
IFIT1	4.18893	5.00E-05	0.000967
KIAA1045	4.18693	5.00E-05	0.000967
ARL14	4.18639	5.00E-05	0.000967
MX1	4.15104	5.00E-05	0.000967
IFIT3	4.14016	5.00E-05	0.000967
IFNB1	4.13006	5.00E-05	0.000967
AOC3	4.12595	5.00E-05	0.000967
TULP2	4.10406	5.00E-05	0.000967
MMP25	4.10341	5.00E-05	0.000967
C1orf189	4.09977	5.00E-05	0.000967

SLC1A3	4.06054	5.00E-05	0.000967
ALOXE3	4.05268	5.00E-05	0.000967
ZNF880	4.02872	0.00105	0.010623
GOLGA6C	4.02692	0.0001	0.001745
RSAD2	4.02467	5.00E-05	0.000967
KLRC3	4.02426	5.00E-05	0.000967
C20orf195	4.01489	5.00E-05	0.000967
ESR1	3.99705	5.00E-05	0.000967
CTRC	3.94739	0.0007	0.007858
LMOD2	3.94461	5.00E-05	0.000967
RFPL4A	3.92458	0.0005	0.006117
OR2B6	3.91393	5.00E-05	0.000967
SELPLG	3.90697	5.00E-05	0.000967
EGR4	3.89578	5.00E-05	0.000967
CCDC11	3.88493	5.00E-05	0.000967
CREB5	3.87514	5.00E-05	0.000967
HIST1H3G	3.84993	5.00E-05	0.000967
OVOL1	3.84897	5.00E-05	0.000967
KLRC1	3.84074	0.00025	0.003659
HDAC9	3.82778	5.00E-05	0.000967
KBTBD13	3.82493	5.00E-05	0.000967
C11orf91	3.82231	5.00E-05	0.000967
IFNL2	3.81902	5.00E-05	0.000967
HERC5	3.81038	5.00E-05	0.000967
IL17RE	3.80802	5.00E-05	0.000967
ARL14EPL	3.80413	0.00015	0.002431
ZNF300P1	3.79365	0.0001	0.001745
KRTAP5-9	3.78753	0.0056	0.036355
GIF	3.78503	0.00035	0.004678
TEX37	3.78491	0.0005	0.006117
MX2	3.76595	5.00E-05	0.000967
EPPK1	3.76031	5.00E-05	0.000967
ISG15	3.73909	5.00E-05	0.000967
CD200R1	3.72982	5.00E-05	0.000967
HPX	3.72707	5.00E-05	0.000967
NFE2	3.72239	5.00E-05	0.000967
C19orf69	3.71714	0.002	0.017269
IRGM	3.70614	5.00E-05	0.000967
GCG	3.70298	0.0025	0.02025
SLC6A12	3.67616	5.00E-05	0.000967
DSC1	3.67222	5.00E-05	0.000967
LOC100507206	3.66951	5.00E-05	0.000967
SCUBE2	3.64948	5.00E-05	0.000967
KDM6B	3.63942	5.00E-05	0.000967
C6orf25	3.63336	5.00E-05	0.000967
PLA2G4C	3.63276	5.00E-05	0.000967
LGR5	3.63262	5.00E-05	0.000967
OR5AU1	3.61021	0.0021	0.017919
CASS4	3.60942	5.00E-05	0.000967

TPRX1	3.60126	0.0006	0.007015
HIST1H2AE	3.59591	5.00E-05	0.000967
ADAMTSL4	3.59381	5.00E-05	0.000967
IFNL3	3.5908	5.00E-05	0.000967
OR13A1	3.58527	0.00015	0.002431
SPACA4	3.57752	5.00E-05	0.000967
CASQ1	3.57676	5.00E-05	0.000967
DHDH	3.56466	5.00E-05	0.000967
HK3	3.56078	0.00035	0.004678
OR2AE1	3.55476	0.0013	0.01252
ACTA1	3.54154	5.00E-05	0.000967
SLC6A13	3.51501	5.00E-05	0.000967
HCLS1	3.51402	5.00E-05	0.000967
FAM71F1	3.51359	5.00E-05	0.000967
SYT5	3.50348	5.00E-05	0.000967
FAM83E	3.4984	5.00E-05	0.000967
C3orf20	3.4976	5.00E-05	0.000967
CMPK2	3.48511	5.00E-05	0.000967
SLC16A12	3.47168	5.00E-05	0.000967
IFNA5	3.46386	0.004	0.02841
DDX58	3.46113	5.00E-05	0.000967
C6orf222	3.44699	5.00E-05	0.000967
DHX58	3.44683	5.00E-05	0.000967
TNF	3.43605	5.00E-05	0.000967
ITGAM	3.43508	5.00E-05	0.000967
MAFF	3.41791	5.00E-05	0.000967
TCTE1	3.41761	5.00E-05	0.000967
FCER1G	3.41274	0.00345	0.025496
TTC24	3.40816	5.00E-05	0.000967
OR1F2P	3.40691	5.00E-05	0.000967
FAM90A25P	3.39635	5.00E-05	0.000967
TMEM88	3.39178	5.00E-05	0.000967
C5orf48	3.3868	0.0035	0.025785
HIST1H1T	3.38114	5.00E-05	0.000967
LOC643201	3.37486	5.00E-05	0.000967
TRIM69	3.36928	5.00E-05	0.000967
FLJ12334	3.35901	5.00E-05	0.000967
TNFRSF9	3.3558	5.00E-05	0.000967
CCL5	3.34982	5.00E-05	0.000967
TMEM236	3.34382	5.00E-05	0.000967
C10orf120	3.3352	0.00335	0.024946
POPDC2	3.32977	5.00E-05	0.000967
SLCO5A1	3.32188	5.00E-05	0.000967
JUN	3.29393	5.00E-05	0.000967
RGMA	3.29325	5.00E-05	0.000967
ADAM21	3.29149	0.0001	0.001745
ANKRD1	3.29103	5.00E-05	0.000967
TG	3.28496	5.00E-05	0.000967
CD163L1	3.28335	5.00E-05	0.000967

CCDC144NL	3.28178	5.00E-05	0.000967
PLA2G12B	3.27595	0.00015	0.002431
LOC100128770	3.26979	5.00E-05	0.000967
CYLD	3.25114	5.00E-05	0.000967
RFPL3S	3.2484	5.00E-05	0.000967
ACTN2	3.24796	5.00E-05	0.000967
MYH15	3.24123	5.00E-05	0.000967
FCGR1B	3.23969	5.00E-05	0.000967
GPR152	3.23908	5.00E-05	0.000967
RNF112	3.22871	5.00E-05	0.000967
SV2A	3.22587	5.00E-05	0.000967
VSTM5	3.22523	0.00155	0.014354
C13orf45	3.21986	0.0001	0.001745
OR52I2	3.21474	0.00495	0.033213
HIST1H2AD	3.20276	0.0066	0.041051
TMCO2	3.20109	0.0054	0.035445
TRAF1	3.19978	5.00E-05	0.000967
PRDX2	3.18601	5.00E-05	0.000967
FOXS1	3.18318	5.00E-05	0.000967
C1orf162	3.18075	5.00E-05	0.000967
CCDC85B	3.17844	5.00E-05	0.000967
LRRC43	3.17194	0.0001	0.001745
ATP1A4	3.17016	5.00E-05	0.000967
IL32	3.15769	5.00E-05	0.000967
RASL10B	3.15756	5.00E-05	0.000967
LST1	3.15684	5.00E-05	0.000967
IFI27	3.15153	5.00E-05	0.000967
GPR50	3.13935	5.00E-05	0.000967
MYOZ3	3.13925	5.00E-05	0.000967
PKD1L1	3.1345	5.00E-05	0.000967
ZFPM2	3.1152	5.00E-05	0.000967
GPD1	3.11176	5.00E-05	0.000967
FBLL1	3.10854	5.00E-05	0.000967
TRHR	3.10532	5.00E-05	0.000967
ERP27	3.10336	0.0009	0.009427
CCDC64B	3.09802	0.002	0.017269
USH1G	3.09577	5.00E-05	0.000967
EGR3	3.09211	5.00E-05	0.000967
LMOD1	3.08821	5.00E-05	0.000967
CPA2	3.06952	0.00165	0.015053
GALR3	3.06679	5.00E-05	0.000967
LOC728175	3.06434	0.0001	0.001745
IL6	3.04262	5.00E-05	0.000967
CD79A	3.03582	5.00E-05	0.000967
GJB5	3.03473	0.0003	0.004153
DDX60	3.02631	5.00E-05	0.000967
OR1I1	3.01566	0.00295	0.022658
SLC6A4	3.00715	5.00E-05	0.000967
GPR26	3.00425	5.00E-05	0.000967

TSGA10IP	2.99703	5.00E-05	0.000967
CRABP2	2.99471	5.00E-05	0.000967
STC1	2.9795	5.00E-05	0.000967
TPPP3	2.9778	0.0001	0.001745
SLC28A1	2.97407	5.00E-05	0.000967
C5orf56	2.97356	5.00E-05	0.000967
IFNA8	2.97323	0.00185	0.016374
LOC100507140	2.97054	0.00035	0.004678
C17orf98	2.96746	0.0038	0.027341
IL23A	2.95271	5.00E-05	0.000967
OXER1	2.95221	5.00E-05	0.000967
LOC574538	2.94808	0.00085	0.009052
SRMS	2.93482	0.0006	0.007015
MEG9	2.93311	5.00E-05	0.000967
FAM209B	2.93068	5.00E-05	0.000967
MRGPRD	2.93043	0.0028	0.021921
EFNB2	2.92594	5.00E-05	0.000967
ARID5B	2.92321	5.00E-05	0.000967
ARL9	2.92096	5.00E-05	0.000967
TRPC7	2.91277	5.00E-05	0.000967
MYOC	2.90503	0.0003	0.004153
PLEKHA4	2.90482	5.00E-05	0.000967
SLC22A1	2.90308	5.00E-05	0.000967
ATF3	2.90299	5.00E-05	0.000967
SEMA4A	2.89964	5.00E-05	0.000967
NAT8	2.89788	0.00065	0.007427
LRIT3	2.8901	5.00E-05	0.000967
LDLRAD4	2.88847	5.00E-05	0.000967
ZC3HAV1	2.88678	5.00E-05	0.000967
EPHA8	2.88128	0.0001	0.001745
N4BP3	2.87916	5.00E-05	0.000967
DHRS2	2.87848	5.00E-05	0.000967
CNTD2	2.87737	5.00E-05	0.000967
NFKBIZ	2.87621	5.00E-05	0.000967
GPR84	2.87465	5.00E-05	0.000967
GSTM2P1	2.87316	5.00E-05	0.000967
SOCS1	2.87285	5.00E-05	0.000967
TNFAIP3	2.86829	5.00E-05	0.000967
NAV3	2.85706	5.00E-05	0.000967
GPBAR1	2.85539	5.00E-05	0.000967
GADL1	2.85417	5.00E-05	0.000967
HMGCS1	2.84885	5.00E-05	0.000967
CACNG1	2.84749	0.0001	0.001745
PNLDC1	2.84646	5.00E-05	0.000967
OIT3	2.84464	5.00E-05	0.000967
S100A14	2.84011	5.00E-05	0.000967
FAM71F2	2.83412	5.00E-05	0.000967
ADCY4	2.83292	5.00E-05	0.000967
IRF9	2.83041	5.00E-05	0.000967

LYST	2.8261	5.00E-05	0.000967
CCRN4L	2.82286	5.00E-05	0.000967
LAMB2P1	2.82214	5.00E-05	0.000967
MYPN	2.81597	5.00E-05	0.000967
IFNL4	2.79671	0.00065	0.007427
LRRC10	2.79634	5.00E-05	0.000967
MURC	2.78638	5.00E-05	0.000967
DDIT3	2.78466	0.00045	0.005638
DUSP10	2.78218	5.00E-05	0.000967
RASL12	2.77725	0.00315	0.023852
NPAS4	2.77609	0.00015	0.002431
IMPG2	2.77474	5.00E-05	0.000967
TBR1	2.76613	5.00E-05	0.000967
PARD6G-AS1	2.76479	0.00795	0.046985
RNF183	2.76063	5.00E-05	0.000967
TAS2R9	2.7602	5.00E-05	0.000967
CPT1C	2.75995	5.00E-05	0.000967
LRRC15	2.75902	5.00E-05	0.000967
GBP5	2.75849	5.00E-05	0.000967
MXD1	2.75752	5.00E-05	0.000967
SOD2	2.74126	5.00E-05	0.000967
ZEB2	2.73516	5.00E-05	0.000967
IRGC	2.73484	5.00E-05	0.000967
C6orf58	2.72753	0.0002	0.003062
LOC100507217	2.72481	5.00E-05	0.000967
ARHGAP40	2.72453	0.0001	0.001745
PLSCR2	2.72437	5.00E-05	0.000967
PCDH12	2.72329	5.00E-05	0.000967
GTF2IRD2	2.72105	5.00E-05	0.000967
PROZ	2.71604	5.00E-05	0.000967
B3GNT4	2.71518	5.00E-05	0.000967
CYP2G1P	2.70755	5.00E-05	0.000967
ZSCAN12P1	2.70728	5.00E-05	0.000967
GAS2L2	2.7047	5.00E-05	0.000967
EFNA2	2.70438	5.00E-05	0.000967
WFDC5	2.70136	0.00115	0.011454
ZNF382	2.69468	5.00E-05	0.000967
CELP	2.69317	0.0056	0.036355
ABAT	2.69032	5.00E-05	0.000967
NR4A3	2.68986	5.00E-05	0.000967
TMEM95	2.68519	5.00E-05	0.000967
FBXW12	2.67842	0.00075	0.008257
ALDOB	2.6775	5.00E-05	0.000967
H1FNT	2.67662	0.00085	0.009052
CATSPERD	2.67432	5.00E-05	0.000967
AOX1	2.67247	5.00E-05	0.000967
ARL10	2.66889	5.00E-05	0.000967
TREM2	2.66642	0.00175	0.015698
NRN1L	2.66523	0.00275	0.021673

ICAM1	2.66468	5.00E-05	0.000967
TAS2R7	2.66416	0.0001	0.001745
GYPE	2.66331	0.00035	0.004678
AADAC	2.65933	5.00E-05	0.000967
P2RY4	2.65755	0.0019	0.016639
SFTPA1	2.65231	5.00E-05	0.000967
TRIM54	2.65229	0.0003	0.004153
CTNNAL1	2.646	5.00E-05	0.000967
TBC1D29	2.63835	0.0008	0.008653
UPK1A	2.63736	0.00465	0.031738
S100A5	2.63503	5.00E-05	0.000967
SLCO1C1	2.63467	5.00E-05	0.000967
IFI6	2.63449	5.00E-05	0.000967
PPP1R15A	2.62734	5.00E-05	0.000967
N4BP2L1	2.62005	5.00E-05	0.000967
RELB	2.61828	5.00E-05	0.000967
LYPD3	2.61768	5.00E-05	0.000967
TLX2	2.61637	5.00E-05	0.000967
MYF6	2.61569	0.00335	0.024946
LINC00152	2.61465	5.00E-05	0.000967
GPR1	2.61381	5.00E-05	0.000967
HIST4H4	2.61201	5.00E-05	0.000967
BHLHE40-AS1	2.60794	0.0004	0.005167
BIRC3	2.60749	5.00E-05	0.000967
OVGP1	2.5984	5.00E-05	0.000967
PYGM	2.59554	5.00E-05	0.000967
MMRN2	2.59349	5.00E-05	0.000967
CHRM1	2.59309	5.00E-05	0.000967
LOC100287846	2.58963	5.00E-05	0.000967
TEX13B	2.58367	0.0033	0.024673
C5orf27	2.58206	5.00E-05	0.000967
FANK1	2.57982	5.00E-05	0.000967
ADIRF	2.57144	0.00025	0.003659
PLA1A	2.56318	0.00075	0.008257
WNT8A	2.54946	0.006	0.038323
PPP1R1C	2.54867	5.00E-05	0.000967
ANKRD34B	2.54436	5.00E-05	0.000967
TAC3	2.5439	0.0007	0.007858
FAM151B	2.54352	5.00E-05	0.000967
NHLH1	2.54113	5.00E-05	0.000967
FAM163A	2.53771	5.00E-05	0.000967
IFI16	2.5364	5.00E-05	0.000967
CYP2A6	2.53548	5.00E-05	0.000967
ITGA9	2.53014	5.00E-05	0.000967
LOC153910	2.52596	5.00E-05	0.000967
SOX30	2.52297	0.00015	0.002431
PPP4R4	2.51886	0.00015	0.002431
SBSN	2.51319	5.00E-05	0.000967
HIST1H2BF	2.50998	5.00E-05	0.000967

IL31RA	2.50977	5.00E-05	0.000967
ASB15	2.50905	5.00E-05	0.000967
CDKL4	2.50855	0.0002	0.003062
IL18RAP	2.5074	0.0001	0.001745
TEX35	2.50574	5.00E-05	0.000967
DAPP1	2.50571	5.00E-05	0.000967
PDE11A	2.50447	5.00E-05	0.000967
ACSM1	2.50406	0.00365	0.026607
SCN3A	2.50131	5.00E-05	0.000967
SLC39A2	2.49694	5.00E-05	0.000967
SHD	2.4945	5.00E-05	0.000967
PHLDA1	2.48433	5.00E-05	0.000967
CD83	2.48303	5.00E-05	0.000967
NIM1	2.48267	5.00E-05	0.000967
CLEC3B	2.47455	0.00625	0.03948
GJB4	2.4745	5.00E-05	0.000967
MIMT1	2.47013	0.0039	0.027879
GPR176	2.46749	5.00E-05	0.000967
HERC4	2.46558	5.00E-05	0.000967
SLC8A2	2.46461	5.00E-05	0.000967
CCL20	2.46301	5.00E-05	0.000967
LOC100130370	2.46125	0.00055	0.006573
DCHS1	2.45984	5.00E-05	0.000967
LOC100216546	2.45855	5.00E-05	0.000967
RHCG	2.45823	5.00E-05	0.000967
IL6ST	2.45323	5.00E-05	0.000967
CMTM2	2.45218	5.00E-05	0.000967
ZSCAN5B	2.4516	0.00025	0.003659
SLC2A9	2.4509	5.00E-05	0.000967
NEB	2.45069	5.00E-05	0.000967
LINC00486	2.44799	0.0072	0.043687
H2AFJ	2.44382	5.00E-05	0.000967
UBL4B	2.44231	0.00265	0.021074
TEKT2	2.43814	0.0005	0.006117
FOSL1	2.43529	5.00E-05	0.000967
APLF	2.4333	5.00E-05	0.000967
C3orf35	2.43104	5.00E-05	0.000967
SMLR1	2.43007	5.00E-05	0.000967
OR2B2	2.42738	5.00E-05	0.000967
ZSWIM4	2.42677	5.00E-05	0.000967
F13B	2.42653	5.00E-05	0.000967
TNNC2	2.42409	0.00065	0.007427
SNAPC1	2.42308	5.00E-05	0.000967
DQX1	2.41205	5.00E-05	0.000967
RPRML	2.41195	5.00E-05	0.000967
NR4A1	2.41092	5.00E-05	0.000967
LOC100499194	2.40999	5.00E-05	0.000967
IL13	2.40919	0.0001	0.001745
ALDH8A1	2.40693	5.00E-05	0.000967

PARD6G	2.4067	5.00E-05	0.000967
LRRIQ4	2.40603	0.00095	0.009766
ADAM32	2.4037	5.00E-05	0.000967
AOC4	2.40293	5.00E-05	0.000967
CLEC18A	2.40238	5.00E-05	0.000967
SLCO1B7	2.40236	5.00E-05	0.000967
DDX60L	2.40008	5.00E-05	0.000967
NPM2	2.39746	0.0083	0.048535
CCDC62	2.39726	5.00E-05	0.000967
ZNF610	2.39615	5.00E-05	0.000967
DIRC1	2.3902	0.0001	0.001745
CXCR4	2.38806	5.00E-05	0.000967
OR7E91P	2.37578	0.0009	0.009427
TEX29	2.37486	0.00815	0.047894
NPC1L1	2.37395	5.00E-05	0.000967
CYP2A7	2.36836	0.0005	0.006117
FAM109B	2.36519	0.0001	0.001745
LHFPL4	2.36263	5.00E-05	0.000967
IQSEC3	2.36131	5.00E-05	0.000967
PSD	2.35993	0.0038	0.027341
NEDD4L	2.35969	5.00E-05	0.000967
BEND4	2.35952	5.00E-05	0.000967
GCAT	2.35883	5.00E-05	0.000967
UNC5A	2.35485	0.00095	0.009766
VWA3A	2.35383	0.00015	0.002431
CLPS	2.35336	0.00115	0.011454
CYP21A1P	2.35173	0.0001	0.001745
PATL2	2.35119	0.00015	0.002431
TRIM40	2.35016	0.0002	0.003062
C7orf61	2.34591	0.0001	0.001745
PCP4L1	2.34495	0.0012	0.011824
SORBS1	2.34347	5.00E-05	0.000967
IFNA1	2.34261	0.0013	0.01252
RSPH4A	2.34071	5.00E-05	0.000967
PTPRH	2.33552	5.00E-05	0.000967
IFITM1	2.33359	0.0073	0.044132
OLFML2B	2.33215	5.00E-05	0.000967
IRF1	2.33121	5.00E-05	0.000967
MAP2K3	2.32745	5.00E-05	0.000967
IL11	2.32596	5.00E-05	0.000967
CAV3	2.32135	5.00E-05	0.000967
LOC100129924	2.31407	0.0002	0.003062
RSPH6A	2.31349	5.00E-05	0.000967
LOC100289187	2.30956	5.00E-05	0.000967
NLGN3	2.30633	5.00E-05	0.000967
HIST1H2AA	2.30607	0.0084	0.048965
LOC339807	2.30471	5.00E-05	0.000967
FGF23	2.30271	5.00E-05	0.000967
SEMA3D	2.30115	5.00E-05	0.000967

SAMD9	2.29472	5.00E-05	0.000967
HIST1H3D	2.29376	5.00E-05	0.000967
MFSD2B	2.29275	5.00E-05	0.000967
OR1J4	2.29163	0.0036	0.026323
ALPK3	2.28375	5.00E-05	0.000967
NODAL	2.28364	5.00E-05	0.000967
PLSCR1	2.28192	5.00E-05	0.000967
FOXD4L1	2.2811	5.00E-05	0.000967
TBX10	2.27754	5.00E-05	0.000967
LINC00565	2.277	0.00025	0.003659
ART4	2.27666	0.00015	0.002431
C17orf66	2.27518	5.00E-05	0.000967
HLA-B	2.26921	5.00E-05	0.000967
LINC00654	2.26905	0.00015	0.002431
PDZD2	2.26678	5.00E-05	0.000967
NKAPL	2.26578	5.00E-05	0.000967
ITGA5	2.26387	5.00E-05	0.000967
HELZ2	2.26379	5.00E-05	0.000967
GPR32	2.26285	0.0077	0.046014
ART1	2.26045	5.00E-05	0.000967
INHBA	2.25972	5.00E-05	0.000967
ERO1LB	2.25917	5.00E-05	0.000967
PDLIM4	2.25848	0.0013	0.01252
RNASE13	2.25846	0.00275	0.021673
JMJD1C	2.25736	5.00E-05	0.000967
LOC339166	2.25444	5.00E-05	0.000967
FUT5	2.25162	0.0005	0.006117
LTB	2.24993	0.00035	0.004678
TIGIT	2.24898	5.00E-05	0.000967
POU2F2	2.24817	5.00E-05	0.000967
ADAM20P1	2.24678	0.0002	0.003062
DNAH2	2.24657	5.00E-05	0.000967
FREM2	2.24039	5.00E-05	0.000967
C16orf46	2.23157	5.00E-05	0.000967
SSUH2	2.23127	5.00E-05	0.000967
ADAM20	2.23105	5.00E-05	0.000967
KCND3	2.22928	0.00115	0.011454
LINC00202-1	2.22886	5.00E-05	0.000967
ACR	2.22798	0.0084	0.048965
ESRRB	2.22241	5.00E-05	0.000967
NFKBIE	2.22195	5.00E-05	0.000967
GRK7	2.22123	5.00E-05	0.000967
HRH1	2.21965	5.00E-05	0.000967
SLC10A1	2.21698	5.00E-05	0.000967
PTAFR	2.21583	5.00E-05	0.000967
UBQLNL	2.21413	0.0003	0.004153
WNT2B	2.21195	5.00E-05	0.000967
LOC100505495	2.20492	5.00E-05	0.000967
SLC7A9	2.20483	5.00E-05	0.000967

LAMC2	2.20481	5.00E-05	0.000967
UBE2U	2.20271	0.0067	0.041533
LINC00641	2.20189	5.00E-05	0.000967
ISG20	2.20131	5.00E-05	0.000967
MYO1A	2.20002	5.00E-05	0.000967
SPRED3	2.19734	5.00E-05	0.000967
GRK4	2.19454	0.00015	0.002431
LOC388942	2.191	5.00E-05	0.000967
C8orf46	2.18921	5.00E-05	0.000967
ZNF501	2.18619	0.0001	0.001745
AKAP12	2.17973	5.00E-05	0.000967
FGF18	2.1773	0.00025	0.003659
PTGER4	2.17719	5.00E-05	0.000967
SEMA7A	2.17429	5.00E-05	0.000967
MRPL23-AS1	2.17395	5.00E-05	0.000967
LOC440896	2.17149	5.00E-05	0.000967
CORO2B	2.17098	5.00E-05	0.000967
CSRNP1	2.17014	5.00E-05	0.000967
BCAR4	2.16529	0.00055	0.006573
SRD5A2	2.16364	0.00095	0.009766
C7orf53	2.16249	5.00E-05	0.000967
PYCARD	2.1614	5.00E-05	0.000967
MPZL3	2.16088	5.00E-05	0.000967
TRIM72	2.15779	0.0007	0.007858
CHRNB2	2.15282	5.00E-05	0.000967
LDHAL6A	2.15257	5.00E-05	0.000967
SLCO1A2	2.14748	5.00E-05	0.000967
HIVEP2	2.14713	5.00E-05	0.000967
NLRP9	2.1445	5.00E-05	0.000967
NMRK2	2.14287	0.00125	0.012208
LZTS1	2.14268	5.00E-05	0.000967
GJC2	2.13892	5.00E-05	0.000967
ACVR1C	2.13853	0.0001	0.001745
PDZD9	2.13767	5.00E-05	0.000967
NLRP7	2.13426	0.00015	0.002431
SPAG9	2.13314	5.00E-05	0.000967
HAS2	2.13268	5.00E-05	0.000967
KLF6	2.13199	5.00E-05	0.000967
B3GALNT2	2.12953	5.00E-05	0.000967
APOE	2.12934	5.00E-05	0.000967
MTNR1A	2.12886	5.00E-05	0.000967
FAM154B	2.12861	5.00E-05	0.000967
C2orf83	2.12625	5.00E-05	0.000967
ACSBG1	2.12588	5.00E-05	0.000967
SAMD7	2.1255	0.00295	0.022658
UCN2	2.12469	5.00E-05	0.000967
SLC22A14	2.1244	0.0007	0.007858
RSPO1	2.12095	0.00015	0.002431
NCF1B	2.12044	0.00205	0.017591

OSM	2.11952	5.00E-05	0.000967
TLR9	2.11941	5.00E-05	0.000967
PHLDA2	2.11781	5.00E-05	0.000967
CYR61	2.11721	5.00E-05	0.000967
SYT11	2.11602	5.00E-05	0.000967
MCTP1	2.11475	5.00E-05	0.000967
COL11A2	2.1146	0.0003	0.004153
NEK10	2.11305	5.00E-05	0.000967
FAM90A1	2.11043	0.00035	0.004678
IL21R-AS1	2.11036	0.0003	0.004153
ST3GAL6	2.10989	5.00E-05	0.000967
CDO1	2.10754	0.00205	0.017591
BATF2	2.10489	5.00E-05	0.000967
TAS2R8	2.0953	0.00245	0.019913
LOC100216545	2.09353	5.00E-05	0.000967
POU3F4	2.09176	0.0006	0.007015
OR1F1	2.08628	5.00E-05	0.000967
PTPRN2	2.08571	0.0007	0.007858
MAGEA8	2.08487	0.0001	0.001745
SLC45A2	2.08454	0.00055	0.006573
FOXD4L6	2.07969	5.00E-05	0.000967
CDKL3	2.07961	5.00E-05	0.000967
GPR61	2.07777	0.0001	0.001745
MCL1	2.07401	5.00E-05	0.000967
TSPO2	2.07078	0.00045	0.005638
LOC339894	2.0701	0.0004	0.005167
OAS1	2.06867	5.00E-05	0.000967
CILP	2.0674	5.00E-05	0.000967
PLBD1	2.06539	0.0001	0.001745
PARP9	2.06406	0.00665	0.041284
CELF6	2.06203	5.00E-05	0.000967
PFKFB4	2.06131	5.00E-05	0.000967
CLEC18C	2.0593	0.00265	0.021074
SPTSSB	2.05786	0.0003	0.004153
TNFAIP6	2.05772	0.0085	0.04934
RSPH10B2	2.05663	5.00E-05	0.000967
S100A2	2.05613	5.00E-05	0.000967
C2orf27B	2.05529	0.0047	0.031987
RDH12	2.05508	0.00015	0.002431
LOC285627	2.05285	5.00E-05	0.000967
PLA2G3	2.05272	0.00175	0.015698
PINLYP	2.05231	0.0004	0.005167
FOXD4L5	2.04942	5.00E-05	0.000967
GTF2IRD2B	2.04732	5.00E-05	0.000967
CHD2	2.04513	5.00E-05	0.000967
CEACAM20	2.04479	0.00175	0.015698
IFI44L	2.0444	5.00E-05	0.000967
NR1D1	2.04357	0.00025	0.003659
C2orf61	2.04302	5.00E-05	0.000967

MYLK2	2.04081	5.00E-05	0.000967
SLC25A34	2.04004	5.00E-05	0.000967
LINC00622	2.03941	0.00135	0.012838
C7orf63	2.03892	5.00E-05	0.000967
RRH	2.03219	0.0041	0.028947
CSF3	2.02984	5.00E-05	0.000967
RAPSN	2.02861	5.00E-05	0.000967
NFKB1	2.02855	5.00E-05	0.000967
ZNF80	2.02645	5.00E-05	0.000967
HCAR3	2.02166	5.00E-05	0.000967
CSF2	2.01884	0.00865	0.049984
ETS1	2.01587	5.00E-05	0.000967
OAS3	2.01535	5.00E-05	0.000967
NCOR1P1	2.01378	0.00245	0.019913
LRRC66	2.01164	5.00E-05	0.000967
GALNT3	2.0115	0.0004	0.005167
ARC	2.01094	5.00E-05	0.000967
LOC100862671	2.00839	5.00E-05	0.000967
SNCG	2.00833	0.00015	0.002431
LINC00162	2.00771	0.00675	0.041733
FSCN3	2.0052	0.0002	0.003062
ISLR	2.00421	5.00E-05	0.000967
GUCA1B	2.00346	5.00E-05	0.000967
ADIG	2.00243	0.00695	0.04259
KIAA1549L	2.00138	5.00E-05	0.000967
GRIA4	2.00022	5.00E-05	0.000967
AVPR2	1.99744	0.0013	0.01252
LOC339666	1.99668	5.00E-05	0.000967
ACTL7A	1.99628	0.00105	0.010623
HLA-E	1.99587	5.00E-05	0.000967
DUSP6	1.99509	5.00E-05	0.000967
FRZB	1.99206	0.0003	0.004153
RPL21P44	1.99161	5.00E-05	0.000967
LOC646498	1.99083	0.00685	0.042163
LINC00672	1.98944	5.00E-05	0.000967
CILP2	1.98809	5.00E-05	0.000967
NMNAT2	1.98637	5.00E-05	0.000967
SERTAD1	1.98525	5.00E-05	0.000967
GPR3	1.98299	5.00E-05	0.000967
LAMP5	1.97685	0.00045	0.005638
ACRBP	1.97424	5.00E-05	0.000967
HERC6	1.97148	5.00E-05	0.000967
APOBEC3B	1.9707	5.00E-05	0.000967
STARD13	1.97012	5.00E-05	0.000967
PRDM7	1.96992	5.00E-05	0.000967
TFPI	1.96987	5.00E-05	0.000967
GUCA1A	1.96697	0.00095	0.009766
PRKACG	1.96692	0.00225	0.01885
LINC00854	1.96416	5.00E-05	0.000967

KCNN2	1.96395	5.00E-05	0.000967
GUCY2D	1.9575	0.00035	0.004678
BEST2	1.95582	0.0026	0.020846
EXOC3L2	1.95543	5.00E-05	0.000967
APOL3	1.95447	0.00045	0.005638
HRH3	1.95408	0.0003	0.004153
DUSP15	1.9456	0.0012	0.011824
FOXD4L3	1.94327	0.0001	0.001745
PLCB4	1.94309	5.00E-05	0.000967
LOC100129722	1.94258	0.00105	0.010623
CCDC19	1.9405	0.00015	0.002431
RNF223	1.94049	0.00635	0.03999
PTGS2	1.93826	5.00E-05	0.000967
LOC100506136	1.93621	5.00E-05	0.000967
GPBP1	1.93315	5.00E-05	0.000967
DNAH1	1.93178	5.00E-05	0.000967
HIST2H2BC	1.92959	5.00E-05	0.000967
HAPLN2	1.92818	0.0002	0.003062
SLC30A7	1.92741	5.00E-05	0.000967
RSRC2	1.9264	5.00E-05	0.000967
POU5F1P3	1.92586	0.00635	0.03999
PER1	1.92501	5.00E-05	0.000967
NUAK2	1.92487	5.00E-05	0.000967
ARID4B	1.92424	5.00E-05	0.000967
HIST1H4E	1.92257	5.00E-05	0.000967
FAM46C	1.91907	5.00E-05	0.000967
HSPH1	1.91886	5.00E-05	0.000967
TOP1	1.91766	5.00E-05	0.000967
CPM	1.91313	5.00E-05	0.000967
IER2	1.90993	5.00E-05	0.000967
TTC9B	1.90916	0.0019	0.016639
DEF6	1.90906	0.0051	0.033918
CRTAM	1.90784	0.0001	0.001745
TREML1	1.90511	0.00195	0.016979
MTVR2	1.90112	5.00E-05	0.000967
NLRC4	1.90056	0.0001	0.001745
UBASH3A	1.89848	0.00135	0.012838
NFKBIB	1.89763	5.00E-05	0.000967
MEG8	1.89675	0.00285	0.022187
GHSR	1.8966	0.00325	0.024387
KCNH7	1.89649	0.00055	0.006573
ARL5B	1.89341	5.00E-05	0.000967
LOC554223	1.89145	0.0076	0.045548
LEAP2	1.89031	5.00E-05	0.000967
LOC100271702	1.89021	0.0025	0.02025
C12orf70	1.88939	0.0057	0.036875
C4A,C4B_2	1.88933	0.0039	0.027879
IRS4	1.888	0.0011	0.011035
KIAA0825	1.88427	0.0001	0.001745

OR2H2	1.8818	0.0068	0.041918
BCL2A1	1.88146	5.00E-05	0.000967
H3F3B	1.88127	5.00E-05	0.000967
CLEC18B	1.8804	0.00165	0.015053
NPR2	1.87871	5.00E-05	0.000967
HDX	1.87633	5.00E-05	0.000967
IL21R	1.87576	0.0028	0.021921
SLC9C1	1.87454	0.0015	0.013976
PAX6	1.87299	5.00E-05	0.000967
PGM5	1.87047	0.00075	0.008257
UCMA	1.86943	0.00335	0.024946
PTPRE	1.86839	0.00035	0.004678
COL12A1	1.86668	5.00E-05	0.000967
NFKB2	1.86656	5.00E-05	0.000967
ZSCAN10	1.86539	0.0012	0.011824
CKM	1.8652	0.00135	0.012838
CTRB1	1.86516	0.0058	0.037289
VGF	1.86495	5.00E-05	0.000967
LOC100506305	1.86261	5.00E-05	0.000967
AZI2	1.86107	5.00E-05	0.000967
ANKDD1B	1.85986	0.00085	0.009052
AOC2	1.85915	5.00E-05	0.000967
HTR2A	1.85863	0.0003	0.004153
NXPH3	1.8554	5.00E-05	0.000967
NFE2L3	1.85469	5.00E-05	0.000967
KPNA7	1.8546	5.00E-05	0.000967
GADD45A	1.85426	5.00E-05	0.000967
ASB2	1.8541	0.0004	0.005167
RASD1	1.84893	5.00E-05	0.000967
GRM2	1.84796	0.0013	0.01252
CCNL1	1.84595	5.00E-05	0.000967
MTHFD2L	1.84529	0.00025	0.003659
DNAH17	1.84404	0.0033	0.024673
DLL4	1.8438	0.0001	0.001745
DNAJB5	1.84311	5.00E-05	0.000967
ABL2	1.84251	5.00E-05	0.000967
SOST	1.84205	0.0014	0.013185
SPRY2	1.8416	5.00E-05	0.000967
TAS2R42	1.84064	0.0029	0.022408
TTC21A	1.8399	5.00E-05	0.000967
MPP4	1.83794	0.00035	0.004678
CPLX3	1.8348	0.00245	0.019913
LHX3	1.83376	0.0015	0.013976
MAP3K19	1.83324	0.00265	0.021074
ANXA5	1.83314	5.00E-05	0.000967
CD226	1.83083	0.0003	0.004153
HIST1H4H	1.83058	5.00E-05	0.000967
XIRP1	1.83005	0.00045	0.005638
WDR66	1.82924	5.00E-05	0.000967

CRISPLD2	1.82805	0.0001	0.001745
RUNX1	1.82595	5.00E-05	0.000967
TGIF2	1.82535	0.0073	0.044132
WDR96	1.82393	0.0003	0.004153
PTH1R	1.82225	0.00095	0.009766
TAS2R5	1.82193	5.00E-05	0.000967
MARK2P9	1.8209	0.0008	0.008653
LINC00483	1.82018	5.00E-05	0.000967
HAVCR2	1.81707	0.0004	0.005167
THOC6	1.81703	0.0001	0.001745
FAAH	1.81583	0.0009	0.009427
GTF2IRD2P1	1.81472	5.00E-05	0.000967
LINC00624	1.81315	0.00065	0.007427
SYNE1	1.81312	5.00E-05	0.000967
LOC646862	1.81153	0.00035	0.004678
NFKBIA	1.81012	5.00E-05	0.000967
CNTN2	1.80974	5.00E-05	0.000967
PKI55	1.80909	5.00E-05	0.000967
TSPYL2	1.80849	5.00E-05	0.000967
CNN1	1.80567	0.002	0.017269
LATS2	1.80539	5.00E-05	0.000967
GUSBP5	1.80213	0.00065	0.007427
RASGRP2	1.80181	0.0005	0.006117
ANKRD10	1.80074	5.00E-05	0.000967
ZFP36	1.79921	5.00E-05	0.000967
FLJ33581	1.79817	0.0028	0.021921
ST7L	1.79721	5.00E-05	0.000967
SLC25A2	1.79712	0.0002	0.003062
LAMB3	1.79565	5.00E-05	0.000967
TAAR3	1.79464	0.0068	0.041918
ICAM4	1.79362	0.00025	0.003659
CALHM1	1.79248	5.00E-05	0.000967
GPR179	1.79095	5.00E-05	0.000967
PCF11	1.78944	5.00E-05	0.000967
SKAP1	1.78904	0.0023	0.019134
ATP2B2	1.78582	0.0001	0.001745
LRRC4B	1.78325	5.00E-05	0.000967
SEMA6A	1.78087	5.00E-05	0.000967
LOC643669	1.77901	5.00E-05	0.000967
ROPN1L	1.77791	0.00075	0.008257
LPAR5	1.7755	0.00055	0.006573
WDR26	1.77395	5.00E-05	0.000967
MFRP	1.77247	0.0002	0.003062
PODXL	1.77157	5.00E-05	0.000967
SNX29P2	1.77067	0.0002	0.003062
PARP8	1.77036	0.00015	0.002431
C16orf90	1.77011	0.00135	0.012838
KLF10	1.76994	5.00E-05	0.000967
TEX14	1.76906	0.0001	0.001745

GALR2	1.76696	0.0003	0.004153
TBC1D28	1.76565	0.00535	0.035214
CYP2E1	1.76556	0.0032	0.024153
PDE2A	1.76408	0.0001	0.001745
H3F3C	1.76378	5.00E-05	0.000967
CREBRF	1.76355	0.00025	0.003659
LINC00346	1.7627	5.00E-05	0.000967
TOP1P1	1.7627	5.00E-05	0.000967
FAM180B	1.75928	0.00035	0.004678
CLDN6	1.75924	0.00095	0.009766
ABCC11	1.75591	0.0002	0.003062
HCAR2	1.75571	0.0002	0.003062
TMEM198	1.75548	0.0002	0.003062
CDR1	1.75462	0.00025	0.003659
FOXD4L2	1.75408	5.00E-05	0.000967
KLHL41	1.75406	0.0004	0.005167
RSPRY1	1.75335	5.00E-05	0.000967
PARP14	1.75145	5.00E-05	0.000967
FST	1.75036	5.00E-05	0.000967
MUC20	1.74986	5.00E-05	0.000967
LOC100507489	1.74953	0.00415	0.029152
KLHL4	1.74894	5.00E-05	0.000967
TMEM151B	1.74775	0.0003	0.004153
XRN1	1.74482	5.00E-05	0.000967
ALMS1P	1.74479	0.00505	0.033693
RIOK3	1.74376	5.00E-05	0.000967
CXCL1	1.7426	5.00E-05	0.000967
DLX2	1.7411	0.00015	0.002431
EEA1	1.74057	5.00E-05	0.000967
OR52W1	1.73923	0.00675	0.041733
MAP1LC3B	1.73669	0.00015	0.002431
XDH	1.73488	5.00E-05	0.000967
H1F0	1.73467	5.00E-05	0.000967
SCN5A	1.73393	0.0001	0.001745
CCDC17	1.73191	5.00E-05	0.000967
CFP	1.73079	0.0004	0.005167
TRA2A	1.72883	5.00E-05	0.000967
KCNQ3	1.72705	0.0001	0.001745
SFRP5	1.72627	0.0001	0.001745
NRADDP	1.72404	0.00085	0.009052
SBSPON	1.72293	0.00015	0.002431
DCAF4L1	1.71954	5.00E-05	0.000967
ELL2	1.71877	5.00E-05	0.000967
PRAM1	1.71707	0.0034	0.025194
THAP1	1.71697	5.00E-05	0.000967
SEC24A	1.71508	5.00E-05	0.000967
FABP2	1.71413	0.001	0.010186
C22orf34	1.71197	0.0032	0.024153
MAATS1	1.71123	0.00325	0.024387

FRMD6	1.71072	5.00E-05	0.000967
ZNF474	1.71055	0.0001	0.001745
NFAT5	1.71002	5.00E-05	0.000967
TMEM163	1.70836	0.001	0.010186
RYR1	1.70692	5.00E-05	0.000967
FAM117A	1.70617	5.00E-05	0.000967
TRPC5	1.70443	0.0005	0.006117
LRGUK	1.70382	0.00055	0.006573
REM2	1.70358	5.00E-05	0.000967
LOC100288181	1.70208	0.0001	0.001745
CYP1A1	1.70183	5.00E-05	0.000967
PGPEP1L	1.69932	0.00395	0.028152
MICALCL	1.69858	0.00055	0.006573
CDKN1C	1.69805	0.00235	0.019365
CHRNA10	1.69796	5.00E-05	0.000967
USP53	1.69779	5.00E-05	0.000967
ZCCHC6	1.69757	5.00E-05	0.000967
APOF	1.69709	0.00045	0.005638
ADAM12	1.69576	0.00015	0.002431
NAALAD2	1.69391	5.00E-05	0.000967
SDCBP	1.69356	5.00E-05	0.000967
CXorf36	1.69339	0.0002	0.003062
SHISA4	1.69328	5.00E-05	0.000967
LOC339593	1.69301	0.0042	0.029379
IGF2BP2	1.69155	5.00E-05	0.000967
SERPINA10	1.69028	0.0005	0.006117
ACTN3	1.68753	0.00045	0.005638
HIST1H4J	1.6875	0.00025	0.003659
PARP12	1.68725	5.00E-05	0.000967
HIST1H4K	1.68585	0.0001	0.001745
ATCAY	1.68477	5.00E-05	0.000967
LOC541471	1.6842	5.00E-05	0.000967
TRIM55	1.68362	0.0029	0.022408
FAM133DP	1.68345	0.0003	0.004153
ZNFX1	1.68339	5.00E-05	0.000967
LINC00163	1.68262	0.00065	0.007427
DUSP5	1.68253	5.00E-05	0.000967
BCL2L12	1.68137	0.00235	0.019365
CLDN24	1.68025	0.0003	0.004153
TBX6	1.68011	0.00165	0.015053
GDF9	1.68004	0.0002	0.003062
NPTX1	1.6795	5.00E-05	0.000967
HIST1H4C	1.6781	5.00E-05	0.000967
DDAH2	1.67773	5.00E-05	0.000967
TNFSF14	1.67762	0.00015	0.002431
EIF2AK2	1.67691	5.00E-05	0.000967
INPP1	1.67648	5.00E-05	0.000967
FAM46B	1.67603	5.00E-05	0.000967
SYNJ1	1.67462	5.00E-05	0.000967

ETV7	1.67397	0.0008	0.008653
PPM1K	1.67366	5.00E-05	0.000967
LOC100128288	1.67357	5.00E-05	0.000967
TAS2R4	1.67274	5.00E-05	0.000967
TKTL1	1.67219	0.0017	0.015408
DAW1	1.67056	0.00345	0.025496
GPM6B	1.66856	0.0004	0.005167
NME5	1.66789	0.0058	0.037289
REL	1.66625	5.00E-05	0.000967
BST1	1.66587	5.00E-05	0.000967
LAMTOR3	1.6644	0.0001	0.001745
SEMA3A	1.66439	5.00E-05	0.000967
RPL13AP20	1.66206	0.00015	0.002431
IFFO1	1.66061	5.00E-05	0.000967
RFTN2	1.65937	5.00E-05	0.000967
CD160	1.65661	0.0001	0.001745
LOC401242	1.65645	0.00085	0.009052
FNIP1	1.65009	5.00E-05	0.000967
GNG4	1.65008	0.0001	0.001745
GRHL2	1.64879	0.00085	0.009052
STX3	1.6484	5.00E-05	0.000967
CLK1	1.64731	5.00E-05	0.000967
NCOA7	1.64662	5.00E-05	0.000967
POU1F1	1.64405	0.0019	0.016639
CA13	1.64166	5.00E-05	0.000967
EPGN	1.64035	0.0002	0.003062
PIWIL2	1.63685	0.0001	0.001745
SLC22A13	1.63646	0.0003	0.004153
C19orf38	1.63631	0.00535	0.035214
LINC00521	1.63566	0.0019	0.016639
CLEC3A	1.63565	0.0074	0.044655
CPEB3	1.6351	5.00E-05	0.000967
AKD1	1.63221	0.0003	0.004153
TRANK1	1.63079	5.00E-05	0.000967
RCBTB2	1.63022	0.00025	0.003659
LINC-ROR	1.63001	0.00095	0.009766
CNBD2	1.62915	0.0053	0.034982
ZNF655	1.62823	0.0001	0.001745
BCL3	1.62725	5.00E-05	0.000967
TMEM86A	1.62694	0.004	0.02841
PGM5P2	1.62568	5.00E-05	0.000967
CCDC50	1.62471	5.00E-05	0.000967
DHRS9	1.62399	0.0026	0.020846
SUSD3	1.62217	0.00695	0.04259
GPSM3	1.62171	0.00695	0.04259
EVA1B	1.62164	0.00215	0.018214
FUT3	1.6214	0.0009	0.009427
YTHDC1	1.62122	5.00E-05	0.000967
ZMYND10	1.62122	0.00095	0.009766

HCP5	1.62055	0.0022	0.018543
SELT	1.6193	5.00E-05	0.000967
SLC2A12	1.61816	5.00E-05	0.000967
C12orf50	1.61805	0.0079	0.046806
BRAF	1.61777	5.00E-05	0.000967
SPRED2	1.61754	5.00E-05	0.000967
HLA-H	1.61666	0.0002	0.003062
COL17A1	1.61562	0.00015	0.002431
TRPM5	1.61145	0.00025	0.003659
C15orf27	1.60947	5.00E-05	0.000967
CD274	1.60869	0.0001	0.001745
APOL5	1.60653	0.0086	0.049747
SLC25A18	1.60634	0.00015	0.002431
ANTXR2	1.60601	0.00155	0.014354
FITM1	1.60367	0.0078	0.046428
CD14	1.6034	0.0004	0.005167
MYO15A	1.60245	0.0001	0.001745
ZBP1	1.60101	0.0016	0.014734
AKT1S1	1.59982	5.00E-05	0.000967
ROCK1P1	1.59933	0.0005	0.006117
CPEB2	1.59927	5.00E-05	0.000967
COL8A2	1.59795	5.00E-05	0.000967
CDS1	1.59772	0.0003	0.004153
TSC22D1	1.59674	5.00E-05	0.000967
SERPINB8	1.59658	5.00E-05	0.000967
NEXN	1.59582	0.0002	0.003062
TBX15	1.59568	0.00065	0.007427
DDX25	1.59461	0.0061	0.038798
PIP5K1A	1.59316	5.00E-05	0.000967
ECM1	1.59221	0.00025	0.003659
HSPB9	1.59207	0.00205	0.017591
CAMK1G	1.58774	0.00235	0.019365
RXFP4	1.58732	0.0003	0.004153
OXTR	1.58305	5.00E-05	0.000967
TMEM106A	1.58292	5.00E-05	0.000967
B2M	1.58287	5.00E-05	0.000967
C8orf86	1.58232	0.0007	0.007858
SAMHD1	1.5818	0.0002	0.003062
SOAT2	1.57941	0.00355	0.026073
C6orf99	1.57906	0.00195	0.016979
MTSS1	1.57904	5.00E-05	0.000967
NRIP3	1.57745	5.00E-05	0.000967
SLC10A5	1.57697	0.0019	0.016639
HLA-A	1.57696	5.00E-05	0.000967
GNRHR2	1.5763	0.0038	0.027341
ZNF547	1.57551	0.00505	0.033693
CUL3	1.57421	0.0002	0.003062
SOWAHD	1.57282	0.00105	0.010623
ITGA10	1.57261	5.00E-05	0.000967

GJA4	1.57166	0.0065	0.040703
ING3	1.57079	5.00E-05	0.000967
MSANTD3	1.57078	5.00E-05	0.000967
ABI3BP	1.57011	0.00095	0.009766
VAMP2	1.56835	5.00E-05	0.000967
ERV3-1	1.56767	0.0058	0.037289
CD59	1.56753	5.00E-05	0.000967
DUOXA1	1.56675	0.00315	0.023852
PRSS37	1.56513	0.00175	0.015698
MSH4	1.56463	0.00025	0.003659
SLC36A2	1.56463	0.00025	0.003659
SLC17A9	1.56413	0.00395	0.028152
IGF2	1.5634	0.00685	0.042163
AP3S1	1.56311	0.0005	0.006117
NCCRP1	1.56307	0.0008	0.008653
GFRA3	1.5626	0.002	0.017269
GPR37L1	1.56182	0.0007	0.007858
LOC100292680	1.56006	0.0001	0.001745
PSORS1C3	1.55822	0.0003	0.004153
CYP3A5	1.55773	0.00045	0.005638
GPR4	1.55557	0.00085	0.009052
SLCO1B1	1.55476	0.0005	0.006117
CFB	1.55349	0.0002	0.003062
ZC3H11A	1.55249	0.00015	0.002431
DDA1	1.55152	0.0001	0.001745
DUOX2	1.55036	0.00065	0.007427
OTUD1	1.54965	5.00E-05	0.000967
TINCR	1.5496	0.0001	0.001745
TSPAN12	1.54768	0.0071	0.043222
PLIN1	1.54647	0.00095	0.009766
STX11	1.54593	0.00015	0.002431
ADRB2	1.5455	0.00155	0.014354
FAM83C	1.54469	0.00045	0.005638
PGF	1.54465	0.0008	0.008653
P4HA1	1.54367	5.00E-05	0.000967
PDE6G	1.54362	0.00145	0.01361
CTSK	1.54274	5.00E-05	0.000967
SETX	1.54267	5.00E-05	0.000967
LOC441155	1.54192	0.00015	0.002431
DDX26B	1.54018	0.00165	0.015053
BBC3	1.53954	0.00015	0.002431
SERPINA1	1.53843	0.0006	0.007015
TDRD7	1.53836	5.00E-05	0.000967
LINC00525	1.53664	0.0009	0.009427
ZNF682	1.53378	0.00105	0.010623
LRRC71	1.53264	0.0049	0.032984
CLTB	1.53239	0.0001	0.001745
CYP27B1	1.53229	5.00E-05	0.000967
RFPL2	1.53215	0.0016	0.014734

TRIP12	1.52764	5.00E-05	0.000967
CDK7	1.52723	5.00E-05	0.000967
GABRR2	1.52381	0.00615	0.039026
PRPH2	1.52341	0.00065	0.007427
WNT8B	1.52196	0.0021	0.017919
KCND1	1.52085	5.00E-05	0.000967
LETM2	1.52	0.00015	0.002431
CCDC173	1.51698	0.0066	0.041051
SP110	1.51694	0.0001	0.001745
HSD3BP4	1.51667	0.00035	0.004678
CD247	1.51659	0.0028	0.021921
APPBP2	1.51611	5.00E-05	0.000967
DNAJC27-AS1	1.51522	0.00025	0.003659
LOC400027	1.51475	5.00E-05	0.000967
NACAD	1.51389	0.00245	0.019913
NUDT8	1.51386	0.00165	0.015053
ANPEP	1.51292	0.0001	0.001745
LOX	1.511	0.0002	0.003062
MYOZ1	1.51014	0.00675	0.041733
CREB3L1	1.50704	0.0062	0.039223
NR1D2	1.50637	5.00E-05	0.000967
CSPG5	1.50556	0.0013	0.01252
CTGF	1.50545	5.00E-05	0.000967
MTHFR	1.50413	0.0007	0.007858
ACHE	1.5036	0.0009	0.009427
PPP6C	1.50316	0.00035	0.004678
CECR2	1.50079	0.0008	0.008653
GFRA1	1.49991	5.00E-05	0.000967
SEMA4D	1.49949	5.00E-05	0.000967
LECT1	1.49778	0.00485	0.03274
PLB1	1.49748	0.00075	0.008257
THEMIS2	1.49735	0.0012	0.011824
SCYL2	1.49574	5.00E-05	0.000967
MYH3	1.49517	0.0001	0.001745
TBX4	1.49411	0.00215	0.018214
ZSCAN5A	1.4932	0.0001	0.001745
USP6	1.49281	0.00045	0.005638
CELF2	1.49268	0.00435	0.030187
LTBP2	1.49039	5.00E-05	0.000967
RRAD	1.48987	0.0047	0.031987
LINC00470	1.48944	0.00055	0.006573
POU3F1	1.48775	0.00485	0.03274
KDM5B	1.48712	5.00E-05	0.000967
PRKXP1	1.48689	0.00015	0.002431
HIST1H2BK	1.48605	0.0002	0.003062
CCT6B	1.48333	0.0011	0.011035
ITGAV	1.48259	5.00E-05	0.000967
DKK3	1.4823	0.0002	0.003062
RBM39	1.48197	5.00E-05	0.000967

ITGA2B	1.48145	0.0012	0.011824
MZB1	1.48035	0.00235	0.019365
LOC149134	1.47885	0.00185	0.016374
WHAMM	1.476	0.0001	0.001745
MASP2	1.47562	0.00025	0.003659
BTN2A2	1.47389	5.00E-05	0.000967
C11orf68	1.47302	0.00035	0.004678
PRDM6	1.47228	0.00165	0.015053
KLF4	1.47118	5.00E-05	0.000967
DDX10	1.47066	0.0001	0.001745
FBLN5	1.47035	0.0034	0.025194
KDM6A	1.47019	5.00E-05	0.000967
TRABD2A	1.47001	0.00025	0.003659
CUBN	1.46998	0.0002	0.003062
PTHLH	1.4696	0.0003	0.004153
LAT2	1.46825	5.00E-05	0.000967
LOC100506233	1.46626	0.0014	0.013185
ING1	1.46606	0.00015	0.002431
CRY1	1.46435	0.0001	0.001745
LOC392364	1.46434	0.00465	0.031738
SEPW1	1.46419	5.00E-05	0.000967
LRRC73	1.46308	0.00705	0.043012
CD68	1.46248	0.00015	0.002431
KIAA1683	1.46107	0.00025	0.003659
ADRA2A	1.45994	0.0017	0.015408
LIMA1	1.45861	0.0004	0.005167
SPON1	1.4558	0.00035	0.004678
SIGLEC16	1.45564	0.00035	0.004678
CECR6	1.45551	0.00235	0.019365
SLC25A42	1.45446	0.00065	0.007427
ETV2	1.4543	0.0016	0.014734
ANKRD35	1.45386	0.0043	0.029915
H1FX	1.45371	5.00E-05	0.000967
UFC1	1.45358	0.00025	0.003659
ANKRD20A19P	1.45319	0.0009	0.009427
RPS6KL1	1.45312	5.00E-05	0.000967
KATNA1	1.45276	0.00015	0.002431
HIST1H2AC	1.45167	0.0002	0.003062
KCNS1	1.4515	0.00025	0.003659
GTF2B	1.45118	5.00E-05	0.000967
UTY	1.45005	5.00E-05	0.000967
RASAL2	1.44808	5.00E-05	0.000967
TNC	1.44765	0.00085	0.009052
LILRA6	1.44759	0.00065	0.007427
ATP1B2	1.44718	0.0009	0.009427
LINC00520	1.4469	0.0046	0.0315
BTBD19	1.44627	0.0071	0.043222
ALB	1.44359	0.00835	0.048793
KIF17	1.44291	0.00045	0.005638

NUP210L	1.44037	5.00E-05	0.000967
FYB	1.44021	0.0002	0.003062
PCID2	1.43962	5.00E-05	0.000967
HIST2H2BA	1.43823	0.0007	0.007858
CHMP4C	1.43778	5.00E-05	0.000967
VSIG1	1.43665	0.0005	0.006117
AKR7L	1.43612	0.0005	0.006117
SLC35G5	1.43149	0.0007	0.007858
LOC100130705	1.43101	0.00045	0.005638
ENOX1	1.42977	0.0027	0.02138
SLC27A3	1.42883	0.0004	0.005167
NRXN2	1.42804	0.00295	0.022658
ASPDH	1.42763	0.0026	0.020846
TRIP11	1.42551	5.00E-05	0.000967
NIPBL	1.42537	5.00E-05	0.000967
LRRC34	1.42525	0.0029	0.022408
ITGA11	1.42346	0.0004	0.005167
IGSF1	1.42175	0.00075	0.008257
XAF1	1.4211	0.00145	0.01361
PRDM8	1.42097	0.004	0.02841
EPC1	1.4194	5.00E-05	0.000967
SMURF2	1.41895	5.00E-05	0.000967
FAM46A	1.41839	5.00E-05	0.000967
CHRM4	1.41713	0.00755	0.045412
ASGR1	1.417	0.00145	0.01361
IL5RA	1.41647	0.00375	0.027122
VEGFC	1.41608	0.00025	0.003659
PLAGL1	1.41586	0.0008	0.008653
INSM2	1.41425	0.0051	0.033918
FAM53C	1.41361	5.00E-05	0.000967
CAMK2A	1.41247	0.0026	0.020846
TMEM119	1.41105	0.0051	0.033918
RBBP6	1.40995	0.0003	0.004153
GORAB	1.40972	0.00035	0.004678
CABP4	1.40854	0.00015	0.002431
PNPT1	1.40837	5.00E-05	0.000967
SPOCK1	1.40785	0.00055	0.006573
PTBP2	1.40731	0.00015	0.002431
C1orf52	1.40659	5.00E-05	0.000967
TMCC2	1.4055	0.00075	0.008257
LRRN4	1.4046	0.0009	0.009427
ANKRD33B	1.40353	5.00E-05	0.000967
KLC4	1.4029	0.00065	0.007427
CLK4	1.40278	0.00015	0.002431
MLL5	1.40186	0.0001	0.001745
DKK1	1.40086	0.0001	0.001745
LY6G5C	1.39929	0.0068	0.041918
ZNF335	1.39893	0.00015	0.002431
PON1	1.39817	0.00095	0.009766

HELQ	1.39795	0.0001	0.001745
SPATA21	1.3971	0.0017	0.015408
DNAJB4	1.39704	0.00035	0.004678
LOC100505702	1.39555	0.0013	0.01252
DUSP16	1.39407	5.00E-05	0.000967
GPATCH2L	1.39335	5.00E-05	0.000967
HSPB8	1.39246	0.0001	0.001745
HIST1H2BC	1.39242	5.00E-05	0.000967
PYROXD2	1.39094	0.0014	0.013185
RRN3P1	1.39067	0.0004	0.005167
HIP1R	1.39006	0.0002	0.003062
LPPR3	1.38978	0.0039	0.027879
FAIM3	1.38968	0.00855	0.049544
IFRD1	1.38884	5.00E-05	0.000967
BAZ1A	1.38882	5.00E-05	0.000967
DHRS7B	1.3871	0.00025	0.003659
SEC14L5	1.38629	0.0013	0.01252
TAF2	1.38459	0.00015	0.002431
NFYB	1.38258	0.0009	0.009427
PDE10A	1.38229	0.0001	0.001745
MOK	1.38229	0.0003	0.004153
TP53INP2	1.38157	0.00025	0.003659
RPS2P32	1.38155	0.00055	0.006573
ARF4	1.38113	0.0002	0.003062
TSC22D2	1.38101	5.00E-05	0.000967
IL18R1	1.38064	0.0012	0.011824
CAMK2B	1.38019	0.0019	0.016639
FAM214A	1.37982	0.0004	0.005167
DNAJC27	1.37961	0.0001	0.001745
RGL2	1.37884	0.00025	0.003659
SOCS3	1.37819	5.00E-05	0.000967
C12orf61	1.37423	0.0007	0.007858
LOC285593	1.37367	0.0054	0.035445
LOC646999	1.37298	0.0032	0.024153
SLC39A10	1.37167	0.0001	0.001745
NEDD4	1.37086	5.00E-05	0.000967
NAA38	1.36974	0.00035	0.004678
ALS2CR12	1.36872	0.00415	0.029152
SAMD4A	1.36837	5.00E-05	0.000967
SP2	1.36707	0.00015	0.002431
ZNF385A	1.36466	0.001	0.010186
OR13H1	1.36445	0.0038	0.027341
LOC100506385	1.36391	0.007	0.042786
ZNF528	1.36359	0.00345	0.025496
HELZ	1.36288	5.00E-05	0.000967
NSRP1	1.36241	0.0003	0.004153
SIGLEC11	1.36145	0.0014	0.013185
IRF7	1.36135	0.0009	0.009427
CYP3A4	1.36053	0.00235	0.019365

VWCE	1.35886	0.00215	0.018214
HIST1H1C	1.35837	0.00015	0.002431
GLCCI1	1.35653	0.0005	0.006117
RTL1	1.356	0.0006	0.007015
GP6	1.35598	0.0006	0.007015
NAIP	1.35507	0.0004	0.005167
REEP2	1.35117	0.00245	0.019913
STAT2	1.35115	0.0002	0.003062
C16orf96	1.35093	0.0058	0.037289
EREG	1.34965	0.0004	0.005167
MIRLET7DHG	1.34921	0.0004	0.005167
ZNF554	1.34785	0.0006	0.007015
FOXD4	1.34599	0.00095	0.009766
HAPLN3	1.34589	0.0024	0.019631
KRT15	1.34565	0.00465	0.031738
ANO6	1.34466	0.0001	0.001745
ZC3H12D	1.34421	0.0004	0.005167
AQP7P1	1.3439	0.00225	0.01885
CYP2B7P1	1.3432	0.00155	0.014354
KIAA1217	1.34233	0.00045	0.005638
GOLGA7B	1.34226	0.0014	0.013185
UCP3	1.34057	0.00035	0.004678
LRRIQ1	1.34003	0.00405	0.028668
CCDC144C	1.3374	0.00125	0.012208
PML	1.33705	0.0011	0.011035
TTC33	1.33677	0.0006	0.007015
HAL	1.33433	0.0026	0.020846
KLB	1.33157	0.0006	0.007015
PPP2R3C	1.33097	0.0004	0.005167
AZIN1	1.33006	5.00E-05	0.000967
FAR2	1.32976	0.00265	0.021074
ADAMTS4	1.32899	0.0032	0.024153
ZNF165	1.32849	0.0006	0.007015
FLG	1.32786	0.00095	0.009766
C5AR1	1.32779	0.0003	0.004153
G2E3	1.3259	0.00065	0.007427
ATP6V1D	1.32521	0.00025	0.003659
BEST3	1.32454	0.00295	0.022658
TYW5	1.32425	0.005	0.033413
CD302	1.32409	0.0035	0.025785
HIST1H4L	1.32319	0.00035	0.004678
SGMS2	1.32237	0.0004	0.005167
ZNF274	1.32225	5.00E-05	0.000967
LINC00707	1.32073	0.0018	0.016069
HIST1H3I	1.32065	0.00025	0.003659
ALOX15	1.32056	0.00805	0.047457
DIP2C	1.31916	0.00075	0.008257
ZNF713	1.31815	0.00085	0.009052
SLC5A5	1.31768	0.0019	0.016639

SCG5	1.31735	0.0013	0.01252
PRDM1	1.31734	0.00025	0.003659
FBXO40	1.31567	0.0024	0.019631
TLE4	1.31561	0.00015	0.002431
ZNF821	1.31529	0.0013	0.01252
ANKK1	1.31511	0.00475	0.032248
NR4A2	1.31398	0.0005	0.006117
HAPLN4	1.31367	0.0027	0.02138
SLC4A7	1.31328	0.00035	0.004678
TRIO	1.31206	0.00015	0.002431
KCNJ14	1.31027	0.0002	0.003062
HOXB4	1.30826	0.00085	0.009052
PARP11	1.30765	0.0013	0.01252
IL1R2	1.30741	0.0033	0.024673
OTX2	1.30726	0.00655	0.040923
RAB5A	1.30604	0.0011	0.011035
BEND3P3	1.30501	0.0002	0.003062
CACNB2	1.30428	0.00045	0.005638
C8orf42	1.30384	0.0009	0.009427
CAPN3	1.3032	0.00645	0.040481
PMAIP1	1.3022	5.00E-05	0.000967
ASB4	1.3002	0.0055	0.035846
LOC100506124	1.2997	0.00095	0.009766
ELMSAN1	1.29848	5.00E-05	0.000967
USP16	1.29763	0.0008	0.008653
MCCC1	1.2966	0.00055	0.006573
MITF	1.29602	0.0006	0.007015
RHBDF1	1.29592	0.0007	0.007858
CYFIP1	1.29505	0.0002	0.003062
PLEK2	1.29424	0.00085	0.009052
CEP135	1.29364	0.00095	0.009766
TM9SF3	1.29363	0.00045	0.005638
PNRC1	1.29282	0.0008	0.008653
FHDC1	1.29199	0.00065	0.007427
FSTL4	1.29173	0.0033	0.024673
GPR137B	1.2905	0.0013	0.01252
CCNC	1.28995	0.00085	0.009052
MIR600HG	1.28976	0.0004	0.005167
ALDOC	1.28921	0.002	0.017269
SEMA3C	1.28901	5.00E-05	0.000967
ARHGEF33	1.28836	0.0049	0.032984
RNF10	1.28824	5.00E-05	0.000967
NGFR	1.28802	0.00655	0.040923
ELF1	1.28718	0.00015	0.002431
LANCL2	1.28717	0.00015	0.002431
PADI3	1.28625	0.0017	0.015408
IFNGR2	1.28584	0.00065	0.007427
SMC4	1.28535	0.00045	0.005638
TNFRSF10D	1.2847	0.0001	0.001745

HIST1H2BN	1.28469	0.0011	0.011035
AXL	1.28415	0.00015	0.002431
OPTN	1.28413	0.0005	0.006117
KIAA1614	1.28369	0.0012	0.011824
TJAP1	1.2826	0.0005	0.006117
BCL10	1.28233	0.00055	0.006573
PRKAR1A	1.28046	0.007	0.042786
AP1G1	1.28042	5.00E-05	0.000967
STAT5A	1.27949	0.0009	0.009427
TEX38	1.27913	0.0082	0.048086
NXF1	1.27839	0.0003	0.004153
BIRC2	1.27816	0.0007	0.007858
CLIC2	1.2778	0.00565	0.036623
CYB5D1	1.27728	0.0044	0.030432
EPX	1.27684	0.00415	0.029152
ATP4A	1.27356	0.00275	0.021673
TTN	1.27269	0.00255	0.020565
CLCN4	1.27215	0.0003	0.004153
CD5	1.27166	0.0055	0.035846
SOCS2	1.27054	0.00075	0.008257
DCBLD2	1.26865	0.00025	0.003659
RABGGTB	1.2682	0.0005	0.006117
APOL1	1.26767	0.00085	0.009052
SNX16	1.26758	0.00405	0.028668
VAV1	1.26331	0.00065	0.007427
HCFC2	1.26205	0.00055	0.006573
SSTR2	1.26086	0.00135	0.012838
DNAJB6	1.2588	0.0007	0.007858
USP49	1.25532	0.001	0.010186
FER1L5	1.25524	0.0024	0.019631
TBC1D23	1.2552	0.0006	0.007015
BTG3	1.25492	0.00065	0.007427
CHORDC1	1.25448	0.0007	0.007858
FBXO38	1.25434	0.00025	0.003659
RAB11FIP4	1.25329	0.00135	0.012838
DUSP1	1.25177	0.0004	0.005167
KBTBD8	1.25168	0.0012	0.011824
TRIML2	1.25114	0.00015	0.002431
MAP4K3	1.25097	0.0005	0.006117
C2orf42	1.25063	0.00035	0.004678
FOSL2	1.25021	0.00045	0.005638
NLRC5	1.24936	0.00205	0.017591
UBE2H	1.24857	0.00045	0.005638
C1QTNF7	1.24853	0.00605	0.038539
TNFRSF10B	1.2482	0.0008	0.008653
PRR5L	1.24819	0.0015	0.013976
MYBL1	1.24522	0.00265	0.021074
OSCP1	1.24467	0.0046	0.0315
RCHY1	1.24417	0.00175	0.015698

HSPA1A	1.24225	0.00075	0.008257
LOC100133612	1.24182	0.00395	0.028152
DIABLO	1.23947	0.0007	0.007858
LRRC25	1.2392	0.006	0.038323
GULP1	1.23894	0.0008	0.008653
TMED6	1.23765	0.00415	0.029152
TAS2R3	1.23735	0.0013	0.01252
FBXO36	1.2373	0.0012	0.011824
ARNTL	1.23654	0.00085	0.009052
EMP3	1.23635	0.00065	0.007427
CEP95	1.2361	0.0003	0.004153
LAMB4	1.23481	0.00285	0.022187
HCRTR1	1.23478	0.0034	0.025194
SLC24A1	1.23446	0.00075	0.008257
CCNT2	1.23401	0.0004	0.005167
GTF2H1	1.23386	0.00035	0.004678
HIST2H2AC	1.23269	0.00055	0.006573
METTL15	1.23119	0.0005	0.006117
KRBA2	1.2287	0.0003	0.004153
POU5F1	1.22843	0.00185	0.016374
ZC3H7A	1.22805	0.00155	0.014354
TIMD4	1.22697	0.001	0.010186
INO80D	1.22621	0.0003	0.004153
SLC23A3	1.22338	0.00305	0.023264
CREBBP	1.22333	0.00045	0.005638
RAB30	1.22313	0.00095	0.009766
RBM5	1.22294	0.00065	0.007427
NEU1	1.22086	0.0008	0.008653
MED13	1.22079	0.0003	0.004153
MARCH4	1.21965	0.002	0.017269
MAPK8IP1	1.21945	0.0037	0.026819
SMEK2	1.21929	0.0005	0.006117
CRYBA1	1.21855	0.0051	0.033918
LOC440905	1.21839	0.00315	0.023852
CLDN11	1.21837	0.00515	0.034168
THUMPD2	1.21806	0.00125	0.012208
SUB1	1.21793	0.00195	0.016979
SERPINB9	1.21768	0.0006	0.007015
PLAUR	1.21705	0.00125	0.012208
LOC100506548	1.2162	0.0006	0.007015
BZW1	1.21549	0.00325	0.024387
PILRA	1.21545	0.00305	0.023264
IFT57	1.21466	0.00165	0.015053
TRA2B	1.21451	0.00025	0.003659
MED27	1.21261	0.00175	0.015698
GCNT1	1.21206	0.0017	0.015408
GBF1	1.21203	0.0004	0.005167
SLU7	1.21171	0.00055	0.006573
C10orf118	1.2113	0.001	0.010186

SP100	1.21121	0.0029	0.022408
PPL	1.21089	0.00095	0.009766
FAM65C	1.21058	0.0014	0.013185
LOC283663	1.21033	0.0024	0.019631
MYEF2	1.2098	0.0019	0.016639
ENY2	1.20979	0.00225	0.01885
USP9Y	1.20883	0.00175	0.015698
PRSS27	1.2086	0.00455	0.031261
LOC92249	1.20705	0.0017	0.015408
ZKSCAN1	1.20647	0.00025	0.003659
SEC23B	1.2061	0.0003	0.004153
PPEF1	1.2061	0.00675	0.041733
SLCO1B3	1.20511	0.00295	0.022658
MLL3	1.20501	0.0002	0.003062
FLJ12825	1.20416	0.0081	0.047701
SLC45A3	1.2025	0.0025	0.02025
KDSR	1.20241	0.00065	0.007427
DTX3L	1.20141	0.00415	0.029152
EP300	1.20134	0.00015	0.002431
BCLAF1	1.20013	0.00045	0.005638
SON	1.19939	0.0004	0.005167
CDKN2D	1.19798	0.0034	0.025194
ZMYM5	1.19723	0.00385	0.027605
ZNF350	1.19678	0.00085	0.009052
ARSJ	1.19672	0.00045	0.005638
TTC32	1.19672	0.00215	0.018214
STK11IP	1.19505	0.0019	0.016639
RARRES1	1.19501	0.00535	0.035214
YY1AP1	1.1946	0.00035	0.004678
TRIM31	1.19398	0.0034	0.025194
UBR5	1.1931	0.0003	0.004153
CHMP2B	1.19292	0.0018	0.016069
PIGA	1.19272	0.00105	0.010623
HOMER1	1.19239	0.00135	0.012838
STX5	1.19185	0.00105	0.010623
AMN1	1.19119	0.00405	0.028668
IP6K2	1.19061	0.0007	0.007858
MED30	1.18987	0.002	0.017269
HNRPLL	1.18947	0.00065	0.007427
LIPG	1.18946	0.00615	0.039026
KDM5D	1.18945	0.00105	0.010623
LRRN4CL	1.18849	0.0028	0.021921
DDX3Y	1.18704	0.0011	0.011035
CHRND	1.18646	0.0026	0.020846
LRRC48	1.1862	0.0019	0.016639
MGC16275	1.18604	0.0008	0.008653
SMNDC1	1.18126	0.0024	0.019631
KCNH3	1.18118	0.0048	0.032482
ATAD2B	1.18026	0.0013	0.01252

SAMD8	1.18019	0.0007	0.007858
GRIN2C	1.17979	0.00625	0.03948
ODC1	1.17947	0.0004	0.005167
HNRNPH3	1.17829	0.0035	0.025785
FAM91A1	1.17797	0.00085	0.009052
RNMT	1.17646	0.0005	0.006117
RASA4	1.17557	0.0027	0.02138
MIER1	1.17532	0.00225	0.01885
CATSPER3	1.17413	0.0072	0.043687
AGER	1.17344	0.0037	0.026819
USP43	1.17336	0.00215	0.018214
PIK3C2A	1.17325	0.0014	0.013185
BTAF1	1.1732	0.00095	0.009766
BACH2	1.1722	0.0049	0.032984
SLMAP	1.17142	0.00165	0.015053
ZNF720	1.1711	0.00165	0.015053
SAMD9L	1.17088	0.00705	0.043012
PVRL2	1.16817	0.0018	0.016069
STAC3	1.1679	0.0044	0.030432
ARIH1	1.16734	0.0004	0.005167
MYSM1	1.16705	0.0006	0.007015
HSD17B7P2	1.16688	0.00415	0.029152
GATAD2B	1.16625	0.0004	0.005167
RAB22A	1.16568	0.00115	0.011454
ZFP36L1	1.16463	0.00025	0.003659
SREK1	1.16456	0.0052	0.034431
VEZF1	1.16436	0.0012	0.011824
PTGIS	1.16408	0.0047	0.031987
EXT1	1.16405	0.0009	0.009427
TIGD3	1.16292	0.00485	0.03274
FAM124A	1.16216	0.0026	0.020846
CHGB	1.16179	0.0041	0.028947
ERCC6L2	1.16177	0.0044	0.030432
RB1CC1	1.16171	0.00215	0.018214
FAM22D	1.15982	0.00175	0.015698
NDUFAF5	1.15891	0.0022	0.018543
HIST3H2BB	1.15872	0.00175	0.015698
CRHR2	1.15852	0.0068	0.041918
ITGAX	1.15848	0.0057	0.036875
PAPOLA	1.1584	0.0018	0.016069
PAPD4	1.15665	0.00125	0.012208
KIF20B	1.15632	0.0039	0.027879
ATG4A	1.1557	0.0024	0.019631
GGPS1	1.15542	0.00215	0.018214
FRS2	1.15288	0.00075	0.008257
HSPA1B	1.15279	0.0015	0.013976
VAMP7	1.15155	0.00745	0.044892
RABGEF1	1.15146	0.0021	0.017919
FMR1	1.15129	0.005	0.033413

PPIG	1.15077	0.00225	0.01885
DCUN1D3	1.14848	0.0019	0.016639
AASS	1.14813	0.0056	0.036355
POTEM	1.14676	0.0038	0.027341
FUNDC2	1.14552	0.00175	0.015698
ETF1	1.14524	0.00085	0.009052
MAP4K5	1.14511	0.0016	0.014734
UBR1	1.14505	0.0011	0.011035
TMEM67	1.14327	0.00585	0.037553
CWC22	1.14259	0.0016	0.014734
ZBTB17	1.14253	0.0021	0.017919
NUPL1	1.1421	0.0006	0.007015
KLF2	1.13986	0.0037	0.026819
SNAP23	1.13927	0.0034	0.025194
WAPAL	1.1388	0.00135	0.012838
TMCO3	1.13838	0.00185	0.016374
DGUOK	1.13778	0.001	0.010186
TM9SF4	1.13443	0.00135	0.012838
AFF4	1.13424	0.00095	0.009766
RYBP	1.13359	0.00275	0.021673
FBXO33	1.13317	0.00135	0.012838
SPATA2	1.13201	0.00155	0.014354
ADPRM	1.13189	0.003	0.022946
GPR111	1.13043	0.00655	0.040923
ACSL5	1.12948	0.00715	0.043478
PRPF38B	1.1287	0.0015	0.013976
SIM2	1.12812	0.00235	0.019365
ACSL4	1.12736	0.0033	0.024673
ZYX	1.12586	0.00425	0.029666
CAB39L	1.12517	0.00475	0.032248
C3	1.12483	0.00315	0.023852
BTN3A1	1.12429	0.0029	0.022408
ANKRD42	1.12382	0.00325	0.024387
HCCS	1.12253	0.0013	0.01252
PRICKLE1	1.12253	0.0037	0.026819
USP9X	1.1225	0.0017	0.015408
NT5E	1.12198	0.00225	0.01885
MAPT	1.12166	0.00455	0.031261
AHR	1.12091	0.00125	0.012208
TANK	1.12071	0.0065	0.040703
TRIM5	1.12034	0.00335	0.024946
GDAP1	1.11999	0.0027	0.02138
SCFD1	1.11955	0.0034	0.025194
CATSPERG	1.11893	0.0062	0.039223
CREB1	1.11787	0.0006	0.007015
B3GNT5	1.11763	0.005	0.033413
BLZF1	1.11759	0.0036	0.026323
TMEM107	1.11749	0.0019	0.016639
LOC338817	1.11733	0.00685	0.042163

USP18	1.1171	0.00265	0.021074
AFTPH	1.11707	0.0022	0.018543
NR2E3	1.11517	0.0057	0.036875
SLC9A8	1.11423	0.00125	0.012208
TRAPPC6B	1.11377	0.0037	0.026819
TRIB1	1.1128	0.0011	0.011035
ATF7IP2	1.11252	0.0043	0.029915
AKAP8L	1.11107	0.00175	0.015698
ITSN1	1.10918	0.0028	0.021921
TAPT1	1.10889	0.00245	0.019913
RGS20	1.10187	0.0029	0.022408
SYF2	1.10024	0.00455	0.031261
SAV1	1.10007	0.0025	0.02025
PSMC6	1.09967	0.00235	0.019365
DPM1	1.09911	0.0026	0.020846
SLC39A9	1.09893	0.00295	0.022658
CSRP2	1.09867	0.0073	0.044132
CRB2	1.09836	0.0048	0.032482
TGFB2	1.09756	0.00175	0.015698
PPIL4	1.0975	0.00515	0.034168
STAR	1.09738	0.0042	0.029379
TAB3	1.09649	0.0029	0.022408
CWC25	1.09643	0.00095	0.009766
SLC35F3	1.09584	0.00325	0.024387
GPCPD1	1.0952	0.00175	0.015698
BRWD3	1.09356	0.00325	0.024387
YAF2	1.09352	0.0032	0.024153
CLMP	1.09172	0.0039	0.027879
MSS51	1.09068	0.0043	0.029915
GGN	1.08915	0.0068	0.041918
ZFAT	1.08832	0.0039	0.027879
TTC27	1.08816	0.00135	0.012838
TBK1	1.08799	0.00215	0.018214
NBR1	1.08723	0.00135	0.012838
CBWD1	1.08617	0.00445	0.030676
MICALL2	1.086	0.0076	0.045548
CASP10	1.08459	0.00605	0.038539
ARHGAP31	1.08423	0.003	0.022946
MRPL47	1.08391	0.0067	0.041533
LRIF1	1.08376	0.0055	0.035846
SAR1A	1.08308	0.0012	0.011824
FAM200B	1.08208	0.0076	0.045548
METTL21D	1.08165	0.00495	0.033213
LOC284581	1.08118	0.008	0.047213
HECA	1.08083	0.00315	0.023852
CHIC2	1.08027	0.00855	0.049544
ALK	1.08022	0.0055	0.035846
USP47	1.07986	0.00185	0.016374
IFIT5	1.07932	0.0021	0.017919

FAM126B	1.07924	0.00285	0.022187
IMPA1	1.07831	0.0062	0.039223
VEZT	1.07797	0.00205	0.017591
BHLHE40	1.07762	0.0018	0.016069
MAK	1.07744	0.0082	0.048086
HNRPDL	1.07742	0.0038	0.027341
SIRT1	1.07725	0.0063	0.039735
RFX3	1.07718	0.00245	0.019913
MTRF1L	1.07681	0.0017	0.015408
TRIM21	1.07592	0.0027	0.02138
RAP1B	1.07481	0.006	0.038323
IRAK2	1.07274	0.00145	0.01361
YOD1	1.07165	0.0027	0.02138
CIC	1.07042	0.0038	0.027341
WTAP	1.07023	0.00135	0.012838
DPY19L2P2	1.06929	0.0036	0.026323
HIST2H2AA3	1.06923	0.00285	0.022187
SBDSP1	1.06905	0.0053	0.034982
SH3GLB1	1.06873	0.0037	0.026819
GMEB1	1.06862	0.0029	0.022408
FAM83G	1.06773	0.00365	0.026607
HIST1H2AJ	1.06738	0.00345	0.025496
MCTS1	1.06709	0.00305	0.023264
POLB	1.06608	0.00285	0.022187
NTN4	1.06603	0.00205	0.017591
CAND2	1.06536	0.003	0.022946
LINC00472	1.06514	0.00285	0.022187
ARL13B	1.06392	0.0053	0.034982
LOC100288637	1.06386	0.00275	0.021673
SPRY4	1.06368	0.00255	0.020565
ST5	1.06331	0.006	0.038323
UPP1	1.06326	0.0022	0.018543
WAC	1.06261	0.0018	0.016069
HAP1	1.0621	0.00795	0.046985
VPS37B	1.05942	0.00255	0.020565
RLF	1.0592	0.0016	0.014734
HIST1H1D	1.05909	0.0028	0.021921
HIST1H3E	1.05841	0.00395	0.028152
GPRC5A	1.05777	0.0019	0.016639
UBA6	1.05764	0.0058	0.037289
UBE2D3	1.05495	0.0023	0.019134
CNOT4	1.05422	0.00275	0.021673
PIK3C3	1.0536	0.00645	0.040481
MITD1	1.05284	0.0055	0.035846
HIST2H4A	1.05283	0.00445	0.030676
LDLR	1.05132	0.0023	0.019134
JMJD6	1.05112	0.0081	0.047701
PRKG2	1.04959	0.00835	0.048793
ARL8B	1.04913	0.00465	0.031738

GPR126	1.04846	0.0027	0.02138
DDX5	1.04781	0.00185	0.016374
RBM24	1.04652	0.00455	0.031261
NMT2	1.04545	0.0038	0.027341
PNISR	1.04461	0.00265	0.021074
BRCC3	1.04185	0.0039	0.027879
UGCG	1.04117	0.00425	0.029666
CDKN2AIP	1.04101	0.00225	0.01885
NPC1	1.04095	0.0035	0.025785
PATL1	1.04064	0.00235	0.019365
ZC3H6	1.04029	0.0062	0.039223
COQ7	1.04002	0.0046	0.0315
COL7A1	1.03985	0.0033	0.024673
CSNK2A2	1.03982	0.00495	0.033213
HIST1H2BM	1.03975	0.0036	0.026323
ATP13A3	1.03914	0.00315	0.023852
PPP1R2	1.03879	0.0082	0.048086
SPIB	1.03877	0.0068	0.041918
GATA6	1.03789	0.0062	0.039223
ZNF461	1.03624	0.00435	0.030187
TTF1	1.03501	0.004	0.02841
ZBTB43	1.03449	0.00265	0.021074
EGFR	1.03387	0.00365	0.026607
ZC3H15	1.03363	0.0053	0.034982
TOR1AIP2	1.03191	0.00165	0.015053
PPP2CB	1.03152	0.00285	0.022187
MTMR3	1.03016	0.00295	0.022658
DRAP1	1.02989	0.0063	0.039735
ADCY7	1.02982	0.0036	0.026323
UBXN2B	1.02911	0.00275	0.021673
ARID2	1.02867	0.0029	0.022408
SRSF6	1.02835	0.0022	0.018543
WNT4	1.02807	0.00535	0.035214
ATG12	1.02545	0.0051	0.033918
SRSF3	1.02318	0.0017	0.015408
EML6	1.02234	0.0066	0.041051
TMCO1	1.02165	0.00635	0.03999
ZC3H12C	1.02087	0.00255	0.020565
RAD18	1.01935	0.0043	0.029915
ANXA3	1.01704	0.0058	0.037289
ZNF764	1.01655	0.00485	0.03274
KIN	1.01606	0.00605	0.038539
STAT1	1.01144	0.0043	0.029915
PTPN21	1.01137	0.0032	0.024153
HIVEP1	1.00982	0.0023	0.019134
ZUFSP	1.00924	0.00695	0.04259
LMO7	1.00679	0.00285	0.022187
SNX9	1.00678	0.0026	0.020846
SBDS	1.00674	0.0051	0.033918

DHX36	1.00639	0.0084	0.048965
ANKRD13A	1.0052	0.0055	0.035846
FLCN	1.00439	0.0064	0.040213
ZNF669	1.00273	0.00545	0.035716
GPATCH8	1.00122	0.00185	0.016374
GCA	1.00015	0.00735	0.04437
CA8	0.999653	0.00575	0.037111
ANKRD13C	0.999599	0.00575	0.037111
SPPL3	0.999193	0.00555	0.036115
IER3	0.998504	0.00325	0.024387
ARID3B	0.997159	0.00515	0.034168
WNK4	0.996955	0.00695	0.04259
AREG	0.996612	0.00435	0.030187
BAZ2B	0.99564	0.00285	0.022187
FNTA	0.995051	0.0066	0.041051
COPS5	0.99401	0.0048	0.032482
PPP4R1L	0.993533	0.0058	0.037289
COQ10B	0.993201	0.00555	0.036115
TMEM81	0.992325	0.00725	0.043926
RBM33	0.989059	0.00295	0.022658
MEF2D	0.988019	0.0038	0.027341
HIST2H2AA4	0.987559	0.0048	0.032482
MIR143HG	0.987404	0.0084	0.048965
PPAP2B	0.987311	0.00415	0.029152
PRKAA2	0.987073	0.00795	0.046985
TEAD1	0.985675	0.0028	0.021921
NF1	0.985217	0.0086	0.049747
SRSF4	0.985091	0.0055	0.035846
ACOT9	0.984385	0.005	0.033413
SMARCA5	0.983197	0.0044	0.030432
WSB1	0.98278	0.00485	0.03274
PPP2CA	0.982607	0.00455	0.031261
HIST1H2AM	0.982296	0.0055	0.035846
CELSR3	0.979975	0.00615	0.039026
C11orf30	0.97948	0.00585	0.037553
BTBD1	0.97939	0.0048	0.032482
SLC12A7	0.978048	0.0082	0.048086
UBN2	0.974927	0.00455	0.031261
CNOT2	0.974613	0.005	0.033413
SH3KBP1	0.972277	0.00475	0.032248
ZMYM4	0.970257	0.00435	0.030187
ZNF638	0.967601	0.00575	0.037111
PPID	0.965831	0.0079	0.046806
TET2	0.96583	0.00415	0.029152
HIST1H2BO	0.96526	0.0066	0.041051
AGAP6	0.964724	0.00535	0.035214
ALKBH1	0.964316	0.00565	0.036623
ATF7IP	0.963908	0.00435	0.030187
SPOP	0.962137	0.006	0.038323

SAP30BP	0.960364	0.0052	0.034431
MYO5B	0.960266	0.0072	0.043687
HAUS2	0.959926	0.00675	0.041733
NOC3L	0.95906	0.00625	0.03948
ARIH2	0.957053	0.00665	0.041284
RBFOX2	0.956086	0.0036	0.026323
C9orf131	0.95558	0.00745	0.044892
HIST1H4B	0.954704	0.00595	0.038106
USP8	0.953026	0.00725	0.043926
KIAA0907	0.952523	0.0046	0.0315
BDP1	0.951526	0.00755	0.045412
CCDC117	0.950715	0.00675	0.041733
SNIP1	0.949373	0.0054	0.035445
EFTUD1	0.948999	0.0056	0.036355
NR3C1	0.94744	0.0057	0.036875
ATF1	0.946907	0.00745	0.044892
CHD3	0.94666	0.005	0.033413
FXR1	0.946465	0.0084	0.048965
C1orf63	0.945909	0.00765	0.045782
MON2	0.944043	0.00815	0.047894
PSME4	0.943479	0.0053	0.034982
APOL6	0.939711	0.00785	0.046626
FRMD4B	0.939433	0.008	0.047213
PPP6R3	0.935615	0.00675	0.041733
PNN	0.933958	0.0056	0.036355
EYA3	0.933235	0.0062	0.039223
WDR37	0.932131	0.0064	0.040213
HNRNPC	0.931612	0.0055	0.035846
GOSR1	0.930167	0.0046	0.0315
RLIM	0.930104	0.00605	0.038539
SMU1	0.924983	0.00565	0.036623
RC3H2	0.923355	0.0055	0.035846
SVIL	0.916683	0.0078	0.046428
SRGAP1	0.914578	0.0054	0.035445
TERF2	0.914278	0.0086	0.049746
RC3H1	0.91377	0.00555	0.036115
PRPF38A	0.910531	0.00825	0.048311
KDM5C	0.90986	0.0083	0.048535
WBP11	0.908915	0.0061	0.038798
DDX39B	0.904371	0.00745	0.044892
PTPRK	0.901918	0.0086	0.049747
HSPA4	0.898982	0.00845	0.049135
RPPH1	0.894542	0.0085	0.04934
DNAJA1	0.882594	0.0083	0.048535
EAF1	0.868613	0.00825	0.048311
TRIM25	0.868006	0.0076	0.045548
POGZ	0.857348	0.008	0.047213
HIPK3	0.851921	0.00845	0.049135
MLEC	-0.87263	0.00845	0.049135

NUDT19	-0.88365	0.00775	0.046247
SEC61A1	-0.88509	0.008	0.047213
UCK2	-0.89194	0.00855	0.049544
RCC2	-0.90612	0.0076	0.045548
NT5DC3	-0.90984	0.0077	0.046014
OXA1L	-0.91209	0.00805	0.047457
C17orf51	-0.91735	0.00605	0.038539
DKC1	-0.92349	0.0062	0.039223
SLC35F6	-0.92547	0.007	0.042786
TGIF1	-0.92722	0.0064	0.040213
TBL1X	-0.92929	0.00785	0.046626
EDARADD	-0.93243	0.00855	0.049544
NOP56	-0.9326	0.0064	0.040213
ERMP1	-0.93268	0.0076	0.045548
LRRC37A4P	-0.93418	0.0068	0.041918
LONRF2	-0.93434	0.00615	0.039026
ALDH3A2	-0.94029	0.00445	0.030676
CYB561	-0.94179	0.0085	0.04934
NUP210	-0.94503	0.0082	0.048086
LASP1	-0.94657	0.00655	0.040923
PABPC1L	-0.94811	0.00785	0.046626
ECH1	-0.95378	0.00765	0.045782
ZNF584	-0.95407	0.00695	0.04259
RBL1	-0.95523	0.0067	0.041533
MARS2	-0.95576	0.0071	0.043222
ZADH2	-0.95677	0.00855	0.049544
SFXN4	-0.9572	0.0085	0.04934
UCHL1	-0.96037	0.00325	0.024387
RETSAT	-0.96506	0.00595	0.038106
RIN2	-0.96635	0.00425	0.029666
ID2	-0.9665	0.00315	0.023852
TEX2	-0.96697	0.00445	0.030676
RRP1B	-0.96781	0.00535	0.035214
PLEKHA2	-0.96809	0.00755	0.045412
ORMDL3	-0.96827	0.0066	0.041051
SLC45A4	-0.96833	0.00775	0.046247
AMIGO2	-0.96841	0.00245	0.019913
CNNM1	-0.96933	0.0042	0.029379
TRUB2	-0.96952	0.00685	0.042163
ADO	-0.9714	0.00455	0.031261
GAPDH	-0.97192	0.0062	0.039223
DPM2	-0.97385	0.00815	0.047894
TRMT61A	-0.97452	0.0073	0.044132
TDP2	-0.97482	0.0048	0.032482
SRGN	-0.97605	0.00815	0.047894
PRRC1	-0.97817	0.0051	0.033918
SSR3	-0.98047	0.0057	0.036875
SLC9A2	-0.98075	0.00815	0.047894
ZNF70	-0.98095	0.0076	0.045548

ZNF114	-0.98123	0.0071	0.043222
MRGBP	-0.98208	0.00705	0.043012
UHRF1	-0.98227	0.0066	0.041051
MMP24	-0.98265	0.0079	0.046806
DNAJC30	-0.98375	0.00775	0.046247
NCAPD3	-0.98422	0.0046	0.0315
PDIA6	-0.98537	0.007	0.042786
NABP2	-0.98653	0.0049	0.032984
ONECUT2	-0.98666	0.0077	0.046014
ZNF367	-0.98734	0.005	0.033413
TBC1D4	-0.98758	0.0054	0.035445
TCP11L1	-0.98966	0.00575	0.037111
SYNM	-0.99039	0.003	0.022946
TRIM27	-0.99141	0.00355	0.026073
STAG3L2	-0.9919	0.0071	0.043222
FAM220A	-0.99277	0.00435	0.030187
RASSF3	-0.99377	0.00345	0.025496
ZFP64	-0.99533	0.007	0.042786
SLC7A5P2	-0.99849	0.0079	0.046806
TIMELESS	-1.00042	0.0036	0.026323
TSEN2	-1.00208	0.00665	0.041284
ZBTB9	-1.00231	0.0055	0.035846
C7orf25	-1.00231	0.0073	0.044132
PLEKHH2	-1.00321	0.0078	0.046428
XPOT	-1.00374	0.00405	0.028668
GEMIN6	-1.0051	0.0079	0.046806
FLRT3	-1.00548	0.0078	0.046428
DDR1	-1.00846	0.0084	0.048965
TET1	-1.00943	0.006	0.038323
LOC728554	-1.00977	0.0076	0.045548
SEPHS2	-1.01044	0.00315	0.023852
ZNF768	-1.01076	0.00525	0.034748
TCF19	-1.01208	0.00705	0.043012
TRAPPC1	-1.0139	0.0059	0.037844
DYRK2	-1.01451	0.00335	0.024946
LGR4	-1.01471	0.0033	0.024673
MCM10	-1.01509	0.0038	0.027341
KCTD1	-1.01581	0.00465	0.031738
PDDC1	-1.01608	0.00575	0.037111
EPS8L2	-1.01639	0.0071	0.043222
AVPI1	-1.01801	0.0042	0.029379
KBTBD11	-1.01993	0.00495	0.033213
ZNF598	-1.02026	0.00665	0.041284
RAPGEF5	-1.02035	0.0067	0.041533
MKI67	-1.02063	0.0021	0.017919
EMP2	-1.02145	0.00415	0.029152
CALM3	-1.02305	0.00325	0.024387
NCS1	-1.0232	0.00545	0.035716
LPAR1	-1.02413	0.00375	0.027122

AES	-1.02418	0.0072	0.043687
MRPL17	-1.02488	0.00375	0.027122
SCARA3	-1.02503	0.00785	0.046626
STAG3L1	-1.02831	0.00495	0.033213
PCLO	-1.02834	0.00795	0.046985
DUSP18	-1.0285	0.00775	0.046247
FKBP9	-1.02853	0.0037	0.026819
SLC25A23	-1.02981	0.0083	0.048535
NAGLU	-1.02994	0.0055	0.035846
NLN	-1.03099	0.00425	0.029666
PBK	-1.03163	0.00845	0.049135
ZNF740	-1.03186	0.00445	0.030676
FBXW5	-1.03436	0.006	0.038323
VANGL1	-1.0345	0.00355	0.026073
GIT1	-1.03571	0.00385	0.027605
GINS4	-1.03807	0.00385	0.027605
ALDH1A3	-1.039	0.00325	0.024387
UBE2T	-1.03946	0.00475	0.032248
APEX2	-1.03965	0.00575	0.037111
MRPS26	-1.04107	0.00385	0.027605
APEX1	-1.04175	0.00175	0.015698
C5orf54	-1.04278	0.00805	0.047457
PRPSAP1	-1.04343	0.0056	0.036355
INTS5	-1.04358	0.0029	0.022408
RRS1	-1.04425	0.00445	0.030676
TMEM203	-1.04537	0.0035	0.025785
FAM201A	-1.04548	0.0065	0.040703
BLVRB	-1.04611	0.00355	0.026073
ZNF589	-1.04685	0.00465	0.031738
MALL	-1.04731	0.0041	0.028947
KLF12	-1.04768	0.0049	0.032984
CDCA5	-1.0477	0.0022	0.018543
ZNF514	-1.04782	0.00405	0.028668
ARHGEF2	-1.04807	0.00255	0.020565
AKR1B15	-1.04825	0.0027	0.02138
WDR54	-1.04896	0.00695	0.04259
FTL	-1.04899	0.00255	0.020565
KATNAL1	-1.04926	0.0064	0.040213
FAM83H	-1.05196	0.0041	0.028947
FAM219B	-1.05234	0.00165	0.015053
H2AFX	-1.05238	0.0042	0.029379
CHST10	-1.05285	0.0036	0.026323
ADAMTS10	-1.05458	0.00605	0.038539
DNAJC22	-1.05581	0.0038	0.027341
LOC643401	-1.05641	0.0052	0.034431
ALDH3B1	-1.05743	0.0046	0.0315
CYP24A1	-1.05744	0.00155	0.014354
NUAK1	-1.05885	0.00155	0.014354
KIAA0101	-1.05938	0.005	0.033413

SHARPIN	-1.05984	0.00655	0.040923
KIAA1161	-1.05996	0.0054	0.035445
MEX3A	-1.06018	0.0061	0.038798
CTSC	-1.06127	0.0081	0.047701
TRADD	-1.06273	0.0082	0.048086
ALKBH2	-1.06277	0.00465	0.031738
ENDOD1	-1.064	0.0047	0.031987
CAMK2N1	-1.06428	0.00735	0.04437
HECTD3	-1.06455	0.00695	0.04259
CMTM7	-1.06493	0.00715	0.043478
TADA2A	-1.06543	0.00385	0.027605
CDC7	-1.06567	0.0055	0.035846
KIAA0922	-1.06677	0.00495	0.033213
TNS1	-1.06735	0.00735	0.04437
GIPC1	-1.06859	0.00615	0.039026
GNB3	-1.06868	0.00725	0.043926
DAG1	-1.07011	0.0028	0.021921
FOXC1	-1.0708	0.0024	0.019631
LIPA	-1.07094	0.0047	0.031987
MAN1B1	-1.07145	0.00415	0.029152
KCTD12	-1.07183	0.004	0.02841
SAMD5	-1.07187	0.0043	0.029915
POF1B	-1.07391	0.0065	0.040703
BBS2	-1.07405	0.0044	0.030432
GPS1	-1.07415	0.00385	0.027605
MRM1	-1.07439	0.00445	0.030676
TRIM9	-1.07475	0.00245	0.019913
E2F7	-1.07487	0.0024	0.019631
KTN1-AS1	-1.075	0.00635	0.03999
PALB2	-1.07522	0.00225	0.01885
LBR	-1.07574	0.00245	0.019913
CERCAM	-1.07574	0.00865	0.049984
ORC1	-1.07592	0.00295	0.022658
CD24	-1.07716	0.00265	0.021074
ZNF319	-1.07917	0.00365	0.026607
CDT1	-1.07948	0.004	0.02841
FECH	-1.07963	0.0023	0.019134
DGKQ	-1.08111	0.00845	0.049135
EMR1	-1.082	0.00845	0.049135
GMNN	-1.0833	0.005	0.033413
TERT	-1.08336	0.0053	0.034982
EPB49	-1.08395	0.0049	0.032984
MPI	-1.08423	0.003	0.022946
PFKFB3	-1.08458	0.00335	0.024946
ZNF485	-1.08489	0.00765	0.045782
KRBA1	-1.08538	0.0078	0.046428
DHODH	-1.08614	0.00285	0.022187
ZNF691	-1.08721	0.004	0.02841
ACPL2	-1.08731	0.00545	0.035716

CDC42EP4	-1.08732	0.00215	0.018214
ZNF239	-1.08761	0.00695	0.04259
MSH2	-1.08841	0.00395	0.028152
PTPRU	-1.08996	0.0061	0.038798
OLFML2A	-1.09091	0.0043	0.029915
GLRX	-1.09142	0.0058	0.037289
ZNF607	-1.09152	0.00235	0.019365
SLC35G1	-1.0926	0.00715	0.043478
MTUS1	-1.09281	0.0015	0.013976
ZNF823	-1.09443	0.0044	0.030432
RBM43	-1.09509	0.0035	0.025785
TMEM9	-1.09547	0.0049	0.032984
IGSF11	-1.09678	0.00335	0.024946
CDCA4	-1.09749	0.0016	0.014734
DSN1	-1.09776	0.0029	0.022408
EDEM2	-1.09914	0.00305	0.023264
CBX7	-1.09995	0.0069	0.042424
NCR3LG1	-1.10151	0.0024	0.019631
CXXC4	-1.1017	0.0042	0.029379
GTF2IRD1	-1.10205	0.0046	0.0315
GCLC	-1.10211	0.003	0.022946
POR	-1.10241	0.0053	0.034982
PTGFRN	-1.10337	0.00185	0.016374
TPI1	-1.10394	0.0019	0.016639
KDELC2	-1.1045	0.00295	0.022658
SLFN11	-1.10465	0.00375	0.027122
ZNF285	-1.10551	0.00495	0.033213
LEPREL4	-1.10596	0.00305	0.023264
HNF1B	-1.10772	0.0021	0.017919
KLF11	-1.10911	0.0022	0.018543
RNF222	-1.10967	0.0076	0.045548
GSTP1	-1.10973	0.0013	0.01252
MAZ	-1.1102	0.00235	0.019365
TICRR	-1.11047	0.0023	0.019134
HLTF	-1.11146	0.0026	0.020846
FOXN3	-1.11509	0.0044	0.030432
RASSF2	-1.11551	0.002	0.017269
RPP40	-1.11609	0.00475	0.032248
CHST14	-1.1162	0.0084	0.048965
ZNF71	-1.11701	0.0025	0.02025
MCM3	-1.11797	0.00115	0.011454
ANK3	-1.11917	0.0069	0.042424
PLCXD3	-1.11923	0.00545	0.035716
DNASE2	-1.12092	0.0021	0.017919
PXMP4	-1.12093	0.00285	0.022187
FADS1	-1.1216	0.00065	0.007427
ADPRH	-1.12174	0.0058	0.037289
WDR81	-1.12206	0.00235	0.019365
MGST1	-1.12261	0.0024	0.019631

HOXB7	-1.12293	0.00315	0.023852
GALT	-1.12294	0.00455	0.031261
EEF2K	-1.1236	0.0014	0.013185
VWA1	-1.12384	0.0069	0.042424
PSMG3	-1.12387	0.0044	0.030432
ARSE	-1.12477	0.0028	0.021921
RNASEL	-1.12514	0.00275	0.021673
CBX8	-1.12555	0.0037	0.026819
DHRS4L2	-1.12632	0.0065	0.040703
SORD	-1.12639	0.00135	0.012838
CTPS1	-1.12689	0.0004	0.005167
FOXE1	-1.12707	0.00195	0.016979
PHF17	-1.12728	0.0012	0.011824
GPRIN3	-1.12773	0.0007	0.007858
JDP2	-1.12784	0.003	0.022946
CCND1	-1.12799	0.00085	0.009052
RASSF6	-1.12856	0.0037	0.026819
ARRDC2	-1.12861	0.00235	0.019365
UGDH	-1.12899	0.00095	0.009766
RHOBTB3	-1.12968	0.0021	0.017919
PRIM1	-1.13108	0.00265	0.021074
VOPP1	-1.1311	0.0016	0.014734
LOC344887	-1.13122	0.0009	0.009427
FAM155B	-1.13159	0.0029	0.022408
MAPK11	-1.1316	0.0063	0.039735
LRP8	-1.13201	0.0034	0.025194
CBX6	-1.13358	0.0019	0.016639
METRN	-1.13441	0.00595	0.038106
HEATR3	-1.13501	0.00195	0.016979
TMEM14A	-1.13532	0.0066	0.041051
EPHX2	-1.13734	0.00385	0.027605
PLD6	-1.13777	0.0013	0.01252
IGFBP3	-1.1378	0.0015	0.013976
LOC339803	-1.13813	0.0028	0.021921
XBP1	-1.1411	0.00085	0.009052
MAT2A	-1.14192	0.00215	0.018214
PARS2	-1.14213	0.0029	0.022408
LARP1	-1.14232	0.0006	0.007015
MAD2L1	-1.1424	0.0023	0.019134
EVPL	-1.14298	0.0031	0.023602
ANGEL1	-1.14403	0.00175	0.015698
VASH1	-1.14408	0.00275	0.021673
ANXA2R	-1.14455	0.0041	0.028947
SEC16B	-1.14505	0.0047	0.031987
ZNF221	-1.14513	0.00735	0.04437
STX1B	-1.14791	0.003	0.022946
METTL21B	-1.1484	0.00235	0.019365
GATA2	-1.14844	0.00395	0.028152
BAMBI	-1.14887	0.00165	0.015053

FN3KRP	-1.14982	0.00205	0.017591
ACSS1	-1.15085	0.0036	0.026323
FAM129A	-1.15086	0.0023	0.019134
AGPAT2	-1.15093	0.00725	0.043926
DTX4	-1.15117	0.0034	0.025194
STAG3L3	-1.15259	0.00255	0.020565
FAM109A	-1.15513	0.0072	0.043687
LTBR	-1.15531	0.0017	0.015408
CHST3	-1.15559	0.00055	0.006573
USP27X	-1.15577	0.0034	0.025194
ANKRD39	-1.15645	0.00485	0.03274
RALGDS	-1.15675	0.0027	0.02138
VPS26B	-1.15711	0.0016	0.014734
PCOLCE2	-1.1574	0.00415	0.029152
UPK1B	-1.15747	0.002	0.017269
ELF3	-1.15754	0.0012	0.011824
HOXA13	-1.15815	0.0065	0.040703
LRFN1	-1.15827	0.00825	0.048311
ERCC2	-1.15858	0.0078	0.046428
MCM4	-1.15866	0.00075	0.008257
C11orf95	-1.15987	0.0011	0.011035
GAB2	-1.16045	0.0015	0.013976
MCM6	-1.16076	0.00045	0.005638
CEP19	-1.16112	0.00355	0.026073
LINC00482	-1.16199	0.00365	0.026607
PACSIN3	-1.16218	0.00505	0.033693
SLC25A15	-1.1635	0.00515	0.034168
TP53INP1	-1.16369	0.00355	0.026073
CYB5A	-1.164	0.0029	0.022408
MED22	-1.16534	0.00155	0.014354
ZNF658	-1.16567	0.0024	0.019631
DIXDC1	-1.16668	0.00405	0.028668
LMNB2	-1.16684	0.00165	0.015053
PDXP	-1.16704	0.0037	0.026819
TMEM19	-1.16729	0.00185	0.016374
MON1A	-1.16746	0.007	0.042786
PLCH1	-1.1676	0.002	0.017269
KIF3C	-1.16906	0.00145	0.01361
PCK2	-1.16938	0.00185	0.016374
PPCDC	-1.16946	0.00195	0.016979
ZNF469	-1.17028	0.0019	0.016639
SOGA1	-1.17081	0.00205	0.017591
GLT25D1	-1.17112	0.0015	0.013976
FLYWCH2	-1.17123	0.00355	0.026073
SCARB1	-1.17217	0.0018	0.016069
ZNF280B	-1.17219	0.00185	0.016374
ZDHHC23	-1.17224	0.0022	0.018543
TMEM109	-1.17245	0.0025	0.02025
MAVS	-1.17285	0.001	0.010186

HEG1	-1.17356	0.0019	0.016639
KLHDC8B	-1.17577	0.0059	0.037844
ZNF500	-1.1759	0.0022	0.018543
LOC728743	-1.178	0.00555	0.036115
PIR	-1.18055	0.00495	0.033213
KCNG1	-1.18119	0.0052	0.034431
MIIP	-1.18163	0.00665	0.041284
CCDC115	-1.18166	0.0023	0.019134
FOXL1	-1.18311	0.0066	0.041051
SPIN3	-1.18418	0.0066	0.041051
PROCR	-1.18443	0.00105	0.010623
CREB3L2	-1.18522	0.00475	0.032248
TMEM106C	-1.18524	0.00135	0.012838
WFS1	-1.18643	0.0024	0.019631
KRT83	-1.18658	0.0078	0.046428
NRCAM	-1.18776	0.0004	0.005167
CCL2	-1.18804	0.00075	0.008257
SH3GLB2	-1.18827	0.00325	0.024387
MYLK	-1.18912	0.00705	0.043012
SNX18	-1.18932	0.00245	0.019913
SMAD6	-1.19012	0.00565	0.036623
BPHL	-1.19025	0.00275	0.021673
PRDX1	-1.19103	0.00045	0.005638
TALDO1	-1.19174	0.00065	0.007427
KANK2	-1.19256	0.00135	0.012838
CDC25B	-1.19309	0.0008	0.008653
PAX9	-1.19312	0.0023	0.019134
LY6E	-1.19338	0.0021	0.017919
APEH	-1.19377	0.00125	0.012208
SDPR	-1.19457	0.00085	0.009052
MAPK12	-1.19487	0.0038	0.027341
TMTC4	-1.19494	0.0012	0.011824
ASB13	-1.19587	0.00185	0.016374
GBX2	-1.19595	0.0075	0.045177
PCED1B	-1.1965	0.00445	0.030676
AKR1A1	-1.19654	0.0012	0.011824
FAM136A	-1.19694	0.0006	0.007015
SCARA5	-1.19813	0.00265	0.021074
B3GAT3	-1.19875	0.0022	0.018543
RSAD1	-1.19943	0.0007	0.007858
SPSB3	-1.20006	0.0031	0.023602
RIMS3	-1.20104	0.00185	0.016374
FOXA2	-1.20125	0.00115	0.011454
RHOBTB1	-1.20144	0.00075	0.008257
CREG1	-1.20297	0.00135	0.012838
FOXRED2	-1.20365	0.00075	0.008257
ZNF395	-1.20373	0.0007	0.007858
TMEM129	-1.20518	0.00235	0.019365
ANKRD36B	-1.20573	0.00105	0.010623

SLC19A1	-1.20576	0.0055	0.035846
FAM57A	-1.2068	0.00055	0.006573
LINC00488	-1.20716	0.0079	0.046806
SLC6A6	-1.20719	0.004	0.02841
RMI1	-1.20802	0.00245	0.019913
FUT1	-1.2082	0.0025	0.02025
SLC29A1	-1.20878	0.0014	0.013185
PYCRL	-1.20924	0.00235	0.019365
ZNF618	-1.21021	0.00045	0.005638
DGCR6L	-1.21068	0.00645	0.040481
ANKRD36	-1.21127	0.0009	0.009427
MBOAT1	-1.21129	0.0014	0.013185
TTC3P1	-1.21224	0.0036	0.026323
NLE1	-1.2127	0.0006	0.007015
NPDC1	-1.21293	0.0033	0.024673
SPATA20	-1.21342	0.0014	0.013185
FAM203A	-1.2135	0.00305	0.023264
GDPGP1	-1.21467	0.0028	0.021921
ZBTB42	-1.2151	0.00255	0.020565
KBTBD6	-1.21529	0.00085	0.009052
TRIM16	-1.21691	0.00055	0.006573
LRRC8D	-1.21709	0.00095	0.009766
NRSN2	-1.2179	0.002	0.017269
AHNAK2	-1.21816	0.0004	0.005167
EXO1	-1.2186	0.00055	0.006573
BET1L	-1.21926	0.0008	0.008653
POP1	-1.21927	0.0008	0.008653
THBD	-1.22029	0.0019	0.016639
GPRIN1	-1.22033	0.00135	0.012838
CCDC103	-1.22041	0.0024	0.019631
HOXD8	-1.22115	0.0043	0.029915
CEP78	-1.2218	0.001	0.010186
HOXA10	-1.22211	0.003	0.022946
FZD5	-1.22234	0.0003	0.004153
EIF4EBP1	-1.22252	0.0003	0.004153
LDHA	-1.22283	0.0004	0.005167
C14orf80	-1.22291	0.00315	0.023852
IGSF3	-1.2236	0.0013	0.01252
C1orf216	-1.22394	0.0009	0.009427
C5orf30	-1.22419	0.0006	0.007015
SP9	-1.22507	0.0072	0.043687
NAP1L2	-1.22509	0.0024	0.019631
DSCC1	-1.22543	0.00265	0.021074
KCTD7	-1.22559	0.00085	0.009052
CBS	-1.22598	0.00095	0.009766
VIPR1	-1.22608	0.00755	0.045412
ABHD14B	-1.2265	0.0015	0.013976
TRIM7	-1.22667	0.00405	0.028668
GEMIN4	-1.22693	0.00035	0.004678

SMIM4	-1.22752	0.00365	0.026607
LOC153684	-1.2279	0.0014	0.013185
ZNF284	-1.228	0.0031	0.023602
LRRC45	-1.22836	0.0032	0.024153
SAPCD2	-1.22867	0.0009	0.009427
PLEKHG4	-1.22949	0.0085	0.04934
CDH17	-1.22976	0.00265	0.021074
FADD	-1.22993	0.00065	0.007427
RGS3	-1.23045	0.00215	0.018214
MAP1B	-1.23112	0.0002	0.003062
CDC6	-1.23161	0.00025	0.003659
FIGNL2	-1.23222	0.0017	0.015408
PTGR1	-1.23247	0.0011	0.011035
FLYWCH1	-1.23337	0.002	0.017269
CENPB	-1.23482	0.0009	0.009427
RNF187	-1.23509	0.0013	0.01252
LRP3	-1.23591	0.00215	0.018214
PELO	-1.23618	0.00015	0.002431
HSD17B1	-1.23648	0.00135	0.012838
PDIA4	-1.23673	0.00045	0.005638
HYAL2	-1.23696	0.0014	0.013185
PWWP2B	-1.23703	0.0019	0.016639
GPT2	-1.23732	0.001	0.010186
MRPL55	-1.23738	0.0023	0.019134
SLC6A9	-1.23827	0.0058	0.037289
FAM174B	-1.23835	0.00215	0.018214
PANK1	-1.24123	0.00155	0.014354
MCM2	-1.24218	0.00045	0.005638
WDR76	-1.24327	0.0009	0.009427
GCKR	-1.24361	0.0079	0.046806
SMO	-1.24552	0.00175	0.015698
KEAP1	-1.2457	0.00045	0.005638
CDC42EP1	-1.24577	0.00115	0.011454
PACSIN2	-1.24594	0.00025	0.003659
LPIN3	-1.24616	0.00515	0.034168
MID1IP1	-1.24663	0.0003	0.004153
HMGB2	-1.24731	0.0006	0.007015
NFE2L1	-1.24757	0.00015	0.002431
TRIM35	-1.24791	0.0005	0.006117
LYSMD2	-1.24874	0.00245	0.019913
PTCH1	-1.24896	0.00115	0.011454
PHGDH	-1.24899	0.0003	0.004153
PECR	-1.24947	0.00075	0.008257
LOC100506054	-1.24964	0.00135	0.012838
PPP1R26	-1.25044	0.00095	0.009766
SLC10A3	-1.25064	0.002	0.017269
HINT2	-1.25079	0.002	0.017269
HHEX	-1.25125	0.0007	0.007858
FADS2	-1.25132	0.00055	0.006573

ANKRD2	-1.25417	0.0068	0.041918
EPS8L3	-1.2575	0.00865	0.049984
TBC1D2B	-1.26016	0.0007	0.007858
PPAP2C	-1.26019	0.001	0.010186
GSTM4	-1.26053	0.00365	0.026607
PIK3C2B	-1.26203	0.00035	0.004678
IMPA2	-1.26315	0.0014	0.013185
WDR4	-1.26415	0.0013	0.01252
SIRPA	-1.26686	0.00075	0.008257
SCNN1D	-1.26709	0.007	0.042786
TSPAN13	-1.2684	0.0041	0.028947
SGSH	-1.26855	0.0076	0.045548
ARMC2	-1.26914	0.00795	0.046985
NR2F6	-1.26992	0.00075	0.008257
ZC3HAV1L	-1.27002	0.0006	0.007015
KIF21B	-1.27085	0.00035	0.004678
TMEM141	-1.2711	0.00325	0.024387
GTDC2	-1.27258	0.00185	0.016374
RNFT2	-1.2739	0.0012	0.011824
CCR7	-1.27438	0.0013	0.01252
SCD	-1.27444	0.00015	0.002431
NIT2	-1.27561	0.0008	0.008653
C21orf58	-1.27568	0.0009	0.009427
SLC5A11	-1.27593	0.00125	0.012208
NGFRAP1	-1.27606	0.0008	0.008653
MARCH1	-1.27714	0.00105	0.010623
SRPRB	-1.27733	0.00035	0.004678
PSAT1	-1.27796	0.00025	0.003659
TP53TG1	-1.27966	0.00295	0.022658
LMNB1	-1.28034	0.00095	0.009766
BCAT1	-1.28047	0.0006	0.007015
FAM84B	-1.2808	0.0003	0.004153
NIPSNAP1	-1.28149	0.0009	0.009427
MST1P2	-1.28255	0.00765	0.045782
FADS3	-1.28263	0.00165	0.015053
EPHB4	-1.28479	0.00045	0.005638
GFOD1	-1.28785	0.00385	0.027605
PCYOX1L	-1.28825	0.0015	0.013976
L3HYPDH	-1.29094	0.0008	0.008653
GPD1L	-1.2915	0.00045	0.005638
B4GALT2	-1.29211	0.0006	0.007015
LINC00638	-1.29524	0.0023	0.019134
AURKB	-1.29595	0.00065	0.007427
LZTS2	-1.29609	0.0023	0.019134
TPGS2	-1.29663	0.0064	0.040213
GYG2	-1.29707	0.00195	0.016979
AKAP1	-1.29789	0.0003	0.004153
SLC35C1	-1.29812	0.00065	0.007427
NQO2	-1.29946	0.00025	0.003659

RHPN1	-1.2997	0.00195	0.016979
ZNF74	-1.29998	0.0005	0.006117
NHLRC1	-1.30031	0.0014	0.013185
DGKG	-1.30137	0.00035	0.004678
FAM226B	-1.30158	0.00345	0.025496
SIGMAR1	-1.30341	0.00035	0.004678
G6PD	-1.30356	0.00055	0.006573
GMPPA	-1.30372	0.0012	0.011824
C14orf169	-1.3038	0.0028	0.021921
MYBBP1A	-1.30384	0.0011	0.011035
LHX2	-1.30408	0.0018	0.016069
HR	-1.30424	0.00115	0.011454
AKR1B10	-1.30472	0.0001	0.001745
CA12	-1.30527	0.00015	0.002431
ROGDI	-1.30682	0.00585	0.037553
DHCR24	-1.30768	0.00025	0.003659
CCNF	-1.30794	0.00045	0.005638
AUNIP	-1.30824	0.0003	0.004153
SLC46A1	-1.3102	0.00095	0.009766
HDHD3	-1.3119	0.00075	0.008257
MST1	-1.31297	0.00205	0.017591
NRM	-1.31347	0.002	0.017269
LOC728431	-1.31391	0.0005	0.006117
DNMT3B	-1.31644	0.0008	0.008653
SUMF2	-1.31692	0.00225	0.01885
ZBTB25	-1.31744	0.00015	0.002431
PDXK	-1.31751	0.00025	0.003659
VEGFB	-1.32011	0.00115	0.011454
PCDH9	-1.32169	0.0001	0.001745
MB	-1.32259	0.00415	0.029152
LFNG	-1.32284	0.0019	0.016639
GRAPL	-1.32289	0.00255	0.020565
TUBA1B	-1.32395	0.00015	0.002431
GATS	-1.32451	0.00215	0.018214
TMEM175	-1.32473	0.00265	0.021074
TXNDC5	-1.32627	0.0086	0.049747
GSR	-1.32646	5.00E-05	0.000967
SLC25A11	-1.32681	0.00075	0.008257
PQLC3	-1.32725	0.00095	0.009766
PRKCQ-AS1	-1.32795	0.00065	0.007427
ONECUT1	-1.3283	0.00515	0.034168
MANEAL	-1.3295	0.00055	0.006573
MFSD3	-1.33043	0.00225	0.01885
NQO1	-1.33174	0.0002	0.003062
PIGM	-1.33202	0.00015	0.002431
FAM64A	-1.33346	0.00035	0.004678
AGTRAP	-1.33364	0.00115	0.011454
F8A1,F8A3	-1.33533	0.00595	0.038106
CRTAP	-1.33608	5.00E-05	0.000967

TTC39B	-1.33635	0.0015	0.013976
GINS2	-1.33677	0.0004	0.005167
ABCA12	-1.3372	0.00185	0.016374
TIGD2	-1.34038	0.00075	0.008257
LYNX1	-1.343	0.00815	0.047894
CDPF1	-1.34415	0.0009	0.009427
TUB	-1.34439	0.00055	0.006573
C10orf114	-1.3453	0.0009	0.009427
VLDLR	-1.34551	0.0002	0.003062
RFXAP	-1.34744	0.00225	0.01885
WBSCR27	-1.34814	0.00405	0.028668
PANX2	-1.34935	0.0026	0.020846
FAM213B	-1.3525	0.00195	0.016979
PFN2	-1.35341	0.00025	0.003659
ZFP62	-1.35539	0.0002	0.003062
MRPL34	-1.35562	0.00065	0.007427
LOC344595	-1.35671	0.0015	0.013976
SLC37A4	-1.35698	0.00075	0.008257
PAOX	-1.35768	0.00325	0.024387
ZNF771	-1.36039	0.005	0.033413
DGAT2	-1.36205	0.00165	0.015053
BLOC1S4	-1.36253	0.0003	0.004153
RAB3D	-1.36309	0.0003	0.004153
CDC25A	-1.3647	5.00E-05	0.000967
TTC30A	-1.36479	0.0002	0.003062
TUBG2	-1.36499	0.0006	0.007015
RAP1GAP	-1.36503	0.0001	0.001745
PLCXD1	-1.36513	0.00185	0.016374
EPDR1	-1.36696	0.00015	0.002431
RRM2	-1.36786	0.0001	0.001745
DNLZ	-1.36894	0.0037	0.026819
DGCR6	-1.36971	0.00585	0.037553
FGL1	-1.37103	0.00125	0.012208
CHST1	-1.37303	0.00425	0.029666
TSPO	-1.37449	0.00135	0.012838
NKAIN1	-1.37459	0.0037	0.026819
LRRC16B	-1.37502	0.00175	0.015698
IGSF8	-1.3751	0.00065	0.007427
STBD1	-1.37617	0.0002	0.003062
GLTPD1	-1.37936	0.0007	0.007858
KIAA0319	-1.38	5.00E-05	0.000967
DHRS4L1	-1.38036	0.00655	0.040923
PHF15	-1.38313	5.00E-05	0.000967
FAM102A	-1.3836	0.0003	0.004153
TWIST1	-1.38449	0.00605	0.038539
TOB1	-1.38728	5.00E-05	0.000967
AMBP	-1.38865	0.00175	0.015698
PPP1R3E	-1.38883	0.0003	0.004153
CDR2L	-1.39012	0.0002	0.003062

MEX3B	-1.39099	0.00055	0.006573
HTATSF1P2	-1.39319	0.0001	0.001745
MRPL12	-1.39378	0.00025	0.003659
ASIC5	-1.39414	0.00265	0.021074
CABYR	-1.39422	0.0003	0.004153
C1orf220	-1.39447	0.0005	0.006117
CYP4F11	-1.39608	5.00E-05	0.000967
LOC150381	-1.39696	0.0023	0.019134
CBX4	-1.39734	5.00E-05	0.000967
GCLM	-1.39738	0.0001	0.001745
SEC14L4	-1.39912	0.00055	0.006573
ALDH1L2	-1.39935	0.0004	0.005167
RAB3IL1	-1.40174	0.0003	0.004153
ANKS4B	-1.40179	0.00065	0.007427
LOC440894	-1.40508	0.00075	0.008257
PTCD2	-1.40717	0.0003	0.004153
ARHGEF39	-1.40753	0.0002	0.003062
FXYD2	-1.40792	0.0005	0.006117
SYBU	-1.40955	5.00E-05	0.000967
TTLL12	-1.40972	0.00045	0.005638
NOL3	-1.40994	0.00845	0.049135
OSR2	-1.41046	0.0008	0.008653
GALNT12	-1.41173	0.0011	0.011035
G6PC3	-1.41385	0.0003	0.004153
C1orf106	-1.41414	0.0001	0.001745
SPDEF	-1.41433	0.0018	0.016069
SKIDA1	-1.41467	0.0003	0.004153
LOC348761	-1.41613	0.00205	0.017591
CARNS1	-1.417	0.0035	0.025785
NIPSNAP3B	-1.41859	0.0052	0.034431
ZNF488	-1.41882	0.0001	0.001745
GALK1	-1.41885	0.0003	0.004153
PARD6A	-1.42049	0.0042	0.029379
CTBP1-AS1	-1.42138	0.0002	0.003062
C5	-1.42263	0.0002	0.003062
CA7	-1.42274	0.0073	0.044132
SLC45A1	-1.4236	0.0011	0.011035
SRD5A1	-1.42476	0.0004	0.005167
CAT	-1.42588	0.00015	0.002431
SGK2	-1.42603	0.0002	0.003062
CCNJL	-1.42762	5.00E-05	0.000967
ZNF775	-1.42801	0.00145	0.01361
ZBTB7B	-1.42887	0.0002	0.003062
CPOX	-1.42903	0.0001	0.001745
GM2A	-1.43076	0.00015	0.002431
FAM111B	-1.43241	0.0006	0.007015
F7	-1.43257	0.0011	0.011035
SLC1A4	-1.43416	5.00E-05	0.000967
ZNF48	-1.43547	0.00015	0.002431

ADD2	-1.43815	5.00E-05	0.000967
MAMSTR	-1.43833	0.00175	0.015698
CDX2	-1.44005	0.00285	0.022187
CTDSPL	-1.44047	0.0004	0.005167
SLC17A3	-1.44169	0.0048	0.032482
GAS2L1	-1.4424	0.00075	0.008257
NTHL1	-1.44431	0.0007	0.007858
C1orf74	-1.44444	0.00045	0.005638
LOC151009	-1.44449	0.0066	0.041051
PSD4	-1.44529	0.00045	0.005638
CTAGE7P	-1.44742	0.00195	0.016979
NEIL3	-1.4477	0.0001	0.001745
C2orf72	-1.44771	0.0003	0.004153
LLGL2	-1.44774	0.00125	0.012208
HEXIM2	-1.44848	0.00055	0.006573
RAB20	-1.44995	0.0007	0.007858
WDR16	-1.45029	0.00075	0.008257
TMEM161A	-1.45488	0.00065	0.007427
OSR1	-1.45739	0.0005	0.006117
C3orf72	-1.45852	0.00055	0.006573
RBFA	-1.45892	5.00E-05	0.000967
FOXD2-AS1	-1.4624	0.00015	0.002431
TUBB	-1.46273	5.00E-05	0.000967
S1PR5	-1.46279	0.001	0.010186
B3GALT6	-1.46378	0.00015	0.002431
TRIM16L	-1.46609	5.00E-05	0.000967
TIGD5	-1.46779	0.00025	0.003659
MRPS34	-1.46796	0.0003	0.004153
SEL1L3	-1.4699	5.00E-05	0.000967
HOXB13	-1.47208	0.00035	0.004678
FGD3	-1.47807	0.00235	0.019365
C22orf29	-1.47851	5.00E-05	0.000967
PLEKHH3	-1.47925	0.0004	0.005167
PARM1	-1.48051	0.0017	0.015408
PLEKHF1	-1.48137	0.0002	0.003062
KCTD15	-1.48302	5.00E-05	0.000967
XK	-1.48358	0.0004	0.005167
TCEAL8	-1.48566	0.0006	0.007015
TNFRSF1A	-1.48569	5.00E-05	0.000967
LINC00086	-1.4859	0.00265	0.021074
MCM5	-1.48793	0.00015	0.002431
ZNF30	-1.48973	0.0003	0.004153
FEN1	-1.48983	5.00E-05	0.000967
ZNF526	-1.49008	5.00E-05	0.000967
GCNT3	-1.49032	5.00E-05	0.000967
FLJ44511	-1.49035	0.00075	0.008257
PREB	-1.49094	0.0003	0.004153
TXNRD1	-1.49137	5.00E-05	0.000967
FAM20C	-1.49264	0.0002	0.003062

SDR42E1	-1.4929	0.00085	0.009052
USP51	-1.49656	5.00E-05	0.000967
CENPI	-1.49745	0.0004	0.005167
MCIN	-1.50097	0.00055	0.006573
ZNF786	-1.50144	5.00E-05	0.000967
ASAH2B	-1.50152	0.0048	0.032482
PALM3	-1.5017	0.0066	0.041051
SLC48A1	-1.50183	0.0047	0.031987
AP3M2	-1.50259	5.00E-05	0.000967
IL1RAPL2	-1.50337	0.0051	0.033918
KLF13	-1.50351	0.0001	0.001745
SVIP	-1.5045	0.00025	0.003659
C15orf39	-1.50453	0.0001	0.001745
RERG	-1.50617	0.0013	0.01252
ZNF396	-1.50646	0.00165	0.015053
TMEM246	-1.51081	5.00E-05	0.000967
RAB40B	-1.51124	0.0003	0.004153
MNX1	-1.51147	0.0002	0.003062
HTR1D	-1.51209	0.00015	0.002431
RCAN3	-1.51691	0.0001	0.001745
SPTBN4	-1.51902	0.00295	0.022658
ZNF517	-1.52112	0.00025	0.003659
C17orf70	-1.52201	0.0001	0.001745
LOC158572	-1.52206	0.0008	0.008653
ZBTB14	-1.5222	5.00E-05	0.000967
C10orf35	-1.52248	0.0002	0.003062
SPC24	-1.52291	0.00065	0.007427
TMEM177	-1.52303	5.00E-05	0.000967
GLTPD2	-1.52462	0.00315	0.023852
C9orf142	-1.52498	0.0008	0.008653
SLC52A2	-1.52523	0.0002	0.003062
VPS37D	-1.52643	0.0006	0.007015
PRDM13	-1.52798	0.0004	0.005167
LRRC20	-1.52851	0.00015	0.002431
CDCA3	-1.52953	5.00E-05	0.000967
SLC29A3	-1.52967	5.00E-05	0.000967
FRAT2	-1.53153	5.00E-05	0.000967
LOC100129917	-1.53247	0.00205	0.017591
SLC27A2	-1.5367	0.00025	0.003659
TTLL7	-1.53736	0.0014	0.013185
THBS3	-1.53757	0.00015	0.002431
NKX2-5	-1.53773	0.00035	0.004678
SKP2	-1.53902	0.00045	0.005638
LOC388906	-1.53922	0.0009	0.009427
GPT	-1.5409	0.001	0.010186
UNG	-1.54247	5.00E-05	0.000967
ACSBG2	-1.54372	0.00335	0.024946
PELI3	-1.54461	5.00E-05	0.000967
TTLL6	-1.54753	0.0005	0.006117

AMER1	-1.54837	5.00E-05	0.000967
RTKN	-1.54926	0.0001	0.001745
LBH	-1.54993	5.00E-05	0.000967
C11orf45	-1.55453	0.00115	0.011454
TRIB3	-1.55609	5.00E-05	0.000967
HEIH	-1.55642	0.00045	0.005638
NR0B1	-1.55656	5.00E-05	0.000967
NHLRC3	-1.55884	0.0003	0.004153
FUOM	-1.56015	0.002	0.017269
LOC100507032	-1.56025	5.00E-05	0.000967
GPR135	-1.56133	0.00035	0.004678
IMP3	-1.56281	5.00E-05	0.000967
CCDC87	-1.56437	0.00025	0.003659
AMACR	-1.56773	0.0008	0.008653
C11orf92	-1.57083	0.0013	0.01252
TMEM102	-1.57244	0.0008	0.008653
C21orf67	-1.57288	0.0003	0.004153
STOX2	-1.5747	5.00E-05	0.000967
NME4	-1.575	5.00E-05	0.000967
ITGAD	-1.57563	0.0008	0.008653
RMI2	-1.57612	0.00045	0.005638
EPHX1	-1.57644	5.00E-05	0.000967
UBL4A	-1.57752	5.00E-05	0.000967
SRD5A1P1	-1.57922	0.00375	0.027122
FAM86EP	-1.57986	5.00E-05	0.000967
SIRPB1	-1.58002	0.00035	0.004678
TMEM53	-1.5814	0.00035	0.004678
TM4SF20	-1.58204	0.0001	0.001745
CLIC3	-1.58464	0.00505	0.033693
SFXN2	-1.58572	0.0001	0.001745
ABCC2	-1.58706	5.00E-05	0.000967
CLGN	-1.58904	0.00055	0.006573
ST6GALNAC4	-1.5891	0.00035	0.004678
CRIP2	-1.58979	0.00135	0.012838
PGD	-1.59007	5.00E-05	0.000967
NEURL1B	-1.59021	0.00015	0.002431
FIGNL1	-1.59177	5.00E-05	0.000967
GPC1	-1.59213	5.00E-05	0.000967
C19orf48	-1.59574	5.00E-05	0.000967
DNAJB9	-1.59638	0.0003	0.004153
HPS6	-1.59689	5.00E-05	0.000967
THEM6	-1.59765	5.00E-05	0.000967
PRR5	-1.60192	0.0077	0.046014
IDH1	-1.60239	5.00E-05	0.000967
TMEM139	-1.60559	0.0002	0.003062
MAGEH1	-1.60706	5.00E-05	0.000967
FAM217B	-1.61006	0.00065	0.007427
ZNF792	-1.61088	5.00E-05	0.000967
ATP6V0E2	-1.61184	0.00225	0.01885

KBTBD7	-1.61209	5.00E-05	0.000967
C9orf117	-1.61323	0.00235	0.019365
DEFB1	-1.61349	0.00825	0.048311
ADCK2	-1.61449	5.00E-05	0.000967
PDGFB	-1.61608	0.0001	0.001745
BATF3	-1.6174	5.00E-05	0.000967
SYTL1	-1.61804	0.0034	0.025194
TUSC1	-1.61957	0.00015	0.002431
TP53I11	-1.62024	5.00E-05	0.000967
PGP	-1.62204	5.00E-05	0.000967
UNKL	-1.62311	0.0001	0.001745
PYCR1	-1.62559	5.00E-05	0.000967
RIMBP3	-1.62598	0.00035	0.004678
RPUSD1	-1.62804	5.00E-05	0.000967
HILPDA	-1.62845	5.00E-05	0.000967
PSRC1	-1.62954	5.00E-05	0.000967
CAMKK1	-1.63116	5.00E-05	0.000967
PIM1	-1.63136	5.00E-05	0.000967
SOX12	-1.63254	5.00E-05	0.000967
FGFR3	-1.63305	0.0002	0.003062
KCTD17	-1.63532	0.0003	0.004153
C16orf59	-1.63556	0.0003	0.004153
RAB3A	-1.63837	0.00235	0.019365
KLHL31	-1.63871	5.00E-05	0.000967
NME3	-1.64003	0.0004	0.005167
CKAP4	-1.64022	5.00E-05	0.000967
NUDT18	-1.64026	5.00E-05	0.000967
GATA3-AS1	-1.64175	0.00315	0.023852
ABHD4	-1.64183	5.00E-05	0.000967
SNX33	-1.6435	5.00E-05	0.000967
RIBC2	-1.65177	0.0002	0.003062
B4GALNT1	-1.65314	5.00E-05	0.000967
PCSK9	-1.65688	5.00E-05	0.000967
LINC00516	-1.65913	0.0006	0.007015
CPLX2	-1.66019	5.00E-05	0.000967
P2RY6	-1.66315	0.00015	0.002431
FGF19	-1.66323	0.00265	0.021074
SNTB1	-1.66378	5.00E-05	0.000967
USH1C	-1.66528	0.0002	0.003062
SLC9A3R2	-1.6653	5.00E-05	0.000967
RPP25	-1.66675	5.00E-05	0.000967
THYN1	-1.6697	0.0003	0.004153
DCLRE1B	-1.67012	5.00E-05	0.000967
KCNK5	-1.67903	5.00E-05	0.000967
HSF2BP	-1.67924	0.00615	0.039026
HS3ST6	-1.67938	0.00095	0.009766
ALDH1A1	-1.67946	5.00E-05	0.000967
C9orf69	-1.67968	5.00E-05	0.000967
FAM127C	-1.68166	0.00095	0.009766

ZBTB12	-1.68212	0.0042	0.029379
VAV3	-1.68227	5.00E-05	0.000967
E2F8	-1.68325	5.00E-05	0.000967
CRIP1	-1.68357	5.00E-05	0.000967
CYP4F2	-1.68375	0.0014	0.013185
DUSP23	-1.68917	5.00E-05	0.000967
SLC29A4	-1.693	0.00025	0.003659
SLC7A7	-1.6939	5.00E-05	0.000967
OCEL1	-1.6962	0.00045	0.005638
NUGGC	-1.70004	0.0004	0.005167
ANKRD9	-1.70589	0.0005	0.006117
CDCA7	-1.70598	5.00E-05	0.000967
S100P	-1.71189	5.00E-05	0.000967
AKNA	-1.7127	5.00E-05	0.000967
TST	-1.71448	0.0009	0.009427
CLDND2	-1.71545	0.00095	0.009766
ALDH1B1	-1.71589	5.00E-05	0.000967
APOH	-1.71611	0.0008	0.008653
CBR1	-1.71677	5.00E-05	0.000967
GPNMB	-1.71716	0.00055	0.006573
DHRS13	-1.72237	5.00E-05	0.000967
LOC257396	-1.72754	5.00E-05	0.000967
PFN4	-1.7301	0.0029	0.022408
ZNF232	-1.73383	5.00E-05	0.000967
IDH2	-1.73631	0.00015	0.002431
C15orf55	-1.73814	0.00035	0.004678
CLEC11A	-1.74353	0.00125	0.012208
C1orf233	-1.7478	0.00055	0.006573
CENPM	-1.75297	5.00E-05	0.000967
PRR15	-1.75671	0.00165	0.015053
KRT4	-1.75773	5.00E-05	0.000967
E2F1	-1.77364	5.00E-05	0.000967
SLC7A5	-1.7746	5.00E-05	0.000967
RNASET2	-1.77565	0.0016	0.014734
RGS14	-1.77713	0.0002	0.003062
SPP1	-1.77936	5.00E-05	0.000967
FANCF	-1.77951	5.00E-05	0.000967
SCRT2	-1.78084	0.00085	0.009052
ANXA13	-1.78263	0.0005	0.006117
CORO2A	-1.78679	5.00E-05	0.000967
WFIKKN1	-1.78682	0.001	0.010186
KCNC4	-1.78711	5.00E-05	0.000967
NINJ1	-1.78726	5.00E-05	0.000967
HSPA12B	-1.78853	5.00E-05	0.000967
FLVCR1-AS1	-1.78991	0.00035	0.004678
CEBPA-AS1	-1.79145	5.00E-05	0.000967
DEPTOR	-1.79412	0.0003	0.004153
RPL13P5	-1.79426	5.00E-05	0.000967
PAQR4	-1.79565	0.0002	0.003062

ID1	-1.80178	5.00E-05	0.000967
PNMA2	-1.80926	5.00E-05	0.000967
C1QTNF6	-1.81092	5.00E-05	0.000967
LOC440518	-1.81125	0.0001	0.001745
ZBTB47	-1.8117	5.00E-05	0.000967
FAM78A	-1.8131	5.00E-05	0.000967
ZBTB32	-1.81797	5.00E-05	0.000967
HNF4A	-1.82436	5.00E-05	0.000967
ABCB6	-1.83151	5.00E-05	0.000967
THNSL1	-1.8325	5.00E-05	0.000967
MIRLET7BHG	-1.83405	5.00E-05	0.000967
AGR2	-1.83571	5.00E-05	0.000967
PRSS16	-1.83614	0.0001	0.001745
RAB37	-1.839	5.00E-05	0.000967
RAB38	-1.84257	0.0001	0.001745
FZD2	-1.84564	5.00E-05	0.000967
C9orf50	-1.85444	0.0028	0.021921
MIR210HG	-1.85479	5.00E-05	0.000967
CCND3	-1.85904	5.00E-05	0.000967
ZNF688	-1.86036	5.00E-05	0.000967
C17orf82	-1.86133	0.0012	0.011824
C10orf91	-1.86476	0.0005	0.006117
CEBPA	-1.86696	5.00E-05	0.000967
CBX2	-1.86765	0.00015	0.002431
A4GNT	-1.8763	0.00045	0.005638
CXXC5	-1.87877	5.00E-05	0.000967
CAPN5	-1.87981	5.00E-05	0.000967
HJURP	-1.88251	5.00E-05	0.000967
ABHD15	-1.88407	5.00E-05	0.000967
GJD3	-1.89285	5.00E-05	0.000967
JMJD4	-1.89351	0.00465	0.031738
FAM86C2P	-1.89506	5.00E-05	0.000967
THAP8	-1.89754	5.00E-05	0.000967
DPYSL2	-1.89806	5.00E-05	0.000967
SLC16A13	-1.8982	0.0001	0.001745
NKG7	-1.90342	0.00795	0.046985
KAZALD1	-1.90358	5.00E-05	0.000967
MAFG-AS1	-1.90359	5.00E-05	0.000967
PAQR9	-1.90391	5.00E-05	0.000967
INHBB	-1.9086	5.00E-05	0.000967
ZDHHC8P1	-1.91265	5.00E-05	0.000967
NS3BP	-1.91788	0.0001	0.001745
E2F2	-1.92089	5.00E-05	0.000967
DDIT4L	-1.92126	5.00E-05	0.000967
RTN4R	-1.92727	0.0003	0.004153
SMKR1	-1.93354	0.00035	0.004678
ACP5	-1.93729	0.00025	0.003659
ZFP69B	-1.93919	5.00E-05	0.000967
KCNS3	-1.94149	0.0013	0.01252

SCTR	-1.94783	0.0006	0.007015
KRT82	-1.95046	0.00055	0.006573
MANSC1	-1.95135	5.00E-05	0.000967
SRXN1	-1.95138	5.00E-05	0.000967
MBLAC2	-1.96263	5.00E-05	0.000967
SLC26A1	-1.9631	0.00075	0.008257
F2RL2	-1.96336	5.00E-05	0.000967
TSKU	-1.97016	5.00E-05	0.000967
C19orf60	-1.97101	0.00015	0.002431
LINC00858	-1.97339	5.00E-05	0.000967
SAMD13	-1.97789	0.00535	0.035214
AKR1C3	-1.97937	5.00E-05	0.000967
LOC101101776	-1.97967	0.0001	0.001745
CD40LG	-1.99981	0.00015	0.002431
AKR1C1	-2.00819	5.00E-05	0.000967
CDH1	-2.0205	5.00E-05	0.000967
FGG	-2.03029	5.00E-05	0.000967
PNMA6C	-2.03766	0.00015	0.002431
MPST	-2.03872	0.001	0.010186
ABCA17P	-2.04321	5.00E-05	0.000967
GPR162	-2.04949	5.00E-05	0.000967
UFSP1	-2.05239	5.00E-05	0.000967
B3GNT1	-2.05467	5.00E-05	0.000967
ZNF467	-2.06864	0.00125	0.012208
RASSF7	-2.06928	5.00E-05	0.000967
AKR1C4	-2.06954	5.00E-05	0.000967
HNF1A-AS1	-2.07078	5.00E-05	0.000967
LINC00235	-2.07936	5.00E-05	0.000967
CRLF1	-2.08041	0.0001	0.001745
KHK	-2.08072	0.0031	0.023602
LOC728537	-2.08298	0.0001	0.001745
HLA-DMB	-2.08753	0.0002	0.003062
MSC	-2.09568	5.00E-05	0.000967
CSTL1	-2.12022	0.00785	0.046626
LHX9	-2.12145	0.0001	0.001745
MARVELD1	-2.13719	5.00E-05	0.000967
DISP2	-2.14347	5.00E-05	0.000967
LOC645249	-2.14386	5.00E-05	0.000967
LIPT2	-2.16165	5.00E-05	0.000967
SSR4P1	-2.16355	0.0003	0.004153
GPRIN2	-2.17526	5.00E-05	0.000967
TNFRSF13C	-2.1773	0.0054	0.035445
LOC554206	-2.19167	0.00025	0.003659
FGA	-2.2224	5.00E-05	0.000967
LOC148696	-2.23235	5.00E-05	0.000967
FAM194A	-2.23749	5.00E-05	0.000967
EPN3	-2.24433	5.00E-05	0.000967
KRTAP4-1	-2.25754	0.00195	0.016979
GPR20	-2.2844	5.00E-05	0.000967

CYP4F12	-2.28547	5.00E-05	0.000967
CPN1	-2.29168	0.00035	0.004678
AKR1C2	-2.29206	5.00E-05	0.000967
NT5M	-2.29349	5.00E-05	0.000967
NTN3	-2.30378	0.00045	0.005638
C12orf68	-2.33257	5.00E-05	0.000967
TMEM52	-2.34252	0.00795	0.046985
EMILIN1	-2.349	5.00E-05	0.000967
KIF12	-2.35036	5.00E-05	0.000967
METTL7B	-2.36774	5.00E-05	0.000967
RNASE4	-2.38495	0.0002	0.003062
USP29	-2.38975	5.00E-05	0.000967
CYP4F3	-2.39757	5.00E-05	0.000967
ANG	-2.42336	0.0026	0.020846
NUPR1	-2.44519	5.00E-05	0.000967
OSGIN1	-2.49036	5.00E-05	0.000967
SFRP4	-2.59932	5.00E-05	0.000967
ASCL5	-2.6103	0.00205	0.017591
SLC40A1	-2.64261	5.00E-05	0.000967
TMEM37	-2.71348	5.00E-05	0.000967
SHH	-2.71967	5.00E-05	0.000967
HMOX1	-2.7257	5.00E-05	0.000967
LOC100506229	-2.74899	5.00E-05	0.000967
ATOH8	-2.78861	5.00E-05	0.000967
KLHDC7A	-3.08352	5.00E-05	0.000967

[(a)] probability of your false positive

[(b)] adjusted p-values found using an optimised False Discovery Rate (FDR) approach.

Table S2. Differentially expressed genes in BTV8ΔNS4- compared to mock-infected A549 cells.

Gene symbol	Fold change (log2)	P-value [(a)]	Q-value [(b)] (<0.05)
CH25H	7.15317	0.0054	0.040509
IFNB1	7.09201	5.00E-05	0.001089
IFNL1	6.72284	5.00E-05	0.001089
IFNL2	6.50831	5.00E-05	0.001089
MX1	6.46909	5.00E-05	0.001089
IFNL3	6.45399	5.00E-05	0.001089
OASL	6.45306	5.00E-05	0.001089
IFIT2	6.40381	5.00E-05	0.001089
RSAD2	6.29918	5.00E-05	0.001089
IFIT1	6.24563	5.00E-05	0.001089
CXCL11	6.16231	0.00085	0.010312
IFIT3	6.14966	5.00E-05	0.001089
CMPK2	6.00184	5.00E-05	0.001089
IFI44	5.98641	5.00E-05	0.001089
MX2	5.94949	5.00E-05	0.001089

CCR4	5.81579	5.00E-05	0.001089
IFI27	5.63631	5.00E-05	0.001089
DDX58	5.59636	5.00E-05	0.001089
OAS2	5.52776	5.00E-05	0.001089
IFIH1	5.50756	5.00E-05	0.001089
CA1	5.49294	5.00E-05	0.001089
ISG15	5.44795	5.00E-05	0.001089
FOSB	5.44563	5.00E-05	0.001089
EGR2	5.40941	5.00E-05	0.001089
FOS	5.31981	5.00E-05	0.001089
IL8	5.29486	5.00E-05	0.001089
KLRC2	5.20089	5.00E-05	0.001089
EGR1	5.17407	0.0001	0.001954
SLC1A3	5.1674	5.00E-05	0.001089
EGR4	5.15848	5.00E-05	0.001089
BATF2	5.06808	5.00E-05	0.001089
HERC5	5.0189	5.00E-05	0.001089
IFI6	5.01293	5.00E-05	0.001089
IFNL4	4.92876	5.00E-05	0.001089
ARL14	4.92528	5.00E-05	0.001089
IL1RL1	4.89731	5.00E-05	0.001089
RFPL4A	4.85423	0.0013	0.014165
SAMD9	4.84687	5.00E-05	0.001089
TNFSF4	4.81036	5.00E-05	0.001089
IFITM1	4.74284	0.0012	0.013303
IFI16	4.70346	5.00E-05	0.001089
DDX60	4.64623	5.00E-05	0.001089
CCL5	4.60728	5.00E-05	0.001089
EGR3	4.57014	5.00E-05	0.001089
GBP1	4.56184	5.00E-05	0.001089
PLEKHA4	4.45365	5.00E-05	0.001089
USH1G	4.28429	5.00E-05	0.001089
TRIM22	4.28249	5.00E-05	0.001089
IL6	4.28046	5.00E-05	0.001089
DHX58	4.2557	5.00E-05	0.001089
DDX60L	4.19596	5.00E-05	0.001089
CREB5	4.17979	5.00E-05	0.001089
ALOXE3	4.17345	5.00E-05	0.001089
TYRP1	4.16418	5.00E-05	0.001089
MXD1	4.12641	5.00E-05	0.001089
TRAF1	4.10313	5.00E-05	0.001089
ADH1C	4.09581	0.0026	0.023711
LOC284344	4.09324	0.00355	0.029785
KLRC4	4.09282	0.0024	0.022413
HIST1H1T	4.08444	5.00E-05	0.001089
ZC3HAV1	4.06846	5.00E-05	0.001089

MC5R	4.0662	0.00015	0.002736
IL32	4.04317	5.00E-05	0.001089
HELZ2	4.02699	5.00E-05	0.001089
IRGM	4.0067	5.00E-05	0.001089
KLRC3	4.00329	5.00E-05	0.001089
ZCCHC12	3.9714	0.0008	0.009825
XAF1	3.95484	5.00E-05	0.001089
GPR50	3.9358	5.00E-05	0.001089
IRF9	3.93383	5.00E-05	0.001089
KDM6B	3.93109	5.00E-05	0.001089
TNFRSF9	3.92781	5.00E-05	0.001089
OAS1	3.92679	5.00E-05	0.001089
ANKRD1	3.92282	5.00E-05	0.001089
MAFF	3.88259	5.00E-05	0.001089
FRG2B	3.86752	0.00555	0.041342
CCDC144NL	3.85753	5.00E-05	0.001089
ATF3	3.85677	5.00E-05	0.001089
TNF	3.82417	5.00E-05	0.001089
NFKBIZ	3.81291	5.00E-05	0.001089
JUN	3.80519	5.00E-05	0.001089
DHRS2	3.78842	5.00E-05	0.001089
C20orf195	3.78785	5.00E-05	0.001089
PLA2G4C	3.77897	5.00E-05	0.001089
LOC643201	3.75804	5.00E-05	0.001089
ARL14EPL	3.75766	0.00045	0.006411
PARP9	3.74982	5.00E-05	0.001089
HLA-B	3.7451	5.00E-05	0.001089
DUSP8	3.74413	0.0036	0.030077
RGMA	3.72102	5.00E-05	0.001089
GPR1	3.68597	5.00E-05	0.001089
NPTX2	3.68078	0.00095	0.011119
NID1	3.67198	5.00E-05	0.001089
ITGAM	3.66731	5.00E-05	0.001089
HIST1H2BG	3.66483	5.00E-05	0.001089
EFNB2	3.66458	5.00E-05	0.001089
OAS3	3.64321	5.00E-05	0.001089
GBP5	3.62176	5.00E-05	0.001089
IRF1	3.60272	5.00E-05	0.001089
CD83	3.55657	5.00E-05	0.001089
PPP4R4	3.52592	5.00E-05	0.001089
FAM71A	3.51333	5.00E-05	0.001089
SLCO5A1	3.49092	5.00E-05	0.001089
TRANK1	3.48645	5.00E-05	0.001089
CCL20	3.47834	5.00E-05	0.001089
GBP2	3.47703	5.00E-05	0.001089
PLSCR1	3.47447	5.00E-05	0.001089

DDIT3	3.47373	5.00E-05	0.001089
CXCR4	3.44355	5.00E-05	0.001089
HIST1H3G	3.43807	5.00E-05	0.001089
HSPA6	3.4128	5.00E-05	0.001089
CD274	3.41251	5.00E-05	0.001089
HLA-F	3.3983	5.00E-05	0.001089
PPP1R15A	3.39269	5.00E-05	0.001089
CETP	3.39011	0.00065	0.008431
SAMD9L	3.37648	5.00E-05	0.001089
ACTN2	3.37484	5.00E-05	0.001089
HIST1H2AE	3.3748	5.00E-05	0.001089
TEX37	3.36575	0.0009	0.010731
TNFAIP3	3.36358	5.00E-05	0.001089
TNFAIP6	3.34146	0.0007	0.008891
OR2B6	3.33299	5.00E-05	0.001089
DNAH3	3.3326	0.00065	0.008431
STAT2	3.32918	5.00E-05	0.001089
ICAM1	3.3243	5.00E-05	0.001089
NLRC5	3.31997	5.00E-05	0.001089
CCRN4L	3.30364	5.00E-05	0.001089
IL23A	3.28716	5.00E-05	0.001089
HERC6	3.27896	5.00E-05	0.001089
RTP4	3.27084	0.006	0.043714
C6orf58	3.26956	5.00E-05	0.001089
CPA2	3.26358	0.0015	0.015773
SAMHD1	3.25601	5.00E-05	0.001089
TULP2	3.2497	5.00E-05	0.001089
CYLD	3.2473	5.00E-05	0.001089
SP110	3.22382	5.00E-05	0.001089
HIST4H4	3.2222	5.00E-05	0.001089
NR4A3	3.20964	5.00E-05	0.001089
SOD2	3.20927	5.00E-05	0.001089
PARP14	3.19316	5.00E-05	0.001089
FCGR1B	3.18241	5.00E-05	0.001089
HSPA7	3.17803	5.00E-05	0.001089
BIRC3	3.17266	5.00E-05	0.001089
ARID5B	3.16888	5.00E-05	0.001089
HMGCS1	3.16545	5.00E-05	0.001089
IFIT1B	3.16319	5.00E-05	0.001089
STC1	3.1595	5.00E-05	0.001089
KLRC1	3.14589	0.00125	0.013761
SEMA3D	3.12829	5.00E-05	0.001089
FAM46C	3.11445	5.00E-05	0.001089
TAAR3	3.09548	0.0002	0.003428
RHCG	3.09319	5.00E-05	0.001089
DUSP10	3.09194	5.00E-05	0.001089

GBP3	3.08986	5.00E-05	0.001089
HDAC9	3.08617	5.00E-05	0.001089
IRF7	3.07934	5.00E-05	0.001089
LTB	3.07898	5.00E-05	0.001089
CXCL1	3.06428	5.00E-05	0.001089
SOCS1	3.05133	5.00E-05	0.001089
OR13A1	3.04751	0.00065	0.008431
PTGER4	3.0431	5.00E-05	0.001089
CD163L1	3.02422	5.00E-05	0.001089
NEUROD4	3.00582	5.00E-05	0.001089
CFB	3.00552	5.00E-05	0.001089
N4BP3	2.97401	5.00E-05	0.001089
PDE11A	2.95943	5.00E-05	0.001089
USP18	2.94146	5.00E-05	0.001089
ISG20	2.93439	5.00E-05	0.001089
SELPLG	2.92866	5.00E-05	0.001089
B2M	2.92245	5.00E-05	0.001089
STAT1	2.91479	5.00E-05	0.001089
ZEB2	2.91115	5.00E-05	0.001089
SPATA9	2.90458	0.0029	0.025597
ZNFX1	2.90063	5.00E-05	0.001089
TDRD7	2.89935	5.00E-05	0.001089
LRRC15	2.89419	5.00E-05	0.001089
PTGS2	2.89416	5.00E-05	0.001089
OTUD1	2.8906	5.00E-05	0.001089
PARP10	2.88626	5.00E-05	0.001089
CYR61	2.8776	5.00E-05	0.001089
NR4A1	2.87271	5.00E-05	0.001089
H2AFJ	2.86112	5.00E-05	0.001089
LMO2	2.85718	5.00E-05	0.001089
OR2B2	2.85657	5.00E-05	0.001089
SLC22A1	2.85512	5.00E-05	0.001089
DTX3L	2.84697	5.00E-05	0.001089
FAP	2.84298	5.00E-05	0.001089
MCL1	2.84238	5.00E-05	0.001089
HCAR3	2.8375	5.00E-05	0.001089
PARP12	2.8328	5.00E-05	0.001089
CD200R1	2.8249	0.0001	0.001954
LAMP3	2.82444	5.00E-05	0.001089
C5orf27	2.80215	5.00E-05	0.001089
DSC1	2.80035	5.00E-05	0.001089
HIST2H2BC	2.78173	5.00E-05	0.001089
SNAPC1	2.78079	5.00E-05	0.001089
PRDX2	2.77762	5.00E-05	0.001089
UBD	2.76809	0.0004	0.005861
GPR176	2.76434	5.00E-05	0.001089

ZFP36	2.76419	5.00E-05	0.001089
FAM46A	2.75571	5.00E-05	0.001089
APOL6	2.75533	5.00E-05	0.001089
IFITM3	2.75161	5.00E-05	0.001089
HLA-E	2.74928	5.00E-05	0.001089
KRT17	2.74921	0.00565	0.041892
PMAIP1	2.74852	5.00E-05	0.001089
ZFPM2	2.74752	5.00E-05	0.001089
INHBA	2.74218	5.00E-05	0.001089
TEX14	2.74156	5.00E-05	0.001089
APOL1	2.73666	5.00E-05	0.001089
HLA-H	2.72817	5.00E-05	0.001089
APOL2	2.70693	5.00E-05	0.001089
ZNF80	2.7	5.00E-05	0.001089
DNAJB5	2.6994	5.00E-05	0.001089
VGF	2.69259	5.00E-05	0.001089
PPP1R1C	2.67988	5.00E-05	0.001089
ADAMTSL4	2.64898	5.00E-05	0.001089
ULBP2	2.64691	5.00E-05	0.001089
TMEM88	2.64381	5.00E-05	0.001089
IL6ST	2.6429	5.00E-05	0.001089
CDH9	2.64147	5.00E-05	0.001089
HDX	2.63438	5.00E-05	0.001089
LOC100507217	2.63168	5.00E-05	0.001089
FREM2	2.63111	5.00E-05	0.001089
ESR1	2.62524	5.00E-05	0.001089
C19orf66	2.61581	5.00E-05	0.001089
ITGA5	2.60903	5.00E-05	0.001089
IFI44L	2.60377	5.00E-05	0.001089
NFKB2	2.59888	5.00E-05	0.001089
HLA-A	2.59321	5.00E-05	0.001089
ADIRF	2.58978	0.0002	0.003428
IL11	2.58936	5.00E-05	0.001089
APOBEC3B	2.58875	5.00E-05	0.001089
LOC339166	2.58567	5.00E-05	0.001089
GPIHBP1	2.58433	5.00E-05	0.001089
GBP4	2.58192	5.00E-05	0.001089
EIF2AK2	2.57968	5.00E-05	0.001089
CTNNAL1	2.56487	5.00E-05	0.001089
TRIM21	2.55492	5.00E-05	0.001089
GIF	2.55337	0.00385	0.031636
OTOP2	2.55326	0.0009	0.010731
TAPBPL	2.5442	0.0071	0.049381
TMEM27	2.54257	5.00E-05	0.001089
TTC9B	2.54122	0.00025	0.004092
DLX2	2.53425	5.00E-05	0.001089

OR1F2P	2.52915	0.0006	0.008024
HIVEP2	2.52818	5.00E-05	0.001089
IL7	2.52746	5.00E-05	0.001089
PTPRH	2.52665	5.00E-05	0.001089
ABL2	2.5259	5.00E-05	0.001089
CMTM2	2.51765	0.0001	0.001954
ZSWIM4	2.51381	5.00E-05	0.001089
MEG9	2.50642	5.00E-05	0.001089
TSPYL2	2.50101	5.00E-05	0.001089
SPAG9	2.49418	5.00E-05	0.001089
CNTN6	2.49354	0.0001	0.001954
PPM1K	2.49287	5.00E-05	0.001089
IGF2	2.48941	5.00E-05	0.001089
IFIT5	2.48313	5.00E-05	0.001089
SP100	2.46777	5.00E-05	0.001089
ART4	2.46661	5.00E-05	0.001089
ARC	2.46215	5.00E-05	0.001089
NCOA7	2.45916	5.00E-05	0.001089
FLJ12334	2.45613	5.00E-05	0.001089
SCN3A	2.45384	5.00E-05	0.001089
CYP1A1	2.45104	5.00E-05	0.001089
NAV3	2.45049	5.00E-05	0.001089
NFKBIE	2.44792	5.00E-05	0.001089
C11orf91	2.44202	5.00E-05	0.001089
OR1F1	2.42491	5.00E-05	0.001089
SEMA3A	2.42463	5.00E-05	0.001089
TBX10	2.4187	5.00E-05	0.001089
AOX1	2.41851	5.00E-05	0.001089
SPRY2	2.41423	5.00E-05	0.001089
DUSP6	2.41175	5.00E-05	0.001089
TAP1	2.39958	5.00E-05	0.001089
LINC00152	2.39734	5.00E-05	0.001089
FOSL1	2.39411	5.00E-05	0.001089
SERTAD1	2.39353	5.00E-05	0.001089
AKAP12	2.39238	5.00E-05	0.001089
TGIF2	2.38861	0.0002	0.003428
BTN2A2	2.38586	5.00E-05	0.001089
ARHGAP40	2.38329	0.0002	0.003428
HCAR2	2.38231	5.00E-05	0.001089
HCP5	2.37764	5.00E-05	0.001089
NUDT8	2.37748	5.00E-05	0.001089
GYPE	2.37607	0.0017	0.017361
KLF6	2.37314	5.00E-05	0.001089
PTCHD2	2.37282	5.00E-05	0.001089
IL10RA	2.37094	5.00E-05	0.001089
HIP1R	2.3708	5.00E-05	0.001089

CSRNP1	2.35651	5.00E-05	0.001089
FST	2.35145	5.00E-05	0.001089
HOMER1	2.34937	5.00E-05	0.001089
VSTM1	2.33651	0.00025	0.004092
IL31RA	2.32823	5.00E-05	0.001089
DKK1	2.32285	5.00E-05	0.001089
LAMC2	2.32085	5.00E-05	0.001089
H3F3C	2.31916	5.00E-05	0.001089
PHLDA1	2.31113	5.00E-05	0.001089
NBEAP1	2.31083	0.0027	0.024344
TNFSF10	2.30863	0.0001	0.001954
TOP1P1	2.3067	5.00E-05	0.001089
JMJD1C	2.30348	5.00E-05	0.001089
PLCB4	2.30158	5.00E-05	0.001089
ARL5B	2.30108	5.00E-05	0.001089
BCL2A1	2.29987	5.00E-05	0.001089
NFKBIA	2.29981	5.00E-05	0.001089
AADAC	2.29756	5.00E-05	0.001089
FAM53C	2.29049	5.00E-05	0.001089
RELB	2.28661	5.00E-05	0.001089
ERO1LB	2.27891	5.00E-05	0.001089
DHRS9	2.27832	5.00E-05	0.001089
NR1D1	2.27358	5.00E-05	0.001089
PFKFB4	2.27347	5.00E-05	0.001089
MAP1LC3B	2.27241	5.00E-05	0.001089
CREBRF	2.26854	5.00E-05	0.001089
LDLRAD4	2.26437	5.00E-05	0.001089
FAM71F2	2.26162	5.00E-05	0.001089
UBA7	2.2584	5.00E-05	0.001089
LOC646498	2.25795	0.0031	0.026901
C17orf66	2.25762	5.00E-05	0.001089
CYP2J2	2.25466	0.0029	0.025597
ADAMTS19	2.25373	5.00E-05	0.001089
MCTP1	2.24708	5.00E-05	0.001089
IGF2BP2	2.24572	5.00E-05	0.001089
H1F0	2.24492	5.00E-05	0.001089
C16orf46	2.24267	5.00E-05	0.001089
ACSBG1	2.23737	5.00E-05	0.001089
STARD13	2.23187	5.00E-05	0.001089
RORB	2.22512	0.00035	0.005299
HIST1H3D	2.22024	5.00E-05	0.001089
CTGF	2.21974	5.00E-05	0.001089
NUAK2	2.21893	5.00E-05	0.001089
HLA-G	2.2099	0.00015	0.002736
MAP2K3	2.20267	5.00E-05	0.001089
PML	2.20248	5.00E-05	0.001089

APOL3	2.20145	0.0003	0.004684
LOC440461	2.19229	0.003	0.026176
SLC36A2	2.19063	5.00E-05	0.001089
HOXB4	2.18659	5.00E-05	0.001089
HIST1H2BF	2.18109	5.00E-05	0.001089
CCDC85B	2.17769	5.00E-05	0.001089
PER1	2.17655	5.00E-05	0.001089
HRH1	2.1732	5.00E-05	0.001089
IER2	2.16798	5.00E-05	0.001089
FAM151B	2.16607	5.00E-05	0.001089
HPCAL4	2.16373	0.0001	0.001954
PCK1	2.16058	0.00095	0.011119
LOC339894	2.16034	0.0002	0.003428
GIMAP2	2.1586	0.00375	0.031038
CRY1	2.14853	5.00E-05	0.001089
ETS1	2.14596	5.00E-05	0.001089
TRIM25	2.14281	5.00E-05	0.001089
DNAH17	2.13665	0.00025	0.004092
LOC100862671	2.13664	5.00E-05	0.001089
THEMIS2	2.13279	5.00E-05	0.001089
CEACAM20	2.13057	0.00125	0.013761
TRIM5	2.1305	5.00E-05	0.001089
SUCNR1	2.12886	0.00185	0.018595
NEDD4L	2.12296	5.00E-05	0.001089
C7orf63	2.11981	5.00E-05	0.001089
LOC285627	2.11737	5.00E-05	0.001089
CASQ1	2.11112	0.00045	0.006411
NEU1	2.10877	5.00E-05	0.001089
NFAT5	2.10831	5.00E-05	0.001089
PNRC1	2.10416	5.00E-05	0.001089
TCTE1	2.10282	0.00055	0.007519
PNPT1	2.10281	5.00E-05	0.001089
LOC100216546	2.09927	5.00E-05	0.001089
PCP4L1	2.09831	0.00355	0.029785
COL12A1	2.09125	5.00E-05	0.001089
RSRC2	2.08574	5.00E-05	0.001089
ADRB2	2.08549	5.00E-05	0.001089
KCNN2	2.08241	0.0001	0.001954
NPTX1	2.07907	5.00E-05	0.001089
HLA-C	2.07719	5.00E-05	0.001089
SORBS1	2.07717	5.00E-05	0.001089
TIMP3	2.07714	0.0003	0.004684
GPR26	2.07677	5.00E-05	0.001089
LOC100216545	2.0763	5.00E-05	0.001089
MMD2	2.07403	0.0001	0.001954
REL	2.06818	5.00E-05	0.001089

ZNF655	2.06687	5.00E-05	0.001089
TMEM198	2.06388	5.00E-05	0.001089
GPBP1	2.06282	5.00E-05	0.001089
GRK4	2.05946	0.0007	0.008891
SDCBP	2.05931	5.00E-05	0.001089
MTHFR	2.05613	5.00E-05	0.001089
DNAJB4	2.05451	5.00E-05	0.001089
XDH	2.05025	5.00E-05	0.001089
S100A2	2.04178	5.00E-05	0.001089
TP53INP2	2.03771	5.00E-05	0.001089
HIST1H2BH	2.03146	0.00065	0.008431
SAA2	2.03106	0.0068	0.048028
HAPLN3	2.02848	5.00E-05	0.001089
PATL2	2.02183	0.00085	0.010312
ABCB1	2.02175	0.0008	0.009825
CCNL1	2.02113	5.00E-05	0.001089
NFKB1	2.01981	5.00E-05	0.001089
PINLYP	2.00666	0.0012	0.013303
CDKN1C	2.00454	0.00055	0.007519
IFI35	2.00433	5.00E-05	0.001089
HAP1	2.00289	5.00E-05	0.001089
ARID4B	2.00041	5.00E-05	0.001089
CDR1	1.99922	0.0001	0.001954
FAM90A25P	1.99201	0.00635	0.045556
SLC16A12	1.99175	0.0001	0.001954
FRMD6	1.98949	5.00E-05	0.001089
TOP1	1.98618	5.00E-05	0.001089
RCAN1	1.98279	5.00E-05	0.001089
LINC00494	1.98107	0.00065	0.008431
HIST1H4E	1.98035	5.00E-05	0.001089
IFITM2	1.97731	5.00E-05	0.001089
H3F3B	1.97578	5.00E-05	0.001089
PCID2	1.97167	5.00E-05	0.001089
LAP3	1.96894	5.00E-05	0.001089
FAM19A3	1.96673	0.0033	0.028192
PLSCR2	1.96329	0.0008	0.009825
IGJ	1.95973	0.003	0.026176
ZNF556	1.9576	0.002	0.01979
DEF6	1.95713	0.00445	0.035135
PGF	1.9552	5.00E-05	0.001089
STK19	1.95439	0.00075	0.009355
TRIM69	1.95329	0.0019	0.019025
KPNA7	1.95309	5.00E-05	0.001089
TMEM86A	1.95256	0.0015	0.015773
TBX21	1.94985	0.00055	0.007519
GCAT	1.9478	5.00E-05	0.001089

LY6G6C	1.94575	0.0018	0.018195
DUSP5	1.94367	5.00E-05	0.001089
GLS	1.94151	5.00E-05	0.001089
LOC339807	1.94042	0.00035	0.005299
TFPI	1.94036	5.00E-05	0.001089
KBTBD13	1.93952	0.0011	0.012454
HSPB8	1.93771	5.00E-05	0.001089
ELL2	1.936	5.00E-05	0.001089
TRIM38	1.9345	5.00E-05	0.001089
SMTNL1	1.93317	0.0003	0.004684
ACO1	1.93008	5.00E-05	0.001089
LAMB2P1	1.92767	0.0001	0.001954
APOF	1.92724	0.0001	0.001954
RSPRY1	1.92668	5.00E-05	0.001089
CLPS	1.92486	0.00575	0.04224
LATS2	1.92377	5.00E-05	0.001089
MSANTD3	1.91618	5.00E-05	0.001089
PLAUR	1.91588	5.00E-05	0.001089
AZI2	1.91527	5.00E-05	0.001089
HIST1H4H	1.91472	5.00E-05	0.001089
MEG8	1.91448	0.00325	0.027854
AGTR1	1.91292	0.0007	0.008891
OLR1	1.91094	0.0002	0.003428
CCDC11	1.91005	5.00E-05	0.001089
AMHR2	1.90975	0.00045	0.006411
DNAH2	1.90804	5.00E-05	0.001089
KLF4	1.90795	5.00E-05	0.001089
CA13	1.90291	5.00E-05	0.001089
CLK1	1.90291	5.00E-05	0.001089
GRHL2	1.90216	0.0003	0.004684
CLDN6	1.90166	0.0004	0.005861
UCN2	1.90134	5.00E-05	0.001089
MAP1LC3A	1.89741	5.00E-05	0.001089
SLC30A7	1.89402	5.00E-05	0.001089
TRIM14	1.89249	5.00E-05	0.001089
WHAMM	1.89175	5.00E-05	0.001089
SLC6A13	1.89031	0.00065	0.008431
APLF	1.88369	5.00E-05	0.001089
DCHS1	1.88317	5.00E-05	0.001089
SELL	1.88163	0.00135	0.014531
EBI3	1.88157	0.0011	0.012454
SEMA7A	1.87974	5.00E-05	0.001089
COL1A2	1.87623	0.0013	0.014165
ANXA5	1.87382	5.00E-05	0.001089
ACHE	1.87313	0.0001	0.001954
KLF10	1.87284	5.00E-05	0.001089

IRF8	1.86942	0.0001	0.001954
IL1A	1.86159	0.0002	0.003428
TRA2A	1.85937	5.00E-05	0.001089
LOC100288181	1.85404	5.00E-05	0.001089
ACER2	1.8527	5.00E-05	0.001089
RASGRP3	1.8486	5.00E-05	0.001089
LOC541471	1.84817	5.00E-05	0.001089
CYP3A5	1.8452	5.00E-05	0.001089
LAMB3	1.84445	5.00E-05	0.001089
WDR26	1.84344	5.00E-05	0.001089
RUNX1	1.84321	5.00E-05	0.001089
TKTL1	1.84293	0.0008	0.009825
PRDM1	1.84235	5.00E-05	0.001089
C2CD4A	1.83986	0.00025	0.004092
TLR3	1.8397	5.00E-05	0.001089
C11orf68	1.83518	5.00E-05	0.001089
PLAGL1	1.83488	5.00E-05	0.001089
KLF2	1.83447	5.00E-05	0.001089
LOC284581	1.83221	5.00E-05	0.001089
HSH2D	1.83187	5.00E-05	0.001089
HCLS1	1.82956	0.00045	0.006411
LRRC32	1.82777	0.00075	0.009355
KDM6A	1.82742	5.00E-05	0.001089
SYNE1	1.8259	5.00E-05	0.001089
THEG	1.8236	0.00095	0.011119
USP53	1.82206	5.00E-05	0.001089
HIST1H2BK	1.82185	5.00E-05	0.001089
FNIP1	1.82009	5.00E-05	0.001089
AP3S1	1.819	5.00E-05	0.001089
SLCO1B7	1.81747	0.0001	0.001954
GALR3	1.81741	0.0022	0.021101
FAM133DP	1.81574	5.00E-05	0.001089
NR1D2	1.81574	5.00E-05	0.001089
H1FX	1.81429	5.00E-05	0.001089
LGALS3BP	1.81276	5.00E-05	0.001089
C6orf25	1.80969	5.00E-05	0.001089
ROCK1P1	1.80797	0.0003	0.004684
TAS2R42	1.80648	0.00425	0.034028
NLGN3	1.80488	5.00E-05	0.001089
LOC201651	1.80287	0.0003	0.004684
RIOK3	1.80218	5.00E-05	0.001089
TRIP12	1.80218	5.00E-05	0.001089
CDKL4	1.80185	0.00335	0.028542
AHR	1.8007	5.00E-05	0.001089
LOC100506668	1.79774	0.00045	0.006411
SPRED2	1.79461	5.00E-05	0.001089

PHLDA2	1.79232	5.00E-05	0.001089
TRIML2	1.79217	5.00E-05	0.001089
BMP2	1.79212	0.0001	0.001954
C8orf42	1.79048	5.00E-05	0.001089
LOC643723	1.78666	0.0012	0.013303
DUSP1	1.78619	5.00E-05	0.001089
CSF3	1.78531	0.00055	0.007519
GCNT1	1.78428	5.00E-05	0.001089
ZSCAN12P1	1.78133	5.00E-05	0.001089
ETV7	1.77971	0.00065	0.008431
BEST3	1.7781	0.00015	0.002736
MIR100HG	1.77557	5.00E-05	0.001089
ZNF335	1.77387	5.00E-05	0.001089
TMEM95	1.77204	0.00135	0.014531
LAMTOR3	1.77063	5.00E-05	0.001089
ING1	1.76945	5.00E-05	0.001089
KDM5B	1.7691	5.00E-05	0.001089
KLHL4	1.76639	5.00E-05	0.001089
SOCS3	1.76181	5.00E-05	0.001089
NFKBIB	1.7612	5.00E-05	0.001089
C8orf46	1.75999	0.0003	0.004684
SYNJ1	1.75975	5.00E-05	0.001089
PROX1	1.75881	0.00015	0.002736
FNDC7	1.75351	0.00115	0.012865
GPR3	1.75222	0.0001	0.001954
EXT1	1.75174	5.00E-05	0.001089
FYB	1.75134	5.00E-05	0.001089
RAB30	1.74992	5.00E-05	0.001089
MPZL3	1.74817	0.0004	0.005861
CSRP2	1.74763	5.00E-05	0.001089
ALDH8A1	1.7358	0.00015	0.002736
MAFA	1.73573	0.00035	0.005299
SERPINB9	1.72876	5.00E-05	0.001089
NEURL3	1.72801	5.00E-05	0.001089
IFRD1	1.72754	5.00E-05	0.001089
C3	1.72633	5.00E-05	0.001089
LIMA1	1.72513	5.00E-05	0.001089
SHISA2	1.72413	0.00035	0.005299
FMR1	1.72186	5.00E-05	0.001089
RHEBL1	1.72172	5.00E-05	0.001089
HLA-DOB	1.72148	0.00215	0.020747
HES4	1.7204	5.00E-05	0.001089
TNC	1.7183	0.0002	0.003428
LOC100287846	1.71811	5.00E-05	0.001089
GP6	1.71495	5.00E-05	0.001089
BRAF	1.7135	5.00E-05	0.001089

TSC22D2	1.71332	5.00E-05	0.001089
SP140L	1.71283	0.0001	0.001954
CD59	1.71261	5.00E-05	0.001089
SAMD4A	1.71223	5.00E-05	0.001089
GORAB	1.71211	5.00E-05	0.001089
SLCO1C1	1.71204	0.0004	0.005861
HIST1H4J	1.71007	5.00E-05	0.001089
SLCO1A2	1.70922	0.0016	0.016584
EEA1	1.70829	5.00E-05	0.001089
BCL2L12	1.70733	0.0013	0.014165
RASAL2	1.70457	5.00E-05	0.001089
C5orf56	1.70341	0.0006	0.008024
SMURF2	1.70336	5.00E-05	0.001089
LOC100289187	1.7023	0.0007	0.008891
FAIM3	1.69945	0.0025	0.023036
CRISPLD2	1.6991	5.00E-05	0.001089
TFPI2	1.69767	5.00E-05	0.001089
NBR1	1.69749	5.00E-05	0.001089
CHD2	1.69476	5.00E-05	0.001089
LOC100128822	1.69384	0.00025	0.004092
TSC22D1	1.68677	5.00E-05	0.001089
HERC4	1.68567	5.00E-05	0.001089
USP43	1.68303	5.00E-05	0.001089
SERPINB8	1.68268	5.00E-05	0.001089
MASTL	1.68179	5.00E-05	0.001089
AP1B1P1	1.67807	0.0037	0.030688
DDX26B	1.67733	0.00075	0.009355
PGM5	1.66994	0.00205	0.020098
GTF2B	1.66675	5.00E-05	0.001089
SGMS2	1.66468	5.00E-05	0.001089
ATP6V1D	1.66461	5.00E-05	0.001089
BAZ1A	1.66327	5.00E-05	0.001089
PIP5K1A	1.66294	5.00E-05	0.001089
RBM24	1.66194	5.00E-05	0.001089
CPM	1.66179	5.00E-05	0.001089
LIPG	1.66117	0.00045	0.006411
PCDH10	1.65996	5.00E-05	0.001089
MAFB	1.65713	0.00105	0.012007
RCBTB2	1.65231	0.0001	0.001954
RANBP3L	1.64767	0.00355	0.029785
ZCCHC6	1.64666	5.00E-05	0.001089
CITED2	1.64584	5.00E-05	0.001089
DPY19L2P2	1.64326	5.00E-05	0.001089
CXCL3	1.64115	5.00E-05	0.001089
PIWIL2	1.64041	0.00015	0.002736
LOC283663	1.63462	0.0001	0.001954

GLCCI1	1.6335	5.00E-05	0.001089
TNFRSF10B	1.63073	5.00E-05	0.001089
SOWAHB	1.63038	0.00415	0.033547
SCYL2	1.62862	5.00E-05	0.001089
FOSL2	1.62829	5.00E-05	0.001089
UBE2L6	1.62538	5.00E-05	0.001089
IMPG2	1.62431	0.00055	0.007519
LINC00507	1.62328	0.00685	0.048253
ING3	1.62273	5.00E-05	0.001089
BACH2	1.62258	0.0002	0.003428
PARD6G	1.62061	0.00015	0.002736
EPC1	1.6192	5.00E-05	0.001089
PTHLH	1.61771	5.00E-05	0.001089
BTBD19	1.61728	0.0033	0.028192
NR4A2	1.61432	5.00E-05	0.001089
SAT1	1.61251	5.00E-05	0.001089
CPEB2	1.6119	5.00E-05	0.001089
MT2A	1.60968	0.00035	0.005299
HAS2	1.60966	0.00165	0.017002
PRKD2	1.60834	5.00E-05	0.001089
ANKRD33B	1.60689	5.00E-05	0.001089
CDK7	1.60476	5.00E-05	0.001089
ROPN1L	1.60335	0.00275	0.024628
DLL1	1.60267	0.00075	0.009355
SIRT1	1.6003	5.00E-05	0.001089
GPATCH2L	1.5995	5.00E-05	0.001089
DLL4	1.59932	0.0004	0.005861
ABCC11	1.59798	0.0011	0.012454
NR1H4	1.59529	0.0003	0.004684
KIAA1045	1.59169	0.00095	0.011119
ADAR	1.58865	5.00E-05	0.001089
CATSPERG	1.58837	0.0002	0.003428
SETX	1.58815	5.00E-05	0.001089
SLCO1B3	1.58781	5.00E-05	0.001089
KBTBD8	1.58738	5.00E-05	0.001089
HBP1	1.58596	0.00415	0.033547
SLC8A2	1.58394	0.0006	0.008024
PIGA	1.58388	5.00E-05	0.001089
ITGAV	1.5803	5.00E-05	0.001089
NGFR	1.58029	0.0017	0.017361
LOC392364	1.57767	0.00275	0.024628
TNFRSF10D	1.57748	5.00E-05	0.001089
MFSD2B	1.57471	0.00075	0.009355
MITF	1.57465	5.00E-05	0.001089
ASB2	1.5737	0.0038	0.031338
FBXO33	1.57352	5.00E-05	0.001089

BBC3	1.57253	5.00E-05	0.001089
AZIN1	1.57048	5.00E-05	0.001089
PHF11	1.5695	0.0001	0.001954
RAB5A	1.56761	5.00E-05	0.001089
GPD1	1.56684	0.00025	0.004092
ELF1	1.56683	5.00E-05	0.001089
CLK4	1.56523	5.00E-05	0.001089
YTHDC1	1.56369	5.00E-05	0.001089
SLC6A12	1.56198	0.00225	0.021413
KCTD11	1.56073	5.00E-05	0.001089
CXCL2	1.56023	5.00E-05	0.001089
MMP25	1.5571	0.00045	0.006411
MICALCL	1.55639	0.00145	0.015348
CHEK2	1.55566	5.00E-05	0.001089
RELT	1.55552	5.00E-05	0.001089
NSUN6	1.55419	0.0001	0.001954
ADAP1	1.55217	0.0003	0.004684
CSDAP1	1.55211	0.00015	0.002736
C7orf71	1.551	0.0059	0.043143
SLC17A7	1.55015	0.00205	0.020098
LETM2	1.55002	0.00015	0.002736
HIST2H4A	1.54856	0.0001	0.001954
GADD45A	1.54789	5.00E-05	0.001089
ABCA5	1.5454	0.00015	0.002736
KLHL15	1.54298	5.00E-05	0.001089
KIAA1217	1.54017	5.00E-05	0.001089
MYPN	1.53603	5.00E-05	0.001089
SLCO1B1	1.53506	0.0016	0.016584
CD55	1.5344	5.00E-05	0.001089
NEXN	1.53411	0.0002	0.003428
TNIP2	1.53072	5.00E-05	0.001089
LOC400027	1.53006	5.00E-05	0.001089
USP16	1.52977	5.00E-05	0.001089
MAP1LC3B2	1.52486	0.00495	0.038212
ANKRD20A19P	1.52434	0.0004	0.005861
ALCAM	1.52408	5.00E-05	0.001089
RRAD	1.5235	0.00645	0.046066
NMI	1.52307	0.00025	0.004092
LINC00854	1.5224	0.00085	0.010312
VEGFC	1.51784	5.00E-05	0.001089
MITD1	1.51713	5.00E-05	0.001089
CORO2B	1.51637	0.0006	0.008024
SLC4A7	1.51572	5.00E-05	0.001089
BLZF1	1.51496	5.00E-05	0.001089
CDKN2AIP	1.51424	5.00E-05	0.001089
MATN1-AS1	1.51306	0.00015	0.002736

IMPA1	1.51203	5.00E-05	0.001089
EREG	1.51047	0.0001	0.001954
ARF4	1.50973	5.00E-05	0.001089
MYH15	1.50952	0.0005	0.006979
LRGUK	1.50469	0.0026	0.023711
GCH1	1.50293	0.00025	0.004092
CRABP2	1.49952	0.00095	0.011119
KATNA1	1.49836	0.00015	0.002736
BIRC2	1.49802	5.00E-05	0.001089
YOD1	1.49311	5.00E-05	0.001089
TLE4	1.49257	5.00E-05	0.001089
SRSF3	1.49187	5.00E-05	0.001089
MEG3	1.49179	0.0006	0.008024
PRDM8	1.49068	0.00195	0.01938
HSD17B7P2	1.49007	0.00025	0.004092
RBM39	1.48683	5.00E-05	0.001089
PPP2R3C	1.48592	5.00E-05	0.001089
SOCS2	1.48582	5.00E-05	0.001089
CASP3	1.48459	5.00E-05	0.001089
DCAF8L2	1.48379	0.00635	0.045556
HIST1H3E	1.48378	5.00E-05	0.001089
GRIK5	1.48345	0.0009	0.010731
GTPBP1	1.48344	5.00E-05	0.001089
DNAJC27	1.48314	5.00E-05	0.001089
ELOVL7	1.48234	5.00E-05	0.001089
INO80D	1.48129	5.00E-05	0.001089
XRN1	1.48073	5.00E-05	0.001089
ALOX15	1.47945	0.00525	0.039739
GDAP1	1.4786	5.00E-05	0.001089
UTY	1.47747	5.00E-05	0.001089
RPL13AP20	1.47496	0.0001	0.001954
NIM1	1.4738	0.00105	0.012007
CYFIP1	1.47337	5.00E-05	0.001089
VPS37B	1.47123	5.00E-05	0.001089
NMT2	1.46963	5.00E-05	0.001089
LOX	1.46789	0.00025	0.004092
AXL	1.46592	5.00E-05	0.001089
YY1AP1	1.46575	5.00E-05	0.001089
STX3	1.46538	5.00E-05	0.001089
CHGB	1.46419	0.00045	0.006411
PPIL4	1.46255	0.0001	0.001954
SEC24A	1.46214	5.00E-05	0.001089
ZMYM5	1.45997	0.00035	0.005299
TRIM26	1.45911	5.00E-05	0.001089
RABGEF1	1.45882	5.00E-05	0.001089
ZBTB10	1.45866	5.00E-05	0.001089

KIAA1033	1.45302	5.00E-05	0.001089
YPEL4	1.45275	5.00E-05	0.001089
HOXD10	1.45089	0.00075	0.009355
WSB1	1.45022	5.00E-05	0.001089
UGCG	1.44906	5.00E-05	0.001089
DDX3X	1.44903	0.00015	0.002736
SMC4	1.44885	5.00E-05	0.001089
DDX10	1.44723	5.00E-05	0.001089
STX11	1.44663	0.00035	0.005299
HIST2H4B	1.446	5.00E-05	0.001089
COL7A1	1.44367	0.0001	0.001954
MARCH4	1.44343	5.00E-05	0.001089
THAP1	1.4419	5.00E-05	0.001089
HIST2H2BA	1.44179	0.0008	0.009825
AOC3	1.43987	0.00015	0.002736
DCAF8	1.43954	5.00E-05	0.001089
ZNF764	1.43841	0.0001	0.001954
HELQ	1.43742	5.00E-05	0.001089
SLC25A2	1.43736	0.00395	0.032241
FZD4	1.4363	5.00E-05	0.001089
RABGGTB	1.4335	5.00E-05	0.001089
CEP135	1.43309	5.00E-05	0.001089
P4HA1	1.43206	5.00E-05	0.001089
MTRNR2L8	1.43158	0.0066	0.046885
RPL21P44	1.43095	0.0012	0.013303
B3GALNT2	1.42889	0.0004	0.005861
NPC1	1.42853	5.00E-05	0.001089
RYBP	1.42744	5.00E-05	0.001089
ZNF474	1.42664	0.00075	0.009355
OXTR	1.42615	5.00E-05	0.001089
HSPH1	1.42551	5.00E-05	0.001089
KIF20B	1.42535	5.00E-05	0.001089
AKT1S1	1.42523	5.00E-05	0.001089
ANKRD13A	1.42512	5.00E-05	0.001089
POTEM	1.4235	0.0004	0.005861
LINC00472	1.41882	5.00E-05	0.001089
LYST	1.41725	0.0001	0.001954
SLC41A1	1.41595	5.00E-05	0.001089
DCUN1D3	1.41452	5.00E-05	0.001089
EPHA4	1.41448	0.0005	0.006979
CEACAM22P	1.41294	0.001	0.011594
C10orf118	1.41278	5.00E-05	0.001089
SMARCA5	1.41273	5.00E-05	0.001089
HIST1H4K	1.41099	0.0002	0.003428
PPEF1	1.41097	0.0024	0.022413
SPRY4	1.41067	5.00E-05	0.001089

SMNDC1	1.40838	0.0001	0.001954
CENPE	1.40831	5.00E-05	0.001089
HIST3H2BB	1.40757	5.00E-05	0.001089
BCL6	1.40577	5.00E-05	0.001089
LMO7	1.40552	5.00E-05	0.001089
RGS2	1.40527	0.0001	0.001954
IDI1	1.40412	0.00095	0.011119
DDX3Y	1.40389	5.00E-05	0.001089
C21orf91	1.40346	0.00035	0.005299
IL17RE	1.4033	0.0046	0.036087
PGM5P2	1.40278	0.0002	0.003428
GALR2	1.40264	0.00585	0.042856
ANGPTL4	1.40247	5.00E-05	0.001089
RCOR1	1.4022	5.00E-05	0.001089
SNHG8	1.4012	0.0002	0.003428
EFNA1	1.4009	5.00E-05	0.001089
HIST1H1C	1.40065	0.00045	0.006411
LACC1	1.39994	0.00235	0.022088
STX5	1.39764	5.00E-05	0.001089
NCALD	1.39609	0.001	0.011594
UNK	1.39592	0.00225	0.021413
HIST1H2BC	1.39504	0.0001	0.001954
PRPF38B	1.39461	5.00E-05	0.001089
RHBDF1	1.39117	5.00E-05	0.001089
BTAF1	1.39033	5.00E-05	0.001089
POLR2A	1.38973	5.00E-05	0.001089
NOC3L	1.38972	5.00E-05	0.001089
MLL5	1.3889	5.00E-05	0.001089
TAF13	1.38633	0.00075	0.009355
NOS2	1.38522	0.0031	0.026901
AREG	1.38474	0.0001	0.001954
FBXO38	1.38448	5.00E-05	0.001089
ANO6	1.38267	5.00E-05	0.001089
NXF1	1.38136	0.00015	0.002736
DUSP16	1.3789	5.00E-05	0.001089
PTBP2	1.37857	5.00E-05	0.001089
HELZ	1.3778	5.00E-05	0.001089
BCLAF1	1.37724	5.00E-05	0.001089
LINC00346	1.3749	0.0004	0.005861
TJAP1	1.37441	0.00025	0.004092
PLAG1	1.37434	0.00085	0.010312
SDC4	1.3738	5.00E-05	0.001089
METTL21D	1.37285	0.0002	0.003428
SNPH	1.37265	0.0001	0.001954
NIPBL	1.3719	0.0002	0.003428
ZC3H11A	1.36935	0.0012	0.013303

CCNT2	1.36902	5.00E-05	0.001089
HCG27	1.36344	0.00085	0.010312
MYD88	1.36264	0.00015	0.002736
DNAJC25	1.36144	0.0042	0.033797
KIAA1199	1.36127	0.0005	0.006979
ZNF701	1.35865	0.0052	0.039473
FAM214A	1.35834	5.00E-05	0.001089
DIP2C	1.35623	0.0002	0.003428
TRAPPC6B	1.35606	5.00E-05	0.001089
ELMSAN1	1.35591	0.00045	0.006411
RNMT	1.35555	5.00E-05	0.001089
IL15	1.35523	0.0003	0.004684
EBF4	1.35506	0.0025	0.023036
NSRP1	1.35481	0.0001	0.001954
C4orf32	1.35106	0.0051	0.038881
C1orf141	1.35072	0.007	0.04892
KDSR	1.34717	0.0001	0.001954
PLEKHO2	1.34557	0.00015	0.002736
CCDC174	1.34424	5.00E-05	0.001089
RLF	1.34208	5.00E-05	0.001089
SEC23B	1.34189	5.00E-05	0.001089
G2E3	1.34155	0.00015	0.002736
MORC3	1.34086	5.00E-05	0.001089
LINC00857	1.34051	0.0001	0.001954
CEP95	1.33946	5.00E-05	0.001089
FAM46B	1.33907	0.00025	0.004092
USE1	1.33747	0.00055	0.007519
ZUFSP	1.33658	5.00E-05	0.001089
ARL13B	1.33549	0.00025	0.004092
CLCN4	1.33324	0.0003	0.004684
GPRC5A	1.33053	5.00E-05	0.001089
ZNF697	1.33049	0.0002	0.003428
INTS6	1.33042	0.0002	0.003428
ADPRM	1.32849	0.00015	0.002736
TOR1AIP2	1.32841	0.0001	0.001954
MIER1	1.32841	0.0003	0.004684
ATG12	1.32799	0.0001	0.001954
C7orf53	1.32784	0.00105	0.012007
CRSP8P	1.32774	0.00195	0.01938
C3orf35	1.32691	0.00275	0.024628
BCL3	1.32671	0.0003	0.004684
LOC100506305	1.32638	0.00385	0.031636
OR13H1	1.32491	0.00515	0.039205
PNPLA8	1.32405	0.00045	0.006411
CWC22	1.32264	0.0001	0.001954
NFE2L3	1.32213	0.00015	0.002736

BTG1	1.32035	0.00015	0.002736
NFE2	1.32009	0.0072	0.049859
EPPK1	1.31954	0.0017	0.017361
GULP1	1.31917	0.0001	0.001954
ARNTL2	1.3191	0.0001	0.001954
MYEF2	1.3188	0.00015	0.002736
SAV1	1.3183	0.0001	0.001954
NT5C3	1.31564	0.00025	0.004092
ARAP2	1.31306	0.0002	0.003428
LOC440896	1.31262	0.00505	0.038666
JUND	1.31023	0.00015	0.002736
OSCP1	1.31002	0.00275	0.024628
C15orf48	1.30991	0.00565	0.041892
BTG3	1.30906	0.00025	0.004092
FOXO3	1.30904	5.00E-05	0.001089
SRSF5	1.30771	0.00025	0.004092
ABAT	1.30703	0.00385	0.031636
BCL10	1.30676	0.0003	0.004684
KIF18A	1.30635	0.0001	0.001954
CCDC117	1.30526	0.0001	0.001954
SAR1A	1.30317	5.00E-05	0.001089
KYNU	1.30272	0.00015	0.002736
ARSJ	1.30265	0.0001	0.001954
PLOD2	1.30263	5.00E-05	0.001089
BDP1	1.30161	0.0001	0.001954
MTMR3	1.3014	0.0001	0.001954
AFF4	1.29996	0.0001	0.001954
RAB9A	1.29775	5.00E-05	0.001089
CUL3	1.29745	0.00065	0.008431
SUZ12P1	1.29621	0.00475	0.037027
FAM126B	1.29335	0.0001	0.001954
LOC338817	1.29274	0.0016	0.016584
SELT	1.29264	0.00025	0.004092
AASS	1.29156	0.00225	0.021413
MED13	1.29143	5.00E-05	0.001089
VAV1	1.29093	0.0007	0.008891
HECA	1.29061	0.00065	0.008431
TRA2B	1.2879	5.00E-05	0.001089
NCEH1	1.2862	5.00E-05	0.001089
BRWD3	1.28412	0.0001	0.001954
TRIB1	1.28389	0.0001	0.001954
HIST1H4C	1.28388	5.00E-05	0.001089
DDX5	1.28213	0.00015	0.002736
CHRNB2	1.28163	0.00015	0.002736
CCNH	1.28118	5.00E-05	0.001089
BZW1	1.27948	0.0008	0.009825

VEZT	1.27894	5.00E-05	0.001089
MLL3	1.27863	5.00E-05	0.001089
JAK2	1.27684	0.00015	0.002736
ERCC6L2	1.27584	0.00045	0.006411
NFYB	1.27446	0.0004	0.005861
ZBTB43	1.27395	5.00E-05	0.001089
MAP4K5	1.27309	0.0002	0.003428
STRN3	1.27281	0.001	0.011594
KCNV1	1.27218	0.0033	0.028192
RNF10	1.27165	0.00015	0.002736
ATAD2B	1.271	0.0001	0.001954
GBF1	1.27065	0.0003	0.004684
DENND4A	1.26931	0.0004	0.005861
ZNF165	1.26906	0.00075	0.009355
TTC27	1.26858	0.00015	0.002736
LDLR	1.26698	0.0003	0.004684
RCHY1	1.26685	0.00045	0.006411
LOC441155	1.26635	0.00095	0.011119
ZSWIM6	1.26607	5.00E-05	0.001089
CT45A5	1.26506	0.00275	0.024628
TAF1D	1.26487	0.00025	0.004092
ARIH1	1.26392	5.00E-05	0.001089
FTSJD2	1.26322	0.0002	0.003428
CHIC2	1.26301	0.0009	0.010731
FAM222A	1.26283	0.0013	0.014165
CDKN2C	1.26171	0.00135	0.014531
NLRC4	1.26166	0.003	0.026176
FER	1.26045	0.0004	0.005861
MKLN1	1.25995	0.00015	0.002736
IFT57	1.25978	0.00065	0.008431
FRS2	1.25724	0.0001	0.001954
HIAT1	1.25628	5.00E-05	0.001089
SLMAP	1.25343	0.0002	0.003428
SLC12A4	1.25216	0.0021	0.0204
OFD1	1.2512	5.00E-05	0.001089
ZKSCAN1	1.25104	5.00E-05	0.001089
C6orf141	1.25059	0.0003	0.004684
SEMA3C	1.25046	0.0001	0.001954
LOC100287314	1.24931	0.0004	0.005861
SNX16	1.24873	0.00375	0.031038
DIABLO	1.24828	0.0001	0.001954
TM9SF3	1.24777	0.0003	0.004684
SCFD1	1.24622	0.00045	0.006411
NFKBID	1.24386	0.0005	0.006979
PELI1	1.24306	0.0004	0.005861
COQ10B	1.24267	0.0001	0.001954

SREK1	1.24001	0.00205	0.020098
NMNAT2	1.23957	0.0011	0.012454
BHLHE40	1.23952	0.0013	0.014165
LIG4	1.23934	0.00025	0.004092
ZDHHC17	1.23918	0.00055	0.007519
SBDS	1.23892	0.0003	0.004684
LOC100133612	1.23891	0.0061	0.044281
DNAJA1	1.23826	0.0002	0.003428
AP1G1	1.23813	0.00015	0.002736
TTF1	1.23554	0.0001	0.001954
HNRPLL	1.23512	0.0002	0.003428
SON	1.23231	0.0002	0.003428
PPP2R2A	1.2294	0.0007	0.008891
KHNYN	1.2291	0.0014	0.014968
MAK	1.22887	0.00265	0.024071
ATP1B2	1.22856	0.0052	0.039473
WTAP	1.22808	0.0001	0.001954
MYSM1	1.22589	0.0003	0.004684
HIST1H2AC	1.22485	0.00095	0.011119
NEDD4	1.22475	0.0001	0.001954
RBM5	1.22469	0.00025	0.004092
LRRC8E	1.22464	0.00065	0.008431
RND1	1.22383	0.00025	0.004092
C1orf52	1.22382	0.0007	0.008891
CD2AP	1.22381	0.00035	0.005299
RTN4	1.22298	0.0007	0.008891
DHX36	1.2204	0.0006	0.008024
TEAD1	1.21714	0.0002	0.003428
FRRS1	1.21473	0.00155	0.016224
ZNF670	1.21408	0.00295	0.025924
LINC00707	1.21379	0.0042	0.033797
CPEB3	1.21374	0.00245	0.022773
C1orf63	1.21221	0.00045	0.006411
UBR1	1.21201	0.0002	0.003428
HNRNPU	1.21179	0.00055	0.007519
BNIP3	1.2116	0.0002	0.003428
HIVEP1	1.21151	0.0001	0.001954
THOC6	1.21125	0.0071	0.049381
TAPT1	1.21101	0.00035	0.005299
ZBED5	1.21008	0.0002	0.003428
TET2	1.20961	0.0001	0.001954
ZFYVE1	1.20813	0.0007	0.008891
DSEL	1.206	0.003	0.026176
FAM91A1	1.20455	0.0003	0.004684
CHMP4C	1.20404	0.0003	0.004684
TNFRSF12A	1.20348	0.0004	0.005861

HIPK3	1.20328	0.0002	0.003428
ARID4A	1.20307	0.0006	0.008024
LRIF1	1.2029	0.00045	0.006411
CD22	1.20164	0.0049	0.0379
ERAP2	1.19937	0.0023	0.021759
SP2	1.19933	0.0004	0.005861
FAM200A	1.19822	0.0004	0.005861
SLC2A12	1.1971	0.0041	0.033278
SPOCK1	1.19641	0.0056	0.041637
CELSR3	1.19624	0.0009	0.010731
ZBTB20	1.19571	0.0002	0.003428
UBE2D1	1.19525	0.00125	0.013761
SHOC2	1.19522	0.0006	0.008024
ZNF267	1.19468	0.00025	0.004092
PAF1	1.19325	0.00015	0.002736
RAP1B	1.19158	0.00065	0.008431
ZNF107	1.19009	0.0009	0.010731
HMMR	1.19007	0.0007	0.008891
EP300	1.1892	0.00035	0.005299
CREBBP	1.18821	0.0001	0.001954
FBP2	1.18819	0.00695	0.048678
CCNC	1.18745	0.00085	0.010312
THUMPD2	1.18613	0.00035	0.005299
NF1	1.18475	0.0013	0.014165
MAP4K3	1.18461	0.00035	0.005299
PPAP2B	1.18438	0.00015	0.002736
GEM	1.18408	0.0002	0.003428
GPCPD1	1.18391	0.0003	0.004684
C6orf62	1.18354	0.00155	0.016224
NAALAD2	1.18306	0.003	0.026176
RAB28	1.18257	0.0015	0.015773
PRPF38A	1.1824	0.00035	0.005299
GNG4	1.18146	0.0027	0.024344
UPP1	1.1802	0.0001	0.001954
MYLIP	1.179	0.0001	0.001954
RICTOR	1.17847	0.00055	0.007519
ANKRD42	1.17839	0.0014	0.014968
FERMT2	1.17777	0.0006	0.008024
HSCB	1.17664	0.001	0.011594
ADAM9	1.17618	0.00035	0.005299
ARL4A	1.17574	0.0004	0.005861
LOC100506233	1.17463	0.00315	0.027261
ENSA	1.17383	0.0021	0.0204
TANK	1.17347	0.0027	0.024344
ZNF92	1.17292	0.00115	0.012865
TUFT1	1.17208	0.00045	0.006411

STK38L	1.172	0.0007	0.008891
MAP4K4	1.1718	0.00025	0.004092
UBE2H	1.16869	0.00035	0.005299
YIPF4	1.16784	0.0029	0.025597
DNAJB2	1.1678	0.00105	0.012007
TYW5	1.16641	0.006	0.043714
HES1	1.16624	0.0004	0.005861
GFRA1	1.16611	0.0039	0.031948
CHMP2B	1.16504	0.0013	0.014165
UBE2D3	1.16367	0.00035	0.005299
TP53BP2	1.16287	0.0001	0.001954
RP2	1.16195	0.00055	0.007519
GATA6	1.1619	0.00145	0.015348
CIC	1.16146	0.0006	0.008024
HIST1H4L	1.1589	0.0014	0.014968
CSNK1A1L	1.15843	0.0017	0.017361
TMEM106A	1.15799	0.00075	0.009355
DCBLD2	1.1567	0.00045	0.006411
MTVR2	1.15599	0.0057	0.042028
ZDBF2	1.15541	5.00E-05	0.001089
USP9X	1.15511	0.00065	0.008431
MIRLET7DHG	1.15366	0.0009	0.010731
EIF5	1.15067	0.0008	0.009825
KCNJ14	1.15004	0.0004	0.005861
TBC1D23	1.14972	0.0004	0.005861
ARL8B	1.14621	0.00085	0.010312
ATF2	1.14583	0.00165	0.017002
SPATS2L	1.14339	0.0004	0.005861
NABP1	1.14304	0.00065	0.008431
USP8	1.1412	0.0009	0.010731
KDM5D	1.13968	0.00055	0.007519
SLC12A7	1.13753	0.00115	0.012865
CHORDC1	1.13711	0.0007	0.008891
ARID3B	1.13645	0.0006	0.008024
PLEK2	1.13455	0.00555	0.041342
PIK3C2A	1.13355	0.0006	0.008024
FAM193A	1.13195	0.00045	0.006411
CNTN2	1.13193	0.0054	0.040509
C14orf28	1.13113	0.0048	0.037289
DGUOK	1.12904	0.00045	0.006411
ZNF207	1.12896	0.0004	0.005861
SLU7	1.12863	0.0003	0.004684
SNAP23	1.12832	0.00275	0.024628
ZNF274	1.12807	0.00045	0.006411
ZFAND3	1.12797	0.00075	0.009355
ANKRD20A5P	1.1274	0.0043	0.034308

HELB	1.12729	0.00135	0.014531
PDE10A	1.12659	0.0006	0.008024
CDC73	1.12618	0.00195	0.01938
DRAP1	1.12594	0.0013	0.014165
UBN2	1.12524	0.0003	0.004684
TGFB2	1.12512	0.00045	0.006411
LOC91948	1.12372	0.0021	0.0204
CD164	1.12351	0.0005	0.006979
EIF4A2	1.12324	0.0011	0.012454
CCDC88A	1.12239	0.0008	0.009825
TIPARP	1.1218	0.0021	0.0204
CCDC50	1.12165	0.001	0.011594
ODC1	1.1214	0.00055	0.007519
RB1CC1	1.12138	0.00085	0.010312
TSG101	1.12094	0.00015	0.002736
TBK1	1.12066	0.00065	0.008431
SETD5-AS1	1.1203	0.001	0.011594
PPP6C	1.12001	0.00245	0.022773
C2orf42	1.11955	0.001	0.011594
SYF2	1.11858	0.0021	0.0204
ARNTL	1.11816	0.00205	0.020098
GPR126	1.1176	0.0008	0.009825
MUC1	1.11741	0.00685	0.048253
NTN4	1.11653	0.0009	0.010731
STK11IP	1.11574	0.00195	0.01938
KDM3A	1.1152	0.00055	0.007519
NR3C1	1.11511	0.00185	0.018595
KPNA4	1.11499	0.0018	0.018195
ZFAT	1.11497	0.0016	0.016584
CUL4B	1.11407	0.00035	0.005299
CPEB4	1.11358	0.00055	0.007519
MAGT1	1.11351	0.00055	0.007519
FNDC3A	1.11221	0.00045	0.006411
ARL14EP	1.11184	0.0012	0.013303
SRP54	1.11177	0.00155	0.016224
PATL1	1.11132	0.00065	0.008431
AKAP8L	1.11123	0.0009	0.010731
SERINC3	1.11052	0.00095	0.011119
TAB2	1.11029	0.0007	0.008891
TLK2	1.11017	0.00045	0.006411
TBPL1	1.11012	0.0018	0.018195
CIR1	1.10922	0.00175	0.017791
HPS5	1.10883	0.0005	0.006979
PPP2CB	1.10871	0.00025	0.004092
STRIP2	1.10864	0.00365	0.030415
ATF7IP2	1.10848	0.0035	0.02963

SENP2	1.10838	0.00065	0.008431
TAF2	1.10819	0.00055	0.007519
BAZ2B	1.10768	0.0003	0.004684
HIST1H2BD	1.10728	0.0003	0.004684
SEPT10	1.10683	0.0023	0.021759
DDX50	1.10643	0.00045	0.006411
PIPSL	1.10643	0.00075	0.009355
GPATCH2	1.10622	0.00055	0.007519
TFE3	1.10621	0.00075	0.009355
RNF138	1.10616	0.0014	0.014968
HIF1A	1.1054	0.00095	0.011119
TOR1AIP1	1.1051	0.0007	0.008891
ITCH	1.10406	0.0006	0.008024
EBF1	1.10394	0.002	0.01979
TCEANC	1.10315	0.0055	0.041143
RASEF	1.10046	0.0008	0.009825
ZNF669	1.09973	0.00115	0.012865
ZFP36L1	1.09809	0.00065	0.008431
TMEM156	1.0979	0.0009	0.010731
HCCS	1.09679	0.0007	0.008891
TNIP1	1.09671	0.0008	0.009825
ZC3H15	1.09483	0.00065	0.008431
PNISR	1.09469	0.00065	0.008431
FRMD4B	1.09448	0.0009	0.010731
SRSF6	1.09368	0.00075	0.009355
PTPN11	1.09303	0.0022	0.021101
CHM	1.0919	0.0027	0.024344
VEZF1	1.09134	0.0009	0.010731
ACOT9	1.09096	0.00065	0.008431
ZC3H12C	1.09033	0.0004	0.005861
NDUFA5	1.09021	0.0071	0.049381
UBA6	1.09008	0.00225	0.021413
KDM5C	1.08906	0.00235	0.022088
ZNF160	1.08792	0.0011	0.012454
DENND5A	1.0862	0.00075	0.009355
SBDSP1	1.08535	0.0027	0.024344
YAF2	1.08533	0.0017	0.017361
YIPF6	1.08512	0.00105	0.012007
ATF7IP	1.08437	0.0005	0.006979
DAAM1	1.08426	0.0022	0.021101
GOLGB1	1.08354	0.0007	0.008891
SAMD8	1.08339	0.00085	0.010312
ANXA3	1.08104	0.0018	0.018195
PPID	1.08058	0.0013	0.014165
FUNDC2	1.07998	0.00155	0.016224
MEPCE	1.07965	0.00325	0.027854

PNRC2	1.07944	0.0006	0.008024
UFC1	1.07944	0.0017	0.017361
LANCL2	1.07652	0.0009	0.010731
WDR47	1.07646	0.00155	0.016224
FLCN	1.07627	0.00265	0.024071
MOK	1.07551	0.00355	0.029785
STAG2	1.07532	0.0009	0.010731
ETV3	1.07458	0.00085	0.010312
DEPDC7	1.07447	0.00505	0.038666
ENO3	1.07402	0.0029	0.025597
WDR45	1.07349	0.00165	0.017002
TERF2IP	1.07274	0.001	0.011594
NOTCH2NL	1.07257	0.00195	0.01938
KDM5A	1.07192	0.00145	0.015348
TMEM47	1.07152	0.0043	0.034308
UBA1	1.07053	0.00165	0.017002
HIST2H2AA3	1.0699	0.00145	0.015348
CCDC90B	1.06897	0.0021	0.0204
RIPK2	1.06832	0.0013	0.014165
BACH1	1.06796	0.0011	0.012454
CDC37L1	1.06676	0.0016	0.016584
NRIP3	1.06663	0.00655	0.046613
LRRC48	1.06583	0.00565	0.041892
ZNF394	1.06554	0.00105	0.012007
ABHD5	1.06506	0.0023	0.021759
RGS20	1.0645	0.00255	0.023375
RBBP6	1.06411	0.0048	0.037289
IRGQ	1.06354	0.0007	0.008891
IRF2	1.06329	0.0022	0.021101
SUV420H1	1.06328	0.0014	0.014968
MTF2	1.06294	0.0016	0.016584
DNAJB6	1.06165	0.00105	0.012007
FAM76B	1.06161	0.0033	0.028192
PSME2	1.06133	0.0012	0.013303
GATA3	1.06031	0.00395	0.032241
PCDH12	1.05995	0.00555	0.041342
RBM23	1.05877	0.00095	0.011119
CDK17	1.05813	0.0015	0.015773
OPTN	1.05809	0.0018	0.018195
CNOT4	1.05765	0.00065	0.008431
PSME4	1.05694	0.00115	0.012865
CENPC1	1.05683	0.0037	0.030688
DPM1	1.05582	0.00175	0.017791
IRAK2	1.0557	0.00085	0.010312
BRCC3	1.05497	0.00185	0.018595
LIPT1	1.05492	0.00495	0.038212

DSP	1.05381	0.00095	0.011119
C12orf61	1.05374	0.0053	0.040041
ADNP2	1.05275	0.00105	0.012007
HNRNPC	1.05255	0.00135	0.014531
ZNF554	1.05234	0.0047	0.036745
HIST1H2BO	1.05175	0.001	0.011594
STX10	1.05037	0.00585	0.042856
PGGT1B	1.04999	0.0023	0.021759
CHCHD7	1.04624	0.0051	0.038881
CLTB	1.04547	0.00475	0.037027
TAF7	1.04499	0.0011	0.012454
ZNF518A	1.04463	0.00235	0.022088
COQ7	1.04398	0.00285	0.025324
ADNP	1.04333	0.00175	0.017791
IKBKE	1.04287	0.00355	0.029785
CTTNBP2NL	1.04256	0.0012	0.013303
RSF1	1.04171	0.00135	0.014531
GCA	1.04169	0.0034	0.028922
VAMP2	1.04085	0.00145	0.015348
PNPLA2	1.04053	0.0025	0.023036
PVRL2	1.0399	0.0027	0.024344
IFNGR2	1.03974	0.00335	0.028542
LRCH3	1.03872	0.00235	0.022088
RRN3P1	1.03675	0.0042	0.033797
MARCH5	1.03643	0.00365	0.030415
CCNYL1	1.03637	0.0013	0.014165
THSD4	1.03585	0.0015	0.015773
SERINC1	1.03479	0.00255	0.023375
ZNF711	1.03449	0.00525	0.039739
CAV1	1.03425	0.00115	0.012865
WBP5	1.03418	0.0062	0.044782
TRIP11	1.03405	0.00085	0.010312
RAPGEF6	1.03352	0.00135	0.014531
TOPORS	1.03345	0.00205	0.020098
AFTPH	1.03332	0.0016	0.016584
ZNF674	1.03212	0.00425	0.034028
ZFP37	1.03199	0.00545	0.040807
CLDN1	1.0319	0.0009	0.010731
RBM48	1.03107	0.0034	0.028922
HNRNPH3	1.03065	0.00485	0.037622
POFUT2	1.02969	0.00365	0.030415
RFFL	1.02968	0.00525	0.039739
TRPC1	1.02964	0.0048	0.037289
SYAP1	1.02914	0.00135	0.014531
ASH1L	1.02862	0.0022	0.021101
RRAS2	1.02815	0.0042	0.033797

FAM210A	1.02813	0.0032	0.027485
STK17B	1.02736	0.0022	0.021101
LOC100130331	1.02716	0.00695	0.048678
OTUD5	1.02681	0.002	0.01979
CREM	1.02662	0.005	0.038449
DDX39B	1.02658	0.0016	0.016584
PBDC1	1.02601	0.0027	0.024344
SPTLC1	1.02565	0.0051	0.038881
NRG1	1.02545	0.0019	0.019025
ARID2	1.02507	0.00145	0.015348
CWF19L2	1.02503	0.00565	0.041892
C3orf52	1.02482	0.00205	0.020098
UBR5	1.02476	0.00135	0.014531
ITSN1	1.02408	0.0029	0.025597
AOC2	1.02368	0.0032	0.027485
B3GNT5	1.02367	0.0049	0.0379
TAB3	1.02172	0.00235	0.022088
ANLN	1.02148	0.0054	0.040509
ESCO1	1.02121	0.00285	0.025324
ZNF350	1.02083	0.0032	0.027485
EIF2AK3	1.01997	0.0014	0.014968
SLC25A14	1.01909	0.0057	0.042028
HIST1H2BM	1.01881	0.0021	0.0204
ZRANB1	1.01832	0.00145	0.015348
USP47	1.01795	0.00115	0.012865
TES	1.01768	0.0017	0.017361
ZBTB5	1.01748	0.00125	0.013761
PSMC6	1.0172	0.0021	0.0204
CAAP1	1.01698	0.0039	0.031948
RBMXL1	1.0161	0.0029	0.025597
MBD1	1.01599	0.00285	0.025324
CSNK1A1	1.01587	0.00115	0.012865
ABCF1	1.01484	0.0013	0.014165
ARL6IP1	1.01484	0.0014	0.014968
PAPOLA	1.01408	0.00195	0.01938
SPAG1	1.01379	0.00305	0.026568
SPRED1	1.01369	0.0021	0.0204
SPPL2A	1.01218	0.0026	0.023711
EGLN1	1.0119	0.00145	0.015348
ZEB1	1.01124	0.00185	0.018595
TRIO	1.01123	0.00275	0.024628
DLGAP5	1.01118	0.0024	0.022413
RAB8B	1.01055	0.00285	0.025324
JHDM1D	1.00699	0.0025	0.023036
SPATA2	1.0064	0.00255	0.023375
RLIM	1.00612	0.00215	0.020747

IER3	1.00567	0.00225	0.021413
CHD4	1.00536	0.00215	0.020747
HIST1H2BE	1.00498	0.0023	0.021759
TCF12	1.00479	0.0025	0.023036
PHTF1	1.00418	0.0035	0.02963
LARP1B	1.0036	0.00635	0.045556
TIFA	1.00221	0.00355	0.029785
EPC2	1.00162	0.0021	0.0204
HIST2H2BE	1.00097	0.0065	0.04634
HIST2H2BF	1.0008	0.0052	0.039473
NAIP	0.999877	0.00365	0.030415
MUC13	0.999642	0.0044	0.034878
AEBP2	0.998282	0.0037	0.030688
POLR2M	0.996881	0.00225	0.021413
ATP13A3	0.994164	0.00255	0.023375
RAB21	0.992341	0.0018	0.018195
UBE2J2	0.991635	0.0048	0.037289
UBALD1	0.991004	0.00705	0.049162
EDN1	0.990304	0.00205	0.020098
PUM1	0.990153	0.0025	0.023036
VCL	0.989227	0.00165	0.017002
MTRF1L	0.98903	0.00235	0.022088
WAC	0.988797	0.002	0.01979
MARCH7	0.986871	0.00175	0.017791
RC3H1	0.986259	0.00195	0.01938
PRPF4B	0.985467	0.0028	0.025006
ABCC9	0.985444	0.00535	0.040324
SRGAP1	0.985043	0.0016	0.016584
SPOP	0.984866	0.0025	0.023036
PCBP2	0.983939	0.00515	0.039205
AKAP10	0.983917	0.0014	0.014968
SAP30BP	0.982956	0.00295	0.025924
CLIC1	0.982553	0.00275	0.024628
GMEB1	0.982541	0.003	0.026176
EAPP	0.982111	0.0031	0.026901
HIST2H2AA4	0.981612	0.0027	0.024344
RHOB	0.981568	0.00265	0.024071
RBM7	0.981293	0.00405	0.032906
GAS5	0.980946	0.00405	0.032906
CSDA	0.980387	0.0039	0.031948
MORF4L2	0.979669	0.0024	0.022413
CA8	0.979447	0.00425	0.034028
RNF11	0.979298	0.0042	0.033797
MFAP1	0.978604	0.0029	0.025597
NDUFAF5	0.978308	0.0048	0.037289
PPP1R15B	0.977785	0.00435	0.03462

WAPAL	0.977236	0.00225	0.021413
USP12	0.976538	0.0038	0.031338
ANKRD12	0.974578	0.0037	0.030688
MON2	0.974177	0.003	0.026176
ECD	0.973267	0.00255	0.023375
ADIPOR2	0.972967	0.00235	0.022088
CC2D1B	0.972353	0.00335	0.028542
KBTBD2	0.971853	0.00205	0.020098
DNAJB12	0.971629	0.0057	0.042028
DYNC1I2	0.971472	0.00295	0.025924
JUNB	0.970553	0.0026	0.023711
PIK3C3	0.970347	0.00605	0.044018
DICER1	0.970202	0.00535	0.040324
MYNN	0.96913	0.00335	0.028542
RAB3GAP1	0.968836	0.00205	0.020098
HNRNPA2B1	0.967965	0.00635	0.045556
SIX4	0.967922	0.0034	0.028922
TMEM62	0.966622	0.005	0.038449
ETF1	0.965757	0.0023	0.021759
SLC41A2	0.964666	0.0048	0.037289
RALGAPA1	0.964378	0.0025	0.023036
GATAD2B	0.96342	0.0027	0.024344
YTHDF3	0.962922	0.0029	0.025597
POGZ	0.961314	0.00425	0.034028
LINC00673	0.959827	0.003	0.026176
FAM13B	0.95975	0.00305	0.026568
C6orf48	0.959475	0.0029	0.025597
SRSF4	0.959136	0.0043	0.034308
SPPL3	0.958579	0.0057	0.042028
NUPL1	0.958449	0.00255	0.023375
OSTM1	0.958134	0.0063	0.04532
LNPEP	0.956611	0.0044	0.034878
CWC25	0.953406	0.00195	0.01938
NKX3-1	0.953289	0.0038	0.031338
NUSAP1	0.952982	0.0031	0.026901
LINC00641	0.952481	0.0054	0.040509
CYCS	0.951169	0.004	0.032616
AGL	0.950046	0.00605	0.044018
BTG2	0.94995	0.0043	0.034308
HNRPDL	0.949317	0.00555	0.041342
STAT3	0.948256	0.0032	0.027485
GPATCH8	0.947647	0.0025	0.023036
PAPD4	0.947612	0.0057	0.042028
ATP6V1B2	0.946381	0.00305	0.026568
MAMLD1	0.944165	0.006	0.043714
RPS6KC1	0.944076	0.0035	0.02963

VDR	0.943429	0.00685	0.048253
BTBD10	0.942926	0.0035	0.02963
CADM1	0.942836	0.0069	0.048541
DOCK4	0.942731	0.0045	0.035424
NAA16	0.941588	0.0063	0.04532
SH3KBP1	0.940829	0.00285	0.025324
NFIL3	0.940595	0.0047	0.036745
THOC2	0.938874	0.0032	0.027485
PPP2CA	0.93882	0.00345	0.0293
SETD2	0.938526	0.0032	0.027485
PKN2	0.937846	0.0046	0.036087
PTPRK	0.936384	0.00425	0.034028
COPS2	0.935769	0.00615	0.044562
SCAND3	0.935564	0.0057	0.042028
COPS5	0.935429	0.00355	0.029785
ATF1	0.934967	0.0048	0.037289
BNIP2	0.934399	0.00655	0.046613
TMEM107	0.934017	0.0044	0.034878
GOLGA4	0.933909	0.0041	0.033278
PTPN14	0.933772	0.0027	0.024344
APLP2	0.930615	0.0042	0.033797
CAPZA1	0.928768	0.0038	0.031338
SENP6	0.92855	0.00685	0.048253
UHRF1BP1L	0.928288	0.00615	0.044562
ATF6	0.927796	0.00355	0.029785
UBXN7	0.927623	0.00495	0.038212
EED	0.926994	0.00455	0.0358
ATAD1	0.925479	0.00445	0.035135
SUPT6H	0.922388	0.0044	0.034878
PPIG	0.921962	0.00535	0.040324
AKIRIN1	0.921176	0.004	0.032616
NCOA4	0.920568	0.00405	0.032906
PELI2	0.919125	0.00695	0.048678
SUZ12	0.918006	0.0053	0.040041
DMTF1	0.917989	0.0047	0.036745
MET	0.917499	0.0053	0.040041
SRSF1	0.917184	0.00715	0.049599
NEMF	0.9169	0.00625	0.045082
SFPQ	0.915485	0.00385	0.031636
SLTM	0.914833	0.0036	0.030077
C12orf4	0.914347	0.00625	0.045082
PPP1R12A	0.914225	0.0043	0.034308
UFM1	0.914221	0.0062	0.044782
TARDBP	0.914055	0.0042	0.033797
BCL2L13	0.913588	0.00345	0.0293
PPM1D	0.912624	0.00485	0.037622

ITSN2	0.91259	0.00285	0.025324
OAZ2	0.911731	0.00585	0.042856
STAG1	0.911643	0.00435	0.03462
STAU1	0.911319	0.00385	0.031636
TBC1D22B	0.910778	0.0065	0.04634
BTBD1	0.910721	0.00415	0.033547
DAGLB	0.910018	0.0063	0.04532
CREB1	0.907635	0.0051	0.038881
TCF7L2	0.905408	0.00375	0.031038
UBXN1	0.904446	0.00555	0.041342
IWS1	0.903814	0.00445	0.035135
ALKBH1	0.900117	0.0045	0.035424
SMCHD1	0.900114	0.00555	0.041342
ZNF410	0.899236	0.0039	0.031948
PARVA	0.898659	0.0067	0.047427
FBXO28	0.897479	0.00435	0.03462
TUG1	0.895126	0.0067	0.047427
MAP3K7	0.893316	0.00575	0.04224
RC3H2	0.891882	0.0051	0.038881
CEP350	0.890641	0.00545	0.040807
EMP3	0.889585	0.00695	0.048678
TOR1B	0.88634	0.0059	0.043143
TNKS2	0.884996	0.0049	0.0379
CTR9	0.884741	0.0054	0.040509
IP6K2	0.884159	0.0071	0.049381
KIF5B	0.883184	0.00615	0.044562
IST1	0.882733	0.0057	0.042028
WEE1	0.882408	0.005	0.038449
EAF1	0.88239	0.00535	0.040324
TNKS	0.881736	0.00445	0.035135
FJX1	0.881472	0.0072	0.049859
RIPK1	0.880979	0.00585	0.042856
MED1	0.880848	0.0066	0.046885
PNP	0.880414	0.006	0.043714
SVIL	0.879037	0.0054	0.040509
PTPN21	0.877526	0.0058	0.042568
KIAA0907	0.875833	0.0057	0.042028
PAPOLG	0.874308	0.00605	0.044018
MTMR9	0.87228	0.00575	0.04224
MCU	0.870852	0.0061	0.044281
ECT2	0.87073	0.0064	0.045812
CHD1	0.869651	0.00665	0.047198
NHS	0.866605	0.0065	0.04634
TBX3	0.86516	0.0061	0.044281
RBFOX2	0.86253	0.0059	0.043143
HIST1H4B	0.858592	0.00695	0.048678

FRYL	0.855451	0.00715	0.049599
PSEN1	0.855309	0.0072	0.049859
KIDINS220	0.853756	0.00675	0.047759
RBBP5	0.851055	0.00695	0.048678
SRSF7	0.844134	0.0063	0.04532
PTK2	0.84144	0.00705	0.049162
BPTF	0.838359	0.0072	0.049859
CDCA5	-0.85935	0.0071	0.049381
KLF11	-0.86587	0.00715	0.049599
GPRIN3	-0.86737	0.0061	0.044281
NFE2L1	-0.87342	0.0067	0.047427
FADS1	-0.8736	0.0068	0.048028
GCNT3	-0.8736	0.0046	0.036087
CDCA4	-0.88273	0.0057	0.042028
MID1IP1	-0.88344	0.00475	0.037027
WWC3	-0.88549	0.005	0.038449
SCD	-0.8859	0.0057	0.042028
TRIM9	-0.88653	0.00645	0.046066
SRPRB	-0.88879	0.0069	0.048541
MCM10	-0.89194	0.00505	0.038666
PACSIN2	-0.89236	0.0065	0.04634
ARRDC2	-0.89246	0.0066	0.046885
NUDT19	-0.89301	0.0049	0.0379
LMNB2	-0.89656	0.00645	0.046066
FAM136A	-0.90148	0.00555	0.041342
ZNF749	-0.90167	0.00645	0.046066
GPI	-0.90287	0.0071	0.049381
TOR3A	-0.90384	0.00595	0.043449
SLC45A4	-0.9043	0.0064	0.045812
ARRB2	-0.90937	0.00695	0.048678
MTSS1L	-0.90979	0.0068	0.048028
LARP1	-0.91376	0.00395	0.032241
FOXE1	-0.9166	0.00695	0.048678
LRRC47	-0.91668	0.0062	0.044782
SLC4A2	-0.9175	0.0062	0.044782
VANGL1	-0.9196	0.0051	0.038881
KBTBD6	-0.91962	0.00505	0.038666
IMP3	-0.91984	0.00425	0.034028
IQGAP3	-0.92095	0.00575	0.04224
RIN2	-0.92142	0.0037	0.030688
KIAA0930	-0.92152	0.0046	0.036087
GAREML	-0.92526	0.0064	0.045812
RRS1	-0.9255	0.006	0.043714
MCM3	-0.92706	0.0036	0.030077
RPIA	-0.93276	0.00615	0.044562
ZNF607	-0.93443	0.00635	0.045556

C11orf95	-0.9367	0.00505	0.038666
EXD2	-0.9398	0.0052	0.039473
MCM6	-0.94	0.0027	0.024344
RSAD1	-0.94052	0.0032	0.027485
MRPS26	-0.94136	0.0049	0.0379
RHOBTB1	-0.94366	0.0042	0.033797
ATP5G1	-0.94394	0.00555	0.041342
TRMT61A	-0.94562	0.0044	0.034878
ACSS1	-0.94649	0.0063	0.04532
REEP4	-0.947	0.0058	0.042568
ZNF768	-0.94834	0.00405	0.032906
C1orf216	-0.94998	0.005	0.038449
FADD	-0.9502	0.0025	0.023036
SLC52A2	-0.95534	0.0054	0.040509
BCL7A	-0.95589	0.0035	0.02963
NIPSNAP1	-0.95743	0.00665	0.047198
IGSF11	-0.95784	0.0063	0.04532
KBTBD11	-0.96038	0.00335	0.028542
CNNM1	-0.96086	0.0028	0.025006
MANEAL	-0.96116	0.0069	0.048541
LBH	-0.96255	0.00405	0.032906
MRI1	-0.96392	0.0056	0.041637
CCNF	-0.964	0.00355	0.029785
MGST1	-0.9671	0.0046	0.036087
SPATA20	-0.96732	0.00525	0.039739
FAM213A	-0.9702	0.0056	0.041637
CA12	-0.97038	0.00705	0.049162
CYB561	-0.97129	0.00295	0.025924
APRT	-0.9723	0.0054	0.040509
AP1S1	-0.97339	0.0047	0.036745
PSAT1	-0.97417	0.0037	0.030688
ZNF30	-0.97435	0.00685	0.048253
EDEM2	-0.97744	0.00575	0.04224
WDR5	-0.97934	0.0032	0.027485
NEIL3	-0.97945	0.0036	0.030077
FBXW10	-0.97962	0.00445	0.035135
GAB2	-0.98183	0.0025	0.023036
TCF19	-0.98251	0.0067	0.047427
UBE2T	-0.98265	0.00345	0.0293
RAB26	-0.98309	0.0028	0.025006
ZNF343	-0.98482	0.00215	0.020747
TRAPPC1	-0.98753	0.00435	0.03462
PTCD2	-0.98785	0.00625	0.045082
TMTC4	-0.98804	0.0054	0.040509
LRP8	-0.9883	0.0038	0.031338
AES	-0.98868	0.00555	0.041342

KCTD1	-0.98918	0.0026	0.023711
ZC3HAV1L	-0.98955	0.00405	0.032906
C17orf70	-0.99086	0.0038	0.031338
FECH	-0.99098	0.00255	0.023375
AMPD2	-0.99153	0.0045	0.035424
FAM129B	-0.99241	0.00255	0.023375
MAMDC4	-0.99503	0.0057	0.042028
PFN2	-0.99508	0.00355	0.029785
GIT1	-0.99615	0.0037	0.030688
BCAT1	-0.99805	0.00475	0.037027
OLFML2A	-0.99879	0.00475	0.037027
ZNF526	-0.99898	0.00315	0.027261
BLVRA	-1.00095	0.00545	0.040807
ZFP64	-1.00102	0.00225	0.021413
VOPP1	-1.00181	0.00245	0.022773
FIGNL1	-1.00184	0.00265	0.024071
CAD	-1.00202	0.0022	0.021101
MOCOS	-1.00304	0.0042	0.033797
NFIA	-1.00324	0.00185	0.018595
POP1	-1.0043	0.00215	0.020747
MRPL12	-1.00531	0.00275	0.024628
CCM2	-1.00736	0.007	0.04892
TNFRSF1A	-1.00776	0.0032	0.027485
PIK3C2B	-1.00783	0.0021	0.0204
ADD2	-1.00821	0.0016	0.016584
RECQL4	-1.0083	0.00355	0.029785
POLR3K	-1.00837	0.0055	0.041143
MRM1	-1.00927	0.00285	0.025324
NAGA	-1.01107	0.00715	0.049599
MALL	-1.01336	0.00355	0.029785
CRTAP	-1.01585	0.00145	0.015348
CNTNAP1	-1.01696	0.00505	0.038666
FICD	-1.01711	0.003	0.026176
SLC35C1	-1.01719	0.00245	0.022773
SLC29A3	-1.0173	0.00505	0.038666
SNX18	-1.01856	0.0049	0.0379
STRA13	-1.0194	0.00465	0.036443
E2F8	-1.01962	0.00295	0.025924
NABP2	-1.02046	0.0023	0.021759
GID4	-1.02106	0.00165	0.017002
ALDH3B1	-1.02121	0.0027	0.024344
RNASEH2A	-1.02185	0.00535	0.040324
KBTBD7	-1.02374	0.0024	0.022413
SRM	-1.02387	0.0045	0.035424
HEATR3	-1.02447	0.00215	0.020747
SLC27A4	-1.02449	0.00245	0.022773

FOXF2	-1.02525	0.0051	0.038881
ERP29	-1.0253	0.0016	0.016584
PCLO	-1.02538	0.0067	0.047427
LSM10	-1.02583	0.007	0.04892
CAT	-1.02701	0.0028	0.025006
FCHO1	-1.02711	0.00715	0.049599
NQO1	-1.0301	0.003	0.026176
ANKRD13D	-1.03033	0.00315	0.027261
POLRMT	-1.03255	0.0038	0.031338
ZNF598	-1.03388	0.00215	0.020747
TRIM35	-1.03445	0.0016	0.016584
NRSN2	-1.0348	0.0033	0.028192
APOBEC3C	-1.03669	0.0051	0.038881
RPS6KA4	-1.03844	0.0032	0.027485
CYFIP2	-1.04086	0.0039	0.031948
SCARA5	-1.04226	0.00575	0.04224
ST6GALNAC2	-1.04243	0.00645	0.046066
CYB5RL	-1.04281	0.0032	0.027485
MPI	-1.04316	0.00245	0.022773
ZNF786	-1.04402	0.0019	0.019025
HHIPL2	-1.04497	0.005	0.038449
PDXP	-1.0458	0.0021	0.0204
TP53I13	-1.04581	0.0043	0.034308
GTF2IRD1	-1.0466	0.0032	0.027485
STOX2	-1.04803	0.0018	0.018195
DCTPP1	-1.04866	0.002	0.01979
PGM1	-1.05102	0.00425	0.034028
PTGR1	-1.05131	0.0032	0.027485
FEN1	-1.05221	0.0012	0.013303
TSEN54	-1.0525	0.00415	0.033547
NUP210	-1.05402	0.0017	0.017361
TKT	-1.05403	0.0059	0.043143
RNF187	-1.05449	0.0016	0.016584
VKORC1	-1.05517	0.00475	0.037027
C9orf3	-1.05677	0.0006	0.008024
IGSF3	-1.05701	0.0012	0.013303
AHNAK2	-1.05734	0.0044	0.034878
ABCA3	-1.05801	0.0022	0.021101
NUMBL	-1.05951	0.00105	0.012007
TMED3	-1.06005	0.00175	0.017791
FUT1	-1.06013	0.00715	0.049599
RTKN	-1.06193	0.00355	0.029785
HDHD3	-1.06231	0.00145	0.015348
RBFA	-1.06236	0.00135	0.014531
EBPL	-1.06249	0.00575	0.04224
C19orf48	-1.06271	0.00165	0.017002

CCDC51	-1.06275	0.00225	0.021413
FNDC8	-1.06275	0.00265	0.024071
EVPL	-1.06279	0.00135	0.014531
CHRNG	-1.06398	0.00465	0.036443
UCK2	-1.06405	0.0008	0.009825
MED22	-1.06417	0.0011	0.012454
ALDH1L2	-1.06458	0.00225	0.021413
MCM2	-1.06467	0.0011	0.012454
PDCD2L	-1.0647	0.00155	0.016224
EPS8L2	-1.06649	0.0024	0.022413
DGKG	-1.06688	0.00145	0.015348
PPP1R3E	-1.06777	0.0024	0.022413
ANK2	-1.06898	0.0052	0.039473
EPDR1	-1.07043	0.00055	0.007519
C1orf74	-1.07156	0.0026	0.023711
PREB	-1.07414	0.00185	0.018595
HTATSF1P2	-1.07506	0.00085	0.010312
B3GAT3	-1.07514	0.00325	0.027854
SDC3	-1.07634	0.00445	0.035135
PELO	-1.07689	0.00065	0.008431
ADORA2B	-1.07708	0.00195	0.01938
ST3GAL4	-1.07876	0.005	0.038449
E2F1	-1.07878	0.00175	0.017791
ABHD14B	-1.08014	0.00275	0.024628
PTCH1	-1.08179	0.00205	0.020098
HAGH	-1.08207	0.0062	0.044782
TUBAL3	-1.08219	0.0039	0.031948
PSMG3	-1.08222	0.0027	0.024344
NGFRAP1	-1.08304	0.0025	0.023036
AGAP3	-1.08344	0.0023	0.021759
TBC1D2B	-1.08558	0.00115	0.012865
AP3M2	-1.08854	0.00095	0.011119
FAM217B	-1.08886	0.0068	0.048028
XYLB	-1.08891	0.00275	0.024628
OXLD1	-1.09008	0.00395	0.032241
AMER1	-1.0904	0.0008	0.009825
DBN1	-1.0905	0.0031	0.026901
FGFR4	-1.09148	0.0029	0.025597
GPD1L	-1.09212	0.0017	0.017361
CHST3	-1.09294	0.00065	0.008431
TUB	-1.0931	0.002	0.01979
FADS2	-1.09389	0.00125	0.013761
PCBD1	-1.09517	0.00075	0.009355
NLE1	-1.09531	0.0007	0.008891
C20orf112	-1.09675	0.00065	0.008431
LEPREL4	-1.09787	0.0009	0.010731

DAG1	-1.09909	0.0007	0.008891
RASSF6	-1.09945	0.00415	0.033547
SOGA1	-1.10071	0.0016	0.016584
TUBA1B	-1.10177	0.00105	0.012007
FOXD2	-1.10205	0.00705	0.049162
GGT1	-1.10522	0.0024	0.022413
PRDX1	-1.10766	0.0008	0.009825
GSR	-1.10873	0.0005	0.006979
RTN4RL2	-1.11276	0.00095	0.011119
PNMAL1	-1.11327	0.00205	0.020098
FAM174B	-1.11409	0.00295	0.025924
POC1A	-1.11435	0.00355	0.029785
HECTD3	-1.11456	0.00165	0.017002
TRIM16	-1.11707	0.001	0.011594
RALGDS	-1.11778	0.00215	0.020747
WIPF1	-1.11861	0.00115	0.012865
GINS2	-1.11899	0.0015	0.015773
TRIM65	-1.11917	0.0005	0.006979
PTPN18	-1.12128	0.00155	0.016224
PRRT3	-1.12159	0.0029	0.025597
ANKRD39	-1.1218	0.00355	0.029785
ORAI3	-1.12349	0.0036	0.030077
PECR	-1.12388	0.0013	0.014165
SLC25A10	-1.12462	0.00325	0.027854
DTX4	-1.1258	0.00105	0.012007
PSRC1	-1.12633	0.0017	0.017361
STBD1	-1.12717	0.00395	0.032241
IDH1	-1.12758	0.0004	0.005861
C22orf29	-1.12801	0.0007	0.008891
BLOC1S4	-1.12812	0.0021	0.0204
TMEM106C	-1.13022	0.00095	0.011119
TALDO1	-1.13038	0.0005	0.006979
PDXK	-1.13155	0.00105	0.012007
LOC100506054	-1.13207	0.00215	0.020747
VLDLR	-1.13368	0.00125	0.013761
B3GALT6	-1.13385	0.0017	0.017361
FOXA2	-1.13903	0.0005	0.006979
MCM5	-1.13978	0.00055	0.007519
G6PD	-1.14009	0.0026	0.023711
ZNF239	-1.14037	0.0037	0.030688
DFNA5	-1.14065	0.0021	0.0204
XK	-1.14092	0.007	0.04892
NQO2	-1.14274	0.0006	0.008024
TECR	-1.14542	0.00065	0.008431
VPS37D	-1.14688	0.0052	0.039473
GLTPD1	-1.1472	0.00205	0.020098

SCARB1	-1.14751	0.00065	0.008431
VAV3	-1.14758	0.00105	0.012007
CDRT1	-1.14812	0.0007	0.008891
LINC00842	-1.14919	0.00355	0.029785
LZTS2	-1.15107	0.0024	0.022413
KIF26A	-1.15173	0.007	0.04892
TMEM141	-1.15222	0.0037	0.030688
ISOC2	-1.1525	0.0028	0.025006
MSTO1	-1.15335	0.00115	0.012865
LRRC23	-1.15407	0.00415	0.033547
KCTD17	-1.15511	0.00525	0.039739
CDC25A	-1.15568	0.0005	0.006979
PACSIN3	-1.15581	0.00225	0.021413
CBX6	-1.15592	0.0003	0.004684
TP53I11	-1.15653	0.00205	0.020098
GEMIN4	-1.15742	0.0003	0.004684
USP51	-1.15933	0.00365	0.030415
BRI3BP	-1.15997	0.0012	0.013303
FBXL16	-1.16087	0.00385	0.031636
AKAP1	-1.16163	0.0003	0.004684
PKN1	-1.16186	0.00075	0.009355
PBXIP1	-1.16194	0.00185	0.018595
ZDHHC8	-1.16259	0.00505	0.038666
KCNK5	-1.16275	0.00105	0.012007
RGS3	-1.16351	0.001	0.011594
ABCC2	-1.16386	0.0014	0.014968
SLC19A1	-1.1641	0.003	0.026176
RABAC1	-1.16486	0.00355	0.029785
PPAP2C	-1.16575	0.00195	0.01938
CROT	-1.16719	0.0047	0.036745
SEL1L3	-1.16774	0.0005	0.006979
MCIN	-1.16798	0.0045	0.035424
FANCF	-1.16834	0.0009	0.010731
PHLDA3	-1.1693	0.00095	0.011119
HR	-1.17015	0.001	0.011594
MEST	-1.17029	0.0064	0.045812
ANK3	-1.1732	0.0044	0.034878
CTDSPL	-1.17332	0.00175	0.017791
SAPCD2	-1.17349	0.0008	0.009825
KIAA1161	-1.17782	0.0011	0.012454
CTSF	-1.17825	0.0054	0.040509
EXOSC5	-1.1793	0.0013	0.014165
GLT25D1	-1.18038	0.0002	0.003428
FAM20C	-1.1806	0.0009	0.010731
HGD	-1.18141	0.0009	0.010731
DDIT4L	-1.18161	0.00425	0.034028

SLC1A4	-1.18215	0.0004	0.005861
LEPREL2	-1.1837	0.0025	0.023036
INF2	-1.18385	0.00265	0.024071
CLPP	-1.18395	0.00375	0.031038
SLC5A11	-1.18476	0.00115	0.012865
ZNF362	-1.18534	0.0012	0.013303
CERCAM	-1.18605	0.00205	0.020098
PLD6	-1.18662	0.00025	0.004092
C11orf45	-1.18709	0.00415	0.033547
ZNF287	-1.18982	0.0011	0.012454
CENPB	-1.18987	0.00045	0.006411
SIGMAR1	-1.19086	0.00025	0.004092
SKIDA1	-1.19335	0.00125	0.013761
KEAP1	-1.19344	0.00025	0.004092
KANK2	-1.1937	0.0003	0.004684
ZBTB14	-1.19374	0.0009	0.010731
CSRP2BP	-1.19632	0.0031	0.026901
VPS26B	-1.19639	0.00095	0.011119
UNKL	-1.19706	0.0004	0.005861
ZBTB12	-1.20039	0.00405	0.032906
C15orf39	-1.20073	0.00065	0.008431
GPR135	-1.2009	0.00115	0.012865
GAMT	-1.20101	0.00655	0.046613
TMEM177	-1.20353	0.00055	0.007519
KIAA0319	-1.20446	0.00025	0.004092
DSCAML1	-1.20458	0.0033	0.028192
KCNG1	-1.2062	0.00285	0.025324
FAM86EP	-1.20776	0.0006	0.008024
IGSF8	-1.20966	0.00505	0.038666
FBXW5	-1.20989	0.00055	0.007519
GPT2	-1.21377	0.0002	0.003428
THNSL1	-1.21736	0.00425	0.034028
GAL3ST1	-1.21773	0.00285	0.025324
RGS19	-1.21849	0.00225	0.021413
CDC42EP1	-1.21999	0.0004	0.005861
PGP	-1.2211	0.0003	0.004684
CYP4F11	-1.22257	5.00E-05	0.001089
HNF1A	-1.22288	0.00015	0.002736
MYLK	-1.22373	0.00235	0.022088
AGR2	-1.22487	0.0005	0.006979
AKR1B10	-1.22576	0.0026	0.023711
ZNF395	-1.2272	0.00035	0.005299
NAAA	-1.22737	0.0015	0.015773
TRIM16L	-1.22744	0.0004	0.005861
FAM102A	-1.228	0.00035	0.005299
SLC45A1	-1.2288	0.0032	0.027485

DHRS13	-1.22925	0.0025	0.023036
DHCR24	-1.22952	0.00015	0.002736
SLC16A13	-1.23029	0.00105	0.012007
KIF21B	-1.23059	0.00045	0.006411
DISP2	-1.23359	0.0062	0.044782
GATA2	-1.23427	0.00235	0.022088
AKR1A1	-1.23558	0.00045	0.006411
EPHB4	-1.23628	0.0004	0.005861
TRIM17	-1.23777	0.0013	0.014165
SCARA3	-1.23958	0.0007	0.008891
KLHL31	-1.23988	0.00395	0.032241
HRAS	-1.2403	0.0015	0.015773
MXD4	-1.24167	0.001	0.011594
PAQR9	-1.24217	0.0007	0.008891
FLYWCH1	-1.24277	0.00065	0.008431
PCSK9	-1.24279	0.0022	0.021101
C1orf106	-1.24347	0.0004	0.005861
PNMA2	-1.24368	0.0001	0.001954
FXYD2	-1.2445	0.00115	0.012865
LOC388906	-1.24468	0.00435	0.03462
LOC643401	-1.24565	0.00095	0.011119
SIRPA	-1.24588	0.00025	0.004092
FAM155B	-1.24887	0.0005	0.006979
ASB13	-1.24912	0.00045	0.006411
CELSR2	-1.24962	0.00095	0.011119
DLX4	-1.24992	0.00315	0.027261
C8orf47	-1.25062	0.0036	0.030077
SORBS3	-1.25085	0.0005	0.006979
BLVRB	-1.25146	0.0001	0.001954
RNFT2	-1.25286	0.0003	0.004684
NDUFB10	-1.25306	0.0009	0.010731
MAZ	-1.25456	0.00015	0.002736
MANSC1	-1.25568	0.00085	0.010312
TMEM9	-1.25592	0.0003	0.004684
ZNF219	-1.25684	0.0044	0.034878
C20orf27	-1.2576	0.0004	0.005861
CKAP4	-1.25937	0.00025	0.004092
KLF13	-1.25947	0.0001	0.001954
MLLT11	-1.26182	0.0002	0.003428
SLC46A1	-1.26192	0.0005	0.006979
DNASE2	-1.26262	0.00025	0.004092
TMC4	-1.26379	0.00315	0.027261
KRT86	-1.26572	0.00135	0.014531
LOC348761	-1.26841	0.0061	0.044281
RAB3D	-1.27063	0.0003	0.004684
ZBTB7B	-1.27071	0.0001	0.001954

DNLZ	-1.27076	0.0067	0.047427
CBX8	-1.27132	0.00035	0.005299
SMAD6	-1.27232	0.0003	0.004684
SPP1	-1.27458	5.00E-05	0.001089
ZNF48	-1.27565	0.0005	0.006979
HNF4A	-1.27612	0.0015	0.015773
C9orf69	-1.28022	0.00025	0.004092
TUBB	-1.28443	0.00025	0.004092
SPC24	-1.28595	0.0024	0.022413
CDCA7	-1.28738	0.00015	0.002736
MNX1	-1.2881	0.0019	0.019025
CBS	-1.2885	0.00035	0.005299
GSTM4	-1.29114	0.00495	0.038212
TMEM161A	-1.2917	0.00235	0.022088
CEBPA	-1.29229	0.00015	0.002736
FAM57A	-1.2925	0.00015	0.002736
ZNF469	-1.2928	5.00E-05	0.001089
PPP1R26	-1.29435	5.00E-05	0.001089
NCS1	-1.29548	0.00015	0.002736
C12orf68	-1.29573	0.0057	0.042028
LPCAT4	-1.29639	0.0002	0.003428
HTR1D	-1.29919	0.00025	0.004092
SLC13A4	-1.29925	0.0056	0.041637
LOC257396	-1.30034	0.0005	0.006979
CAMKK1	-1.30114	0.0003	0.004684
METRN	-1.30216	0.00245	0.022773
TTLL6	-1.30299	0.00235	0.022088
C16orf59	-1.30376	0.0006	0.008024
CDCA3	-1.30385	0.0001	0.001954
HPS6	-1.30435	0.00015	0.002736
ZNF792	-1.30529	0.0002	0.003428
AGPAT2	-1.30859	5.00E-05	0.001089
TRAPPC5	-1.30947	0.0009	0.010731
SMO	-1.30985	0.0003	0.004684
ABHD15	-1.31239	5.00E-05	0.001089
TIGD5	-1.31239	0.00025	0.004092
C3orf72	-1.31275	0.0011	0.012454
GTDC2	-1.31408	0.0006	0.008024
MRPL24	-1.31541	0.00015	0.002736
SLC29A1	-1.31612	0.00025	0.004092
C1QTNF6	-1.31892	0.00105	0.012007
UNG	-1.32349	0.00035	0.005299
HILPDA	-1.32683	0.0001	0.001954
CORO2A	-1.32731	0.0002	0.003428
CDT1	-1.32738	0.00015	0.002736
HNF1B	-1.32817	5.00E-05	0.001089

PODXL2	-1.32876	0.0017	0.017361
GPC1	-1.32935	0.00015	0.002736
CPLX2	-1.33059	0.0003	0.004684
SAC3D1	-1.3308	0.00445	0.035135
CDR2L	-1.33099	0.0001	0.001954
C1orf220	-1.3322	0.00065	0.008431
SMKR1	-1.33222	0.00695	0.048678
HJURP	-1.33316	0.00015	0.002736
ID1	-1.33326	0.0002	0.003428
PGD	-1.33486	0.0002	0.003428
RAP1GAP	-1.33572	0.0002	0.003428
NR2F6	-1.33602	0.0001	0.001954
CAPN5	-1.33662	0.0003	0.004684
SLC48A1	-1.33713	0.0031	0.026901
PHGDH	-1.33789	0.00015	0.002736
GALNT12	-1.33847	0.0051	0.038881
SNX33	-1.33952	0.0002	0.003428
SLC47A2	-1.34236	0.00145	0.015348
MFSD3	-1.34346	0.00065	0.008431
NEURL1B	-1.34352	0.0014	0.014968
CCNJL	-1.34364	0.00015	0.002736
GNB3	-1.34429	0.00135	0.014531
BCRP2	-1.34544	0.00545	0.040807
MAMSTR	-1.34802	0.00395	0.032241
C15orf55	-1.34865	0.00415	0.033547
NAT8L	-1.34956	0.0003	0.004684
SLC9A3R2	-1.35009	0.00095	0.011119
VIL1	-1.35083	0.00695	0.048678
EPHX1	-1.35332	0.00025	0.004092
FGFR3	-1.35351	0.00105	0.012007
PIR	-1.35762	0.00115	0.012865
TRIM7	-1.35817	0.00125	0.013761
TMPRSS6	-1.35854	0.00595	0.043449
SKP2	-1.35859	0.0012	0.013303
B4GALNT1	-1.35879	0.0002	0.003428
RAB20	-1.35909	0.0024	0.022413
CARNS1	-1.3605	0.0062	0.044782
JDP2	-1.36202	5.00E-05	0.001089
CEND1	-1.36485	0.0043	0.034308
TUSC1	-1.36519	0.00105	0.012007
MSC	-1.36685	0.00025	0.004092
SLC27A2	-1.36815	0.00035	0.005299
LRP3	-1.36896	0.0002	0.003428
C5orf4	-1.37208	0.00035	0.005299
DHODH	-1.37348	5.00E-05	0.001089
ZFP69B	-1.37371	0.00115	0.012865

LRRC16B	-1.37411	0.00215	0.020747
ANKRD2	-1.37482	0.0036	0.030077
FAM64A	-1.37769	0.0002	0.003428
MARCH1	-1.37986	0.0008	0.009825
HSD17B1	-1.38369	0.00015	0.002736
DGCR5	-1.38908	0.00095	0.011119
AMBP	-1.39141	0.0017	0.017361
MIRLET7BHG	-1.39339	5.00E-05	0.001089
KCTD15	-1.39898	5.00E-05	0.001089
EGFL7	-1.39901	0.00115	0.012865
MMP24	-1.39999	0.0001	0.001954
LOC284798	-1.40208	0.00655	0.046613
GATS	-1.40209	0.0006	0.008024
CENPM	-1.40288	0.0008	0.009825
CBX7	-1.4048	0.0006	0.008024
LOC100129726	-1.40767	0.00025	0.004092
EPHX2	-1.40781	0.00045	0.006411
HYAL1	-1.40799	0.0011	0.012454
PRODH2	-1.40807	0.0066	0.046885
C2orf72	-1.40973	5.00E-05	0.001089
NME4	-1.41007	5.00E-05	0.001089
LIPT2	-1.41365	0.00225	0.021413
GSTP1	-1.41547	5.00E-05	0.001089
MRPS34	-1.41568	5.00E-05	0.001089
DIO3OS	-1.41573	0.00575	0.04224
ABHD4	-1.4192	5.00E-05	0.001089
C10orf35	-1.4225	0.00015	0.002736
SMARCD3	-1.42438	0.00025	0.004092
ARHGEF37	-1.4255	0.0003	0.004684
GDAP1L1	-1.42704	0.00295	0.025924
BRWD1-IT2	-1.42721	0.00425	0.034028
HENMT1	-1.42879	0.00165	0.017002
ALDH1A1	-1.42974	0.00025	0.004092
APOH	-1.4311	0.0029	0.025597
SLCO2B1	-1.43256	0.00075	0.009355
F12	-1.43743	0.00075	0.009355
CDA	-1.44026	0.00245	0.022773
RIMS3	-1.44215	0.0001	0.001954
CDPF1	-1.44296	0.00065	0.008431
TMEM102	-1.44523	0.0004	0.005861
CBR1	-1.44554	5.00E-05	0.001089
LENEP	-1.44871	0.0023	0.021759
SDC1	-1.44991	5.00E-05	0.001089
KLHL35	-1.45052	0.00205	0.020098
SLC2A4RG	-1.45062	0.00445	0.035135
TMEM246	-1.4523	5.00E-05	0.001089

NDUFB7	-1.45425	0.00035	0.005299
FGG	-1.45455	0.0008	0.009825
RPP25	-1.45653	5.00E-05	0.001089
NME3	-1.45882	0.00055	0.007519
AKR1C4	-1.45945	0.0001	0.001954
AKR1B15	-1.46134	5.00E-05	0.001089
HOXB13	-1.46148	0.0003	0.004684
EPN3	-1.46193	0.00035	0.005299
DCLRE1B	-1.46354	5.00E-05	0.001089
IDH2	-1.46374	0.0003	0.004684
CDH1	-1.46665	5.00E-05	0.001089
ZBTB47	-1.46685	5.00E-05	0.001089
ALDH1B1	-1.46793	5.00E-05	0.001089
GAS2L1	-1.46856	0.0004	0.005861
DGCR6	-1.46879	0.00255	0.023375
FAM226B	-1.47295	0.0021	0.0204
SIRPB1	-1.47446	0.00195	0.01938
F8A1,F8A3	-1.4746	0.00385	0.031636
S100P	-1.47635	5.00E-05	0.001089
TEX40	-1.47692	0.0046	0.036087
FAM86C2P	-1.47866	0.0001	0.001954
P2RY6	-1.47923	5.00E-05	0.001089
WNT10B	-1.47961	0.00125	0.013761
ZNF488	-1.48612	5.00E-05	0.001089
TMEM139	-1.48713	0.0019	0.019025
HINT2	-1.48795	0.0001	0.001954
RAB40B	-1.48836	0.00065	0.008431
TSKU	-1.48958	0.0002	0.003428
NR0B1	-1.49427	5.00E-05	0.001089
ADCK2	-1.49579	0.0001	0.001954
AKNA	-1.49694	5.00E-05	0.001089
PLEKHH3	-1.49748	5.00E-05	0.001089
GJD3	-1.50215	5.00E-05	0.001089
ACOT11	-1.50402	0.00635	0.045556
SYBU	-1.50416	5.00E-05	0.001089
TSPO	-1.50604	0.0006	0.008024
LRRC20	-1.50653	5.00E-05	0.001089
TTLL12	-1.50932	5.00E-05	0.001089
LHPP	-1.51042	0.00065	0.008431
S1PR5	-1.51097	0.0005	0.006979
SOX12	-1.51332	5.00E-05	0.001089
UFSP1	-1.51358	0.0004	0.005861
EIF4EBP1	-1.51546	5.00E-05	0.001089
SNTB1	-1.51892	5.00E-05	0.001089
THEM6	-1.51967	5.00E-05	0.001089
PIM1	-1.51999	5.00E-05	0.001089

FLVCR1-AS1	-1.52177	0.00085	0.010312
MXRA8	-1.52256	0.0025	0.023036
PLEKHF1	-1.5233	0.00015	0.002736
NTN3	-1.52543	0.00185	0.018595
DEPTOR	-1.52823	0.0023	0.021759
PRRG2	-1.53379	0.00685	0.048253
LOC100505761	-1.53766	0.0008	0.009825
SYTL1	-1.53995	0.0064	0.045812
MARVELD1	-1.54128	5.00E-05	0.001089
LOC440518	-1.54776	0.00035	0.005299
P2RX2	-1.54788	0.0017	0.017361
NS3BP	-1.54849	5.00E-05	0.001089
TP53TG1	-1.54905	0.0003	0.004684
GATSL2	-1.55145	0.0035	0.02963
GALK1	-1.55373	0.00015	0.002736
SLC16A14	-1.55552	0.0006	0.008024
ZNF232	-1.55612	0.00015	0.002736
F2RL2	-1.55634	0.0002	0.003428
SLC40A1	-1.55639	5.00E-05	0.001089
SLC29A4	-1.55681	0.0005	0.006979
RGS14	-1.56101	0.00065	0.008431
MAFG-AS1	-1.56356	5.00E-05	0.001089
AKR1C3	-1.56418	5.00E-05	0.001089
UBL4A	-1.5658	5.00E-05	0.001089
CFD	-1.56838	0.00145	0.015348
MATN2	-1.56886	0.0002	0.003428
GPR20	-1.57126	0.0007	0.008891
IGSF23	-1.57171	0.00275	0.024628
TMEM105	-1.57214	0.00095	0.011119
RPUSD1	-1.57219	5.00E-05	0.001089
ZNF688	-1.57351	0.00055	0.007519
CYP4F12	-1.57483	0.00105	0.012007
GPT	-1.57537	0.0005	0.006979
LINC00235	-1.57836	0.00195	0.01938
SULT2B1	-1.57876	0.00015	0.002736
ASIC5	-1.58058	0.00135	0.014531
PARD6A	-1.58639	0.0007	0.008891
PWWP2B	-1.58847	5.00E-05	0.001089
HSPA12B	-1.58951	5.00E-05	0.001089
PRODH	-1.59292	0.0026	0.023711
LOC151009	-1.59562	0.00295	0.025924
GPR162	-1.59677	0.00015	0.002736
SDR42E1	-1.59732	0.00095	0.011119
MPST	-1.59789	0.003	0.026176
PCYOX1L	-1.60115	0.0001	0.001954
ACSBG2	-1.60656	0.00515	0.039205

RERG	-1.60829	0.00135	0.014531
ZNF467	-1.62272	0.0045	0.035424
CRIP2	-1.62462	0.0015	0.015773
SLC7A5	-1.62569	0.0002	0.003428
PYCR1	-1.62671	5.00E-05	0.001089
FGA	-1.62685	5.00E-05	0.001089
IMPA2	-1.62997	0.0001	0.001954
INHBB	-1.63015	5.00E-05	0.001089
NXPH4	-1.63201	0.003	0.026176
AKR1C1	-1.6321	5.00E-05	0.001089
TST	-1.63754	0.00065	0.008431
ZNF71	-1.63943	5.00E-05	0.001089
LOC440894	-1.64078	5.00E-05	0.001089
TRIB3	-1.64447	5.00E-05	0.001089
ITGAD	-1.64615	0.00075	0.009355
FAM194A	-1.64646	0.00095	0.011119
ZBTB32	-1.65142	5.00E-05	0.001089
NINJ1	-1.65606	5.00E-05	0.001089
RAB37	-1.66769	0.00025	0.004092
RAB3IL1	-1.66816	5.00E-05	0.001089
VWA1	-1.67644	0.0002	0.003428
KRT83	-1.67848	0.00035	0.005299
HNF1A-AS1	-1.68163	5.00E-05	0.001089
ANXA13	-1.68359	0.00035	0.005299
PIGZ	-1.68544	0.00185	0.018595
ST6GALNAC4	-1.68675	5.00E-05	0.001089
SNN	-1.68829	0.00705	0.049162
ATP6V0E2	-1.68932	0.00085	0.010312
GATSL1	-1.69282	0.00065	0.008431
PNMA6C	-1.69366	0.00125	0.013761
LOC148696	-1.6999	5.00E-05	0.001089
CEBPA-AS1	-1.7016	5.00E-05	0.001089
SLC7A7	-1.70501	5.00E-05	0.001089
DUSP23	-1.70879	0.00015	0.002736
LMO1	-1.71217	0.00565	0.041892
CYP4F3	-1.71299	5.00E-05	0.001089
LDHD	-1.71527	0.0022	0.021101
RASSF7	-1.71573	5.00E-05	0.001089
LOC283710	-1.71812	0.00565	0.041892
DPYSL2	-1.71827	5.00E-05	0.001089
SASH3	-1.71885	0.0018	0.018195
KAZALD1	-1.71888	5.00E-05	0.001089
KRT85	-1.71962	0.0016	0.016584
PROC	-1.72485	0.0002	0.003428
SLC26A1	-1.72987	0.00035	0.005299
SPDEF	-1.73698	0.0003	0.004684

PPYR1	-1.74183	5.00E-05	0.001089
PAQR4	-1.75041	5.00E-05	0.001089
HPDL	-1.75174	0.0006	0.008024
SRXN1	-1.76096	5.00E-05	0.001089
TSPAN1	-1.80224	0.00595	0.043449
LOC150381	-1.80743	0.0003	0.004684
GATA3-AS1	-1.80773	0.0053	0.040041
USP29	-1.81548	5.00E-05	0.001089
NTHL1	-1.81726	0.0001	0.001954
PRDM13	-1.81875	0.00015	0.002736
E2F2	-1.82456	5.00E-05	0.001089
AKR1C2	-1.82762	0.0002	0.003428
TUBA3E	-1.82812	0.0008	0.009825
FAM78A	-1.83541	5.00E-05	0.001089
TM4SF20	-1.8371	5.00E-05	0.001089
CRLF1	-1.84338	0.0002	0.003428
CLIC3	-1.84428	0.0019	0.019025
ABCB6	-1.86537	5.00E-05	0.001089
CRIP1	-1.86788	5.00E-05	0.001089
NUGGC	-1.86812	5.00E-05	0.001089
LINC00858	-1.87454	5.00E-05	0.001089
FZD2	-1.8765	5.00E-05	0.001089
KIF12	-1.88456	0.0002	0.003428
TRAPPC6A	-1.89131	5.00E-05	0.001089
ANKRD9	-1.9	0.0001	0.001954
CXXC5	-1.90289	5.00E-05	0.001089
CCND3	-1.90798	5.00E-05	0.001089
GLTPD2	-1.91193	0.0002	0.003428
ANKS4B	-1.91492	5.00E-05	0.001089
CBX2	-1.93137	5.00E-05	0.001089
LOC645249	-1.93621	5.00E-05	0.001089
HLA-DMB	-1.94039	0.00035	0.005299
FAM127C	-1.95469	0.00075	0.009355
MB	-1.96073	0.0007	0.008891
F7	-1.96787	0.00025	0.004092
TMEM37	-1.97285	5.00E-05	0.001089
METTL7B	-1.9842	5.00E-05	0.001089
SSR4P1	-2.00647	0.00045	0.006411
GLIPR1L1	-2.01782	0.0018	0.018195
NECAB2	-2.02746	5.00E-05	0.001089
GPRIN2	-2.04684	5.00E-05	0.001089
AKR7A3	-2.04877	0.00525	0.039739
SCTR	-2.05663	0.0005	0.006979
B3GNT1	-2.05986	5.00E-05	0.001089
CLEC11A	-2.06655	0.00055	0.007519
RNASE4	-2.0918	0.00095	0.011119

BHLHA15	-2.09775	0.00265	0.024071
C17orf50	-2.14006	0.002	0.01979
CT49	-2.1524	0.00075	0.009355
KRTAP4-1	-2.17713	0.0036	0.030077
MIR210HG	-2.18349	5.00E-05	0.001089
ATOH8	-2.19883	5.00E-05	0.001089
KLHDC7A	-2.26927	5.00E-05	0.001089
OSGIN1	-2.28319	5.00E-05	0.001089
LINC00086	-2.29224	0.00015	0.002736
LOC113230	-2.30113	5.00E-05	0.001089
EMILIN1	-2.32105	5.00E-05	0.001089
NRTN	-2.38775	0.00485	0.037622
SFRP4	-2.42583	5.00E-05	0.001089
KRT4	-2.46233	5.00E-05	0.001089
NUPR1	-2.50193	5.00E-05	0.001089
SHH	-2.52536	5.00E-05	0.001089
KRT82	-2.61671	5.00E-05	0.001089
HMOX1	-2.6431	5.00E-05	0.001089
LOC100506229	-2.66488	5.00E-05	0.001089
EMID1	-2.80838	0.0006	0.008024
DHRS4L1	-2.8223	0.00145	0.015348
CPN1	-2.91205	0.00025	0.004092
CLDND2	-2.98403	5.00E-05	0.001089

(a) probability of your false positive

(b) adjusted p-values found using an optimised False Discovery Rate (FDR) approach.

Table S3. Differentially expressed genes in BTV8ΔNS4- compared to BTV8wt-infected A549 cells.

Gene symbol	Fold change (log2)	P-value (a)	Q-value (b) (<0.05)
GBP1	3.14018	5.00E-05	0.003591
BST2	2.97403	5.00E-05	0.003591
TRIM22	2.93316	5.00E-05	0.003591
BATF2	2.93008	5.00E-05	0.003591
IFNB1	2.91208	5.00E-05	0.003591
IFNL3	2.82238	5.00E-05	0.003591
IFNL2	2.64721	5.00E-05	0.003591
CH25H	2.5473	5.00E-05	0.003591
SAMD9	2.50831	5.00E-05	0.003591
MC5R	2.50592	5.00E-05	0.003591
XAF1	2.49203	5.00E-05	0.003591
CMPK2	2.47945	5.00E-05	0.003591
IFI27	2.44843	5.00E-05	0.003591
PRR15	2.41891	5.00E-05	0.003591
IFITM1	2.37224	0.0001	0.006252
IFI6	2.34314	5.00E-05	0.003591
MX1	2.27635	5.00E-05	0.003591

LAMP3	2.25638	5.00E-05	0.003591
PARP10	2.24328	5.00E-05	0.003591
RSAD2	2.23044	5.00E-05	0.003591
HPCAL4	2.20213	0.00025	0.012378
UBE2L6	2.17791	5.00E-05	0.003591
SAMD9L	2.16761	5.00E-05	0.003591
MX2	2.14045	5.00E-05	0.003591
IFI16	2.12337	5.00E-05	0.003591
HLA-F	2.11707	0.0002	0.010489
DDX58	2.09414	5.00E-05	0.003591
IFNL4	2.0924	5.00E-05	0.003591
KCNS3	2.07421	0.0015	0.047076
NLRC5	2.03646	5.00E-05	0.003591
IFI35	2.01212	5.00E-05	0.003591
IFIT1	2.01191	5.00E-05	0.003591
TLR3	1.98784	5.00E-05	0.003591
IFIT3	1.9659	5.00E-05	0.003591
STAT2	1.94214	5.00E-05	0.003591
APOL2	1.89646	5.00E-05	0.003591
IFI27L2	1.8905	0.00065	0.024792
STAT1	1.86782	5.00E-05	0.003591
GBP3	1.83922	5.00E-05	0.003591
IFNL1	1.82874	5.00E-05	0.003591
OAS1	1.82602	5.00E-05	0.003591
TRANK1	1.81973	5.00E-05	0.003591
IFIT1B	1.79514	0.0006	0.023561
USP18	1.78937	5.00E-05	0.003591
NR1H4	1.78896	0.00015	0.008421
APOL6	1.78076	5.00E-05	0.003591
CD274	1.76901	5.00E-05	0.003591
DDX60L	1.75608	5.00E-05	0.003591
LMO2	1.73266	0.0013	0.043634
IFITM3	1.7314	0.0016	0.049245
HELZ2	1.72962	5.00E-05	0.003591
CXCL11	1.69272	0.00065	0.024792
IRF7	1.68603	5.00E-05	0.003591
PSMB9	1.68053	0.0001	0.006252
ISG15	1.67626	5.00E-05	0.003591
SP110	1.66501	5.00E-05	0.003591
IFIT2	1.66112	5.00E-05	0.003591
RAET1L	1.64732	0.0004	0.017813
SAMHD1	1.63653	5.00E-05	0.003591
C19orf66	1.62591	0.00025	0.012378
MYD88	1.62019	5.00E-05	0.003591
DTX3L	1.60771	0.00015	0.008421
NEUROD4	1.60283	0.00095	0.033959
GBP4	1.59668	5.00E-05	0.003591
OASL	1.59319	5.00E-05	0.003591
OAS3	1.59305	5.00E-05	0.003591

ULBP2	1.57916	0.0006	0.023561
DDX60	1.57736	5.00E-05	0.003591
TTC39B	1.53411	0.00065	0.024792
IFI44	1.52205	5.00E-05	0.003591
PLEKHA4	1.50765	5.00E-05	0.003591
ERAP2	1.5065	0.00015	0.008421
SEC16B	1.48137	0.0007	0.026136
TAP1	1.47697	0.00015	0.008421
TRIM21	1.449	5.00E-05	0.003591
HLA-B	1.44067	0.0002	0.010489
EGR3	1.43939	0.00025	0.012378
APOL1	1.43459	0.0002	0.010489
CFB	1.41816	0.0001	0.006252
PMAIP1	1.41097	5.00E-05	0.003591
PARP14	1.40239	0.0001	0.006252
TRIM38	1.39633	5.00E-05	0.003591
ST8SIA4	1.37122	0.0015	0.047076
IFIT5	1.36304	0.0001	0.006252
TAP2	1.3576	0.00085	0.031082
HLA-C	1.33806	0.00035	0.016081
MXD1	1.33105	5.00E-05	0.003591
TDRD7	1.32125	5.00E-05	0.003591
OTUD1	1.30353	0.00025	0.012378
B2M	1.29837	5.00E-05	0.003591
FAM46A	1.29746	0.0001	0.006252
ACER2	1.29458	0.0004	0.017813
CXCL1	1.28496	0.0003	0.014193
LAP3	1.28368	0.0001	0.006252
HERC6	1.26907	0.00025	0.012378
FLRT3	1.25725	0.00025	0.012378
TMEM47	1.24888	0.00065	0.024792
TRIM25	1.24111	0.00035	0.016081
IRF1	1.23749	0.0007	0.026136
EGR4	1.22741	0.0005	0.021145
CCL5	1.22149	0.00015	0.008421
SP100	1.21649	0.0005	0.021145
CXCL5	1.17883	0.00035	0.016081
FAM84B	1.17252	0.0003	0.014193
CXCL3	1.16635	0.00105	0.037118
THBS1	1.16608	0.00055	0.022143
HERC5	1.16438	0.00055	0.022143
PLSCR1	1.15387	0.0006	0.023561
CITED2	1.13106	0.00115	0.03926
HOMER1	1.12072	0.0007	0.026136
PARP12	1.11563	0.0015	0.047076
IRF9	1.06493	0.0012	0.04088
SAT1	1.06258	0.00045	0.019443
TRNP1	1.0546	0.00125	0.042312
RNF19B	1.04025	0.00135	0.044561

CCL2	1.02868	0.0016	0.049245
IFIH1	1.00906	0.001	0.035508
ANKRD10	-1.05471	0.00135	0.044561
PCF11	-1.07775	0.00125	0.042312
TUBB1	-1.10784	0.0014	0.045645
ZNF251	-1.11366	0.00065	0.024792
ARHGAP31	-1.12698	0.0011	0.038375
ALK	-1.18148	0.0015	0.047076
KLB	-1.18621	0.00045	0.019443
PPM1M	-1.20217	0.0013	0.043634
DDA1	-1.2065	0.0005	0.021145
DNAH1	-1.21722	0.00065	0.024792
FBXO36	-1.22026	0.00085	0.031082
FAM22D	-1.22254	0.00055	0.022143
MTSS1	-1.2297	0.0004	0.017813
ST3GAL6	-1.25324	0.00055	0.022143
GPR179	-1.25375	0.00015	0.008421
REEP1	-1.25395	0.0015	0.047076
TFAP4	-1.27801	5.00E-05	0.003591
LINC00641	-1.28385	0.0002	0.010489
MFSD4	-1.28517	0.0009	0.032463
SIGLEC16	-1.28598	0.0013	0.043634
LHFPL4	-1.29358	0.00055	0.022143
ST7L	-1.29582	0.00025	0.012378
DSCAML1	-1.29729	0.0015	0.047076
FBXW10	-1.30191	0.0002	0.010489
FAM124A	-1.314	0.00075	0.027808
MYPN	-1.31592	0.0003	0.014193
MEGF11	-1.32231	0.0005	0.021145
LOC100506548	-1.32297	0.00015	0.008421
PDZD2	-1.32397	0.00035	0.016081
UCP3	-1.32564	0.00035	0.016081
KIAA1549L	-1.32767	5.00E-05	0.003591
PCYT2	-1.34143	0.0006	0.023561
CRB2	-1.35055	0.0009	0.032463
GAL3ST1	-1.35822	0.0011	0.038375
ST5	-1.35972	0.00025	0.012378
C5AR1	-1.37098	0.00015	0.008421
LEAP2	-1.37693	0.00075	0.027808
ITGA10	-1.38094	0.0007	0.026136
TAS2R3	-1.38473	0.0003	0.014193
LOC339666	-1.38598	0.0005	0.021145
RSPH4A	-1.39652	0.00145	0.04642
RPS6KL1	-1.39958	0.00015	0.008421
MUC20	-1.39975	0.0003	0.014193
MTMR9LP	-1.40239	0.00065	0.024792
MAPT	-1.40554	0.00025	0.012378
RAB11FIP4	-1.40711	0.00015	0.008421
SHISA4	-1.40904	0.00055	0.022143

ESR1	-1.40949	0.00025	0.012378
ADAM32	-1.41154	0.0007	0.026136
C11orf91	-1.41548	0.0002	0.010489
TRIM3	-1.41992	5.00E-05	0.003591
PON1	-1.42133	0.00135	0.044561
ABAT	-1.42346	0.0006	0.023561
MGC16275	-1.43091	5.00E-05	0.003591
PPP1R3F	-1.43362	0.0003	0.014193
CAND2	-1.43519	5.00E-05	0.003591
PITPNM3	-1.43662	0.00035	0.016081
SUSD5	-1.44196	0.0004	0.017813
MTHFD2L	-1.44625	0.00115	0.03926
ZSCAN5A	-1.44731	5.00E-05	0.003591
CCDC17	-1.44944	0.0004	0.017813
BAAT	-1.45095	0.00025	0.012378
LYST	-1.45179	5.00E-05	0.003591
LDHAL6A	-1.46186	0.00015	0.008421
HCRTR1	-1.46494	0.00135	0.044561
DDAH2	-1.46511	0.00015	0.008421
FAP	-1.47516	0.00055	0.022143
SLC10A1	-1.47668	5.00E-05	0.003591
WDR66	-1.47987	5.00E-05	0.003591
LOC643669	-1.48364	0.00055	0.022143
PYCARD	-1.48452	0.00045	0.019443
ANKLE1	-1.48668	0.0002	0.010489
LOC100499194	-1.48854	0.0002	0.010489
MMP14	-1.48985	0.0001	0.006252
SLC23A3	-1.49316	0.0006	0.023561
TAS2R5	-1.4989	0.00045	0.019443
NID1	-1.49895	5.00E-05	0.003591
ARHGEF4	-1.4994	0.0006	0.023561
DDN	-1.49992	0.0006	0.023561
SPRED3	-1.49997	5.00E-05	0.003591
CASQ1	-1.50168	0.00045	0.019443
CSPG5	-1.50811	0.00115	0.03926
PKI55	-1.51262	5.00E-05	0.003591
SLC16A12	-1.52004	5.00E-05	0.003591
RASD1	-1.52363	5.00E-05	0.003591
CRABP2	-1.52739	0.00035	0.016081
NID2	-1.53418	0.00015	0.008421
FES	-1.53544	0.00065	0.024792
IQSEC3	-1.53851	0.0016	0.049245
LGALS4	-1.53916	0.0009	0.032463
LINC00628	-1.54094	0.0003	0.014193
GUCA1B	-1.54221	5.00E-05	0.003591
CELF6	-1.5441	0.00065	0.024792
GTF2IRD2B	-1.54634	5.00E-05	0.003591
IFFO1	-1.54997	0.0001	0.006252
ZP1	-1.55016	0.0011	0.038375

ADSSL1	-1.55289	0.00045	0.019443
PRICKLE1	-1.55631	5.00E-05	0.003591
CXorf36	-1.55777	0.00055	0.022143
SLC5A5	-1.55867	0.0008	0.029456
LTBP2	-1.56407	5.00E-05	0.003591
ALDOB	-1.56552	0.0011	0.038375
SOWAHD	-1.56681	0.0015	0.047076
MYH3	-1.569	5.00E-05	0.003591
ATCAY	-1.56959	0.0002	0.010489
EPX	-1.57427	0.00095	0.033959
PYGM	-1.57538	0.0001	0.006252
LYPD3	-1.57618	0.0016	0.049245
ZNF501	-1.57851	0.00115	0.03926
GPD1	-1.581	5.00E-05	0.003591
CAV3	-1.59185	0.001	0.035508
LOC149134	-1.59973	0.00115	0.03926
SMLR1	-1.60083	0.0003	0.014193
C2orf83	-1.60125	0.0004	0.017813
NLRP9	-1.60702	0.00015	0.008421
MASP2	-1.61087	5.00E-05	0.003591
FAM180B	-1.61631	0.0015	0.047076
SGPP2	-1.61748	0.00035	0.016081
UBQLNL	-1.62322	0.00065	0.024792
MARK2P9	-1.63112	0.0016	0.049245
VIL1	-1.64454	0.00115	0.03926
ART1	-1.64678	0.0013	0.043634
SERPINA1	-1.65642	0.0003	0.014193
KRBA2	-1.65652	5.00E-05	0.003591
SLC6A13	-1.65845	0.0002	0.010489
SLC4A9	-1.66229	0.0007	0.026136
FHDC1	-1.66261	5.00E-05	0.003591
EXOC3L2	-1.66411	0.00015	0.008421
HIPK4	-1.66454	0.00045	0.019443
FAM167B	-1.67388	0.00155	0.048359
GFRA3	-1.67579	0.0011	0.038375
NHLH1	-1.67813	5.00E-05	0.003591
LOC100128288	-1.67966	5.00E-05	0.003591
NODAL	-1.68085	0.0001	0.006252
LOC646862	-1.68565	0.00115	0.03926
MURC	-1.69227	5.00E-05	0.003591
PCDH12	-1.69838	5.00E-05	0.003591
ADAM20	-1.70147	0.0005	0.021145
PDE2A	-1.70258	0.0001	0.006252
ZMYND10	-1.70297	0.0004	0.017813
LINC00163	-1.70594	0.0002	0.010489
TBR1	-1.71151	0.00045	0.019443
CAPN3	-1.71331	0.0009	0.032463
HCLS1	-1.71651	0.0002	0.010489
SNCG	-1.7177	0.0005	0.021145

CRHR2	-1.71917	0.0001	0.006252
SLC39A2	-1.72367	0.00065	0.024792
AOC4	-1.7238	0.00025	0.012378
FER1L5	-1.72491	0.00015	0.008421
TMEM236	-1.72606	0.00085	0.031082
COL8A2	-1.72974	0.0001	0.006252
CCDC62	-1.73071	5.00E-05	0.003591
ACRBP	-1.73858	0.0002	0.010489
AKR7L	-1.74878	0.00025	0.012378
PLBD1	-1.75119	0.00055	0.022143
TIGIT	-1.75137	0.0001	0.006252
TRIM72	-1.75928	0.00135	0.044561
SLC25A34	-1.76067	5.00E-05	0.003591
NLRP7	-1.76236	0.00105	0.037118
KCNB1	-1.76652	0.0016	0.049245
MYO15A	-1.76724	5.00E-05	0.003591
MYH15	-1.76983	5.00E-05	0.003591
FOXD4	-1.77213	5.00E-05	0.003591
ALPK3	-1.78517	5.00E-05	0.003591
LOC100505495	-1.78565	5.00E-05	0.003591
NPR2	-1.78636	5.00E-05	0.003591
TAS2R9	-1.79231	0.0009	0.032463
CCDC19	-1.79389	0.00065	0.024792
RASGRP2	-1.79443	0.0009	0.032463
LRIT3	-1.79761	0.0014	0.045645
LILRB3	-1.79809	5.00E-05	0.003591
HRH3	-1.80375	0.00055	0.022143
CLEC18A	-1.80823	0.0002	0.010489
GPR4	-1.8096	0.0003	0.014193
PIGZ	-1.81834	0.0007	0.026136
FLJ33360	-1.81885	0.0015	0.047076
FAM90A1	-1.8307	0.00115	0.03926
FAM154B	-1.83109	0.00015	0.008421
FOXD4L2	-1.84361	5.00E-05	0.003591
CNTD2	-1.848	5.00E-05	0.003591
KRT8P41	-1.84994	0.0001	0.006252
TEX35	-1.85397	0.0004	0.017813
NKAPL	-1.85588	0.0001	0.006252
CATSPERD	-1.85834	0.0003	0.014193
C6orf25	-1.85954	5.00E-05	0.003591
RSPH6A	-1.86814	0.00015	0.008421
CALHM1	-1.86841	5.00E-05	0.003591
KIF17	-1.86884	5.00E-05	0.003591
CYP26A1	-1.87083	0.0001	0.006252
LZTS1	-1.87139	5.00E-05	0.003591
B3GNT4	-1.87676	5.00E-05	0.003591
CRYBA1	-1.8849	0.0003	0.014193
LILRA6	-1.88617	5.00E-05	0.003591
SEMA4A	-1.88851	0.0004	0.017813

ZNF610	-1.88998	0.0004	0.017813
TINCR	-1.89323	5.00E-05	0.003591
SBSN	-1.89499	0.00055	0.022143
TCAP	-1.9031	0.0002	0.010489
MFRP	-1.91084	0.00025	0.012378
SOST	-1.91165	0.0014	0.045645
RNF183	-1.9118	0.0005	0.021145
LY6G5C	-1.91304	0.0002	0.010489
SSUH2	-1.92319	5.00E-05	0.003591
KBTBD13	-1.92535	5.00E-05	0.003591
C7orf61	-1.92632	0.00115	0.03926
FAM221B	-1.93557	0.00055	0.022143
APOE	-1.93567	5.00E-05	0.003591
PYROXD2	-1.94141	0.00025	0.012378
RDH12	-1.94492	0.0002	0.010489
KLHL35	-1.94592	0.0002	0.010489
RYR1	-1.9461	5.00E-05	0.003591
MTUS2	-1.94815	5.00E-05	0.003591
FAM163A	-1.95539	0.00055	0.022143
SFRP5	-1.97419	5.00E-05	0.003591
RASL10B	-1.97696	5.00E-05	0.003591
SNX29P2	-1.9806	0.0001	0.006252
CD5	-1.98655	0.00015	0.008421
CCDC11	-2.00638	5.00E-05	0.003591
POU2F2	-2.01441	5.00E-05	0.003591
LOC100130705	-2.02375	5.00E-05	0.003591
SLC6A4	-2.0277	5.00E-05	0.003591
PNLDC1	-2.03458	0.00015	0.008421
LOC728175	-2.03474	0.00055	0.022143
TG	-2.03992	5.00E-05	0.003591
MSH4	-2.04681	5.00E-05	0.003591
VWCE	-2.05176	0.0001	0.006252
KLK14	-2.05257	0.00135	0.044561
IL18RAP	-2.05367	0.0014	0.045645
HAPLN4	-2.05942	0.0007	0.026136
LPAR5	-2.06179	0.0004	0.017813
LINC00654	-2.06492	5.00E-05	0.003591
F13B	-2.0664	0.00055	0.022143
GPR151	-2.07033	0.0001	0.006252
TRABD2A	-2.07601	5.00E-05	0.003591
HAVCR2	-2.08154	5.00E-05	0.003591
CILP2	-2.09392	5.00E-05	0.003591
FOXD4L5	-2.0978	0.0001	0.006252
SLC25A42	-2.10545	5.00E-05	0.003591
VWA3A	-2.11576	0.00015	0.008421
EFCAB12	-2.12151	0.0008	0.029456
NRADDP	-2.12868	0.00015	0.008421
LOC100128770	-2.1303	5.00E-05	0.003591
GTF2IRD2P1	-2.14051	0.0001	0.006252

TRPC7	-2.1475	5.00E-05	0.003591
FAM83C	-2.15004	5.00E-05	0.003591
SLC6A12	-2.15199	5.00E-05	0.003591
GOLGA6C	-2.16094	0.00015	0.008421
FBLL1	-2.1685	5.00E-05	0.003591
MYO1A	-2.17486	5.00E-05	0.003591
FUT3	-2.1785	0.0003	0.014193
FOXD4L6	-2.19147	5.00E-05	0.003591
MYOZ3	-2.19159	5.00E-05	0.003591
GPBAR1	-2.19196	5.00E-05	0.003591
HAPLN2	-2.19291	5.00E-05	0.003591
ACTN3	-2.19699	5.00E-05	0.003591
RFPL3S	-2.20766	5.00E-05	0.003591
OVGP1	-2.21362	5.00E-05	0.003591
SLC45A2	-2.21882	0.0001	0.006252
LMOD1	-2.2351	5.00E-05	0.003591
PTAFR	-2.23785	5.00E-05	0.003591
TSPAN1	-2.24287	0.0015	0.047076
TRHR	-2.25007	0.0001	0.006252
SCN5A	-2.25198	5.00E-05	0.003591
DQX1	-2.27897	5.00E-05	0.003591
PAX6	-2.28336	5.00E-05	0.003591
PTH1R	-2.29979	0.0001	0.006252
GTF2IRD2	-2.30771	5.00E-05	0.003591
MMRN2	-2.30835	5.00E-05	0.003591
FGF23	-2.30899	5.00E-05	0.003591
MAGEA8	-2.31811	5.00E-05	0.003591
ARL9	-2.32698	5.00E-05	0.003591
ANKK1	-2.32707	5.00E-05	0.003591
IL13	-2.34279	0.0001	0.006252
C3orf20	-2.34809	5.00E-05	0.003591
PROZ	-2.34853	5.00E-05	0.003591
NPC1L1	-2.36663	5.00E-05	0.003591
ASGR1	-2.36795	5.00E-05	0.003591
PRRT1	-2.37736	0.00095	0.033959
N4BP2L1	-2.38521	5.00E-05	0.003591
SFTPA1	-2.38557	5.00E-05	0.003591
GPR84	-2.38783	0.00015	0.008421
RAPSN	-2.39433	5.00E-05	0.003591
TRIM40	-2.40237	0.00035	0.016081
TPRX1	-2.40584	0.0001	0.006252
FOXD4L1	-2.42932	5.00E-05	0.003591
WNT2B	-2.43211	5.00E-05	0.003591
NFE2	-2.43301	5.00E-05	0.003591
IL17RE	-2.43853	5.00E-05	0.003591
LOC100507140	-2.44036	0.00125	0.042312
SLC28A1	-2.44199	0.0002	0.010489
LECT1	-2.44463	0.0016	0.049245
FAM109B	-2.45378	0.00015	0.008421

FAM209B	-2.45637	5.00E-05	0.003591
SPTSSB	-2.45911	0.00095	0.033959
NAT8	-2.46647	0.0011	0.038375
SPACA4	-2.46924	0.0006	0.023561
MPP4	-2.47138	0.0003	0.014193
EPPK1	-2.47763	5.00E-05	0.003591
ETV2	-2.48924	5.00E-05	0.003591
LOC100507206	-2.49054	0.00015	0.008421
RSPH10B2	-2.51123	0.00035	0.016081
LHX3	-2.52268	0.00045	0.019443
MMP28	-2.52639	0.00025	0.012378
CYP2A7	-2.5276	0.00045	0.019443
TLR9	-2.53418	5.00E-05	0.003591
TLX2	-2.53958	5.00E-05	0.003591
LY6G6C	-2.54466	5.00E-05	0.003591
CPT1C	-2.56802	5.00E-05	0.003591
PKD1L1	-2.57102	5.00E-05	0.003591
MMP25	-2.5829	5.00E-05	0.003591
TTC24	-2.58564	5.00E-05	0.003591
ADCY4	-2.58649	5.00E-05	0.003591
CHRM1	-2.59838	5.00E-05	0.003591
LRRC66	-2.6013	5.00E-05	0.003591
LMOD2	-2.60341	5.00E-05	0.003591
ACTA1	-2.60905	5.00E-05	0.003591
FNDC7	-2.61593	5.00E-05	0.003591
HK3	-2.61841	5.00E-05	0.003591
KIAA1045	-2.62861	5.00E-05	0.003591
SLC7A9	-2.63095	5.00E-05	0.003591
NEB	-2.64404	5.00E-05	0.003591
SCUBE2	-2.65382	5.00E-05	0.003591
CYP2G1P	-2.65475	5.00E-05	0.003591
OLFML2B	-2.65876	5.00E-05	0.003591
LRRC10	-2.68089	5.00E-05	0.003591
LOC153910	-2.70534	5.00E-05	0.003591
FOXD4L3	-2.70811	5.00E-05	0.003591
EFNA2	-2.70858	5.00E-05	0.003591
AOC3	-2.72256	5.00E-05	0.003591
OIT3	-2.73746	5.00E-05	0.003591
DHDH	-2.74564	5.00E-05	0.003591
GRK7	-2.7544	0.0001	0.006252
FAM83E	-2.76319	5.00E-05	0.003591
RPRML	-2.76673	5.00E-05	0.003591
BST1	-2.76895	5.00E-05	0.003591
TRPM5	-2.7777	5.00E-05	0.003591
LTA	-2.78835	5.00E-05	0.003591
SYT5	-2.78955	5.00E-05	0.003591
LGR5	-2.79614	5.00E-05	0.003591
KLRK1	-2.79968	0.0014	0.045645
GOLGA6A	-2.81302	0.0001	0.006252

FOXS1	-2.81649	5.00E-05	0.003591
SV2A	-2.82714	5.00E-05	0.003591
TAS2R7	-2.82854	0.00075	0.027808
WNT8B	-2.83173	0.00025	0.012378
S100A5	-2.84252	5.00E-05	0.003591
ARL10	-2.84528	5.00E-05	0.003591
MYOC	-2.86007	0.00045	0.019443
CTRC	-2.86048	0.0008	0.029456
POU1F1	-2.8657	0.0007	0.026136
C6orf222	-2.88514	5.00E-05	0.003591
OVOL1	-2.92359	5.00E-05	0.003591
PDLIM4	-2.92507	0.0003	0.014193
EPHA8	-2.96804	5.00E-05	0.003591
SHD	-2.97102	5.00E-05	0.003591
C1orf189	-2.98218	5.00E-05	0.003591
GUCY2D	-2.98813	0.0002	0.010489
OXER1	-3.01213	5.00E-05	0.003591
FUT5	-3.02069	0.0006	0.023561
C1orf162	-3.03857	5.00E-05	0.003591
FAM71F1	-3.10421	5.00E-05	0.003591
CASS4	-3.10627	5.00E-05	0.003591
SLC2A9	-3.14108	0.00055	0.022143
GADL1	-3.14387	0.0001	0.006252
CACNG1	-3.14573	0.0001	0.006252
POPDC2	-3.14889	5.00E-05	0.003591
MRPL23-AS1	-3.17037	5.00E-05	0.003591
RNF112	-3.18358	5.00E-05	0.003591
ESRRB	-3.18373	0.00015	0.008421
GPR152	-3.20663	5.00E-05	0.003591
MTNR1A	-3.24022	5.00E-05	0.003591
GOLGA6B	-3.26519	0.00025	0.012378
GAS2L2	-3.26743	5.00E-05	0.003591
LY6G6D	-3.29162	0.00135	0.044561
CD79A	-3.29471	5.00E-05	0.003591
ITGA9	-3.39668	5.00E-05	0.003591
C13orf45	-3.40161	0.00055	0.022143
PLA2G12B	-3.45431	0.00055	0.022143
IRGC	-3.51001	0.00015	0.008421
GSTM2P1	-3.56487	5.00E-05	0.003591
C19orf69	-3.62239	0.00155	0.048359
CYP2A6	-3.64568	0.0001	0.006252
TSGA10IP	-3.68956	5.00E-05	0.003591
MOBP	-4.02337	5.00E-05	0.003591
HPX	-4.13135	5.00E-05	0.003591
LRRC43	-4.15833	0.00015	0.008421

[a] probability of your false positive

[b] adjusted p-values found using an optimised False Discovery Rate (FDR) approach.
